# In-depth characterization of minor 2-(2-phenylethyl)chromone oligomers from Chinese agarwood by integrating offline two-dimensional liquid chromatography and hybrid ion trap time-of-flight mass spectrometry

**DOI:** 10.1186/s13020-025-01073-6

**Published:** 2025-02-27

**Authors:** Huixia Huo, Hang Zhang, Huiting Liu, Jiale Ma, Qian Zhang, Yunfang Zhao, Jiao Zheng, Pengfei Tu, Yuelin Song, Jun Li

**Affiliations:** 1 Modern Research Center for Traditional Chinese Medicine, Beijing Research Institute of Chinese Medicine, Beijing University of Chinese Medicine, Beijing, 102488 People’s Republic of China; 2 School of Chinese Materia Medica, Beijing University of Chinese Medicine, Beijing, 102488 People’s Republic of China

**Keywords:** Chinese agarwood, Offline two-dimensional liquid chromatography, 2-(2-Phenylethyl)chromone dimers, Minor compounds

## Abstract

Those minor, even trace natural products sometimes exhibit exciting activities and possess unique structures; however, it is challenging to pursue and identify such components using routine LC−MS/MS platforms attributing to their low distribution levels in herbs, the overlapping effects from the abundant ingredients and the high-level structural diversity. Here, an off-line two-dimensional liquid chromatography hook up hybrid ion trap time-of-flight mass spectrometry program was exploited to facilitate the exposure of those minor components in chromatographic domain and to acquire high-resolution multi-stage mass spectra, and the less abundant 2-(2-phenylethyl)chromone (PEC) oligomers from Chinese agarwood that is one of the most precious herbal medicines were concerned to illustrate and assess the applicability towards capturing and structurally annotating those minor components. The mass fragmentation pathways of PEC dimers, in particular the linkage fission between monomers, were proposed by assaying eighteen authentic compounds that covered different conjugation manners, and subsequently applied for the tentative structural identification of observed components. Thereafter, targeted purification was conducted to generate eight new, trace PEC dimers to justify the annotated structures. As a result, heterocyclic ring fission was the diagnostic fragmentation pathways for PEC dimers. In total, 199 PECs were discovered and characterized, consisting of 74 dimers and five trimers. Noteworthily, after structural identification with NMR assays, the confirmative structures of those eight new PEC dimers agreed well with the identities suggested by mass fragmentation rules. Above all, PEC derivatives, notably trace oligomers, in Chinese agarwood were profiled in depth, resulting in a number of interesting structures.

**Supplementary Information:**

The online version contains supplementary material available at 10.1186/s13020-025-01073-6.

## Introduction

Increasing evidences have demonstrated that traditional Chinese medicines (TCMs) own promising therapeutical benefits for the treatment of diverse diseases, such as COVID-19 syndrome, tumor, Alzheimer's disease, etc. [1–3] The chemical diversity of TCM lays the foundation for its therapeutical outcomes for those complex, multifactorial diseases, yet presents a formidable task for the chemical composition clarification. LC–MS/MS has undoubtedly emerged as the pivotal tool for the structural identification of constituents in TCM extracts [4–6]. Among the available approaches, the hybrid ion trap time-of-flight mass spectrometer (IT-TOF–MS) seamlessly integrates the mass spectrometric capabilities of the ion trap and time-of-flight mass device, leading to a unique ability to record high-resolution MS^n^ (*n* = 1, 2, 3 …) spectra. As a result, IT-TOF–MS has garnered widespread acclaim as an eminently suitable instrument for deep-going chemical profiling of intricate matrices, *e.g.*, herbal medicine [7–9]. Although LC–MS offers its benefits, it still faces certain limitations for comprehensively elucidating the complex chemical makeup of herbal medicines. First, in most cases, routine LC–MS measurement shows insufficient peak capacity, and those minor but crucial constituents are usually co-eluted with major components on a single column [10, 11]. Secondly, during MS analysis, minor components often encounter ion-suppression effect resulted from their high abundance counterparts. Thirdly, the vast chemical diversity, coupled with the scarcity of commercially available standards and the resemblance fragmentation patterns among different classes, collectively conspire to render unambiguous identification of compounds infeasible without chromatographic resolution. Therefore, enhanced chromatographic resolution is imperative for conducting an in depth the chemical constituents of complicated herbal medicine, and to discover novel trace compounds.

Two-dimensional LC (2D LC) has become a promising choice to fulfill the chromatographic requirements of complex samples due to the advantages in regards of selectivity, peak capacity, and resolution compared with the single column LC [10–12]. 2D LC arises from the innovative combination of two distinct yet independent separation processes, enabling a given compound in sample undergoes two disparate separation mechanisms, resulting in a significant expanding of peak capacity. A 2D LC system has the capability to be configured for versatile applications, accommodating either an off-line or an on-line operational mode, each offering unique advantages tailored to specific analytical separations. In the off-line configuration, 2D LC stands out for its ability to perform sample separation in both dimensions using entirely independent elution programs, leading to a greater convenience to select mobile phase and chromatographic mechanism, than an on-line system [13]. More importantly, the peak capacity of online 2D LC, particularly those designed for comprehensive 2D LC, is dramatically narrowed by the limited measurement time available for the second separation dimension; however, the measurement time of either dimension can be flexibly set as demanded, resulting in a further improvement of peak capacity.

Agarwood consists of the resinous heartwoods of *Aquilaria* tree, a member of the Thymelaeaceae family. *Aquilaria sinensis* (Lour). Gilg is the sole officially recognized source of Chinese agarwood in Chinese Pharmacopoeia (2020 Edition), which primarily found in South China and North Vietnam [14]. The research have indicated that 2-(2-phenylethyl)chromones (PECs) and sesquiterpenoids are the principal effective constituents in Chinese agarwood [15–18]. PECs, notably the highly oxidized form namely 5,6,7,8-tetrahydro-2-(2-phenylethyl)chromones, have been demonstrated the unique distribution in agarwood and could be utilized as the diagnostic markers for authenticity and quality control. Recently, PECs have been reveals as the principal roles being responsible for the anti-inflammatory and gastroprotective properties of Chinese agarwood [19, 20]. In our previous study, thirty-three PEC dimers were gained and characterized from Chinese agarwood, with the majority exhibiting significant anti-inflammatory activities [21, 22]. Moreover, this chemical cluster has been proved to play important roles towards the warm, pleasant, balsamic, and long-lasting odor, and their accumulation can be simulated by burning or artificial heat [15]. Therefore, it is of great importance to pursue in depth PECs from agarwood through assaying with the cutting-edge analytical techniques.

**Fig. 1:**
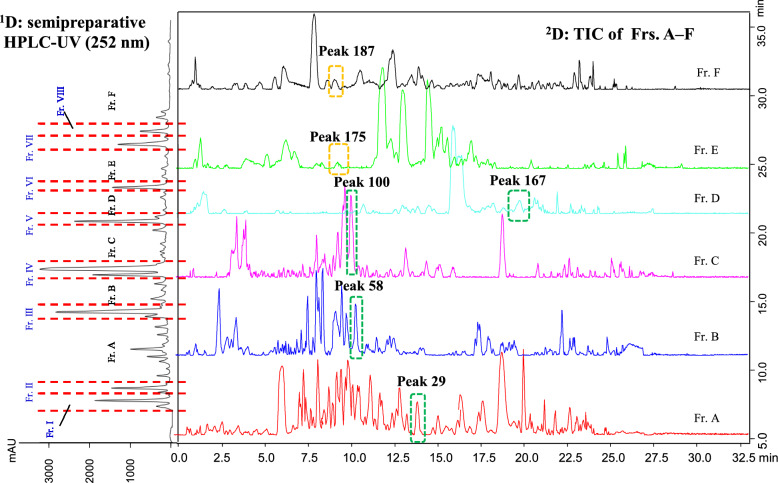
The off-line ^1^D semipreparative HPLC–UV (252 nm)-^2^D total ion current (TIC) chromatograms of selected Frs. A–F

In the present study, in order to comprehensively understand the profile of PECs in agarwood, we attempted to integrate offline 2D LC, IT-TOF–MS, and NMR. Firstly, we conducted semi-preparative LC along with a self-prepared automated fraction collection module to partition the entire extract into eight apparently pure compounds, namely Frs. I–VIII, by purposely collecting those primary peaks, together with six fractions, such as Frs. A–F, through successively collecting eluates with appropriate program. Then, the fractions comprising minor components were further subjected onto Ascentis® Express F5 column and monitored by IT-TOF MS (Fig. 1). In total, 199 PECs were discovered and preliminarily characterized, including 74 PEC dimers and five PEC trimers. Eight targeted unknown minor PEC dimers were isolated from agarwood to justify the reliability of MS-oriented structural elucidation.

## Materials and methods

### Chemicals and reagents

Authentic compounds, crassin A (**1**), aquisinenone N (**2**), aquisinenone G (**3**), 4′-methoxy-aquisinenone G (**4**), aquisinenone A (**5**), 4′-methoxyaquisinenone A (**6**), aquisinenone B (**7**), 6′′-hydroxyaquisinenone B (**8**), 6′′-hydroxy-4′,4′′′-dimethoxyaquisinenone B (**9**), aquisinenone C (**10**), aquisinenone D (**11**), 4′-demethoxyaquisinenone D (**12**), aquisinenone E (**13**), aquisinenone F (**14**), 4′,4‴-dimethoxyaquisinenone K (**15**), aquisinenone I (**16**), 7″-methoxyaquisinenone I (**17**), and 4′,7″-dimethoxyaquisinenone I (**18**) were previously isolated by the authors from Chinese agarwood (Fig. S1) [21, 22]. The purity of each compound, assessed through HPLC–DAD analysis, was confirmed to exceed 98% by NMR spectroscopy.

All LC–MS grade reagents were supplied by Thermo-Fisher (Pittsburgh, PA, USA). In-house, de-ionized water was provided utilizing a Milli-Q Purifier (Millipore, Bedford, MA, USA).

Sixteen batches of Chinese agarwood (Asi 1–16), in total, were gathered from the provinces of Hainan and Guangdong in China. Professor Pengfei Tu confirmed that all materials hailed from *Aquilaria sinensis*. All voucher specimens have been systematically archived at our institute (Beijing, China).

### Sample preparation

To prepare the stock solutions, eighteen PEC dimers (0.5–1.0 mg) were individually dissolved in 1 mL of methanol and subsequently filtered through 0.22 μm nylon membrane filters sourced from Jinteng Experiment Equipment Co. Ltd. (Tianjin, China).

Aliquots (200 mg for each batch) of Asi 1–16 were thoroughly mixed, and the ultrasonication (ultrasonicator, 100 W, Fisher Scientific, USA) with 50 mL methanol was performed at 25 °C for 30 min. The extract was concentrated to dryness under reduced pressure and then reconstituted with methanol. Subsequently, the reconstituted solution was leached through a 0.22 µm membrane and stored at 4 °C till use [23].

### Offline 2D LC separation

The first-dimensional (^1^D) RP separation was conducted on a semipreparative YMC-Pack C_18_ column (10 × 250 mm, 5 μm, Kyoto, Japan) utilizing a Shimadzu LC-20AD modular system (Kyoto, Japan). The gradient elution was programmed using a mobile phase comprising of water (A) and ACN (B) as follows: 0–30 min, 35%–95% B; 30–36 min, 95% B. The flow rate was adjusted to 2 mL/min. Those eight principal peaks were purposely collected to generate eight apparently pure fractions, namely Frs. I–VIII, and moreover, six another fractions, namely Frs. A–F, were gained by following the program illustrated in Fig. 1, and noteworthily, an automated collection module configured previously in our group [23] was applied to accomplish the eluate collection task. The obtained principal peaks and fractions were meticulously dried under a constant flow of N_2_ at 37 °C. Afterwards, eight principal peaks were parallel measured by ^1^H-NMR and LC–IT-TOF–MS to afford a total of nine PEC derivatives following the protocols reported in our previous study [23]. Dry residues of six fractions were reconstituted with methanol and individually filtered through 0.22 μm membranes prior to analysis.

The second dimension was conducted on a Shimadzu Nexera XR UHPLC system (Tokyo, Japan). An Ascentis® Express F5 (150 × 2.1 mm, 2.7 μm) column maintained at 40 °C was used. The mobile phase consisted of 0.1% formic acid (A) and ACN (B), with the following elution procedure: 0–18 min, 5%–30% B; 18–21 min, 30%–95% B; 21–23 min, 95% B for Fr.A; 0–18 min, 10%–45% B; 18–21 min, 45%–95% B; 21–23 min, 95% B for Fr.B; 0–18 min, 25%–40% B; 18–21 min, 40%–95% B; 21–23 min, 95% B for Fr.C; 0–18 min, 25–45% B; 18–21 min, 45–95% B; 21–23 min, 95% B for Fr.D; 0–18 min, 25–50% B; 18–21 min, 50–95% B; 21–23 min, 95% B for Fr.E; 0–18 min, 30–60% B; 18–21 min, 60–95% B; 21–23 min, 95% B for Fr.F. The flow rate and injection volume were kept constant at 0.5 mL/min and 5 *μ*L, respectively. The total analysis time was 30 min for a single chromatographic run. The outlet of Ascentis® Express F5 column was sequentially attached to DAD module (UV = 252 nm) and IT-TOF–MS.

### IT-TOF–MS analysis

The work solution and the outlet of the Ascentis® Express F5 column was directed into a IT-TOF–MS for tandem HRMS data acquisition, the crucial parameters setting as the descriptions in our previous study [7, 24]. Positive mass spectra were systematically acquired in the full scan and automatic multiple stage fragmentation scan modes, with all MS^1^, MS^2^, and MS^3^ spectra collected in the range of *m/z* 50–1000. The ion accumulation time and collision energy of CID was adjusted to 100 ms and 50%, respectively. The LCMS solution software package (version 1.1) from Shimadzu facilitated efficient data acquisition and processing. The precision of the assigned chemical formula was assessed with a mass difference tolerance of ± 20 ppm, calculated from the deviation between the experimental and theoretical masses.

### Purification of new PEC dimers

To verify the reliability of MS-oriented structural elucidation, eight new PEC dimers were isolated by an HPLC–MS guided fractionation procedure. The details of the phytochemical isolation of new PEC dimers and the general experimental procedures are described in the Supporting Information.

## Results

### Method optimization: Selection of the column systems

To exploit an efficient strategy for enriching and analyzing of minor components present in agarwood extracts is the objective of this work. Given the intricate nature of the chemical composition, extensive concentration range, and the structural similarity among PECs, it was virtually infeasible to exactly achieve successful holistic and in-depth understanding of the micro-components in conventional 1D chromatographic modes. Compared to 1D-LC, a 2D-LC system, which arises from the integration of two distinct separation processes, is exceptionally proficient for the analysis of TCM and metabonomics [12]. In practice, a 2D-LC encompasses versatile operational modes, including offline, online, and a stop-flow strategy. In off-line modes, fractions obtained from the 1D are manually gathered, subjected to appropriate treatment, and then injected in the 2D. This approach can employ extended analysis times in the 2D to allow maximum utilization of each of the 1D separations [25]. Therefore, an offline 2D LC coupled with IT-TOF–MS was applied for comprehensive characterization of PECs. In the work, to enrich the micro-components, the entire extract was fragmented into eight principal peaks [23] and six fractions (Fig. 1).

To construct a practical 2D LC system that obtains satisfactory separation for the minor, we refined the chromatographic parameters, encompassing the selection of chromatographic column, mobile phase, and gradient elution program. The primary consideration of picking a column was to gain a high level of orthogonality obtained from the semipreparative RPLC separation used in the 1D. To improve the peak capacity of system, several columns were tried, such as Waters Xbridge Amide (150 × 4.6 mm, 3.5 μm), Ascentis® Express F5 (150 × 2.1 mm, 2.7 μm), ACE UltraCore 2.5 SuperC18 (150 × 3.0 mm, 2.5 µm), Diamonsil C_18_ (250 × 4.6 mm, 5 µm), Capcell PAK C_18_ (250 × 4.6 mm, 5 µm), Phenomenex Kinetex C18 (100 × 3.0 mm, 2.6 µm), and Waters Acquity UPLC HSS T3 (100 × 2.1 mm, 1.8 µm). Finally, Ascentis® Express F5 column was employed due to its dual-mode retention behavior (reversed-phase and HILIC retention modes), especially for polar compounds, than the other candidates, for instance, Phenomenex Kinetex C18 and Waters Acquity UPLC HSS T3. To enhance the chromatographic behaviors, the constitution of mobile phase was screened between water–methanol and water–CAN. Subsequently, the column temperatures (25, 30, and 40 °C) were also screened. Ultimately, the water–acetonitrile system at a column temperature of 40 °C exhibited superior separation efficiency for all analytes. To further refine peak profiles of the target compounds and augment resolution, 0.1% formic acid was employed as a modifier in the water phase. Afterward, the gradient elution program was thoroughly screened to attain satisfactory separation.

### Structural elucidation of PECs by LC/MS

A total of 199 PECs were effectively discovered and tentatively characterized from the extracts of agarwood by using the 2D LC system and analyzed by using UHPLC-IT-TOF/MS. Those PECs obtained in agarwood were divided into several subtypes, such as PECs of flindersia type (FTPECs), 5,6,7,8-tetrahydro-2-(2-phenylethyl)chromones (THPECs), 5,6:7,8-diepoxy-2-(2-phenylethyl)chromones (DEPECs), mono-epoxy-2-(2-phenylethyl)chromones (EPECs), PEC dimers, and PEC trimers. The MS analysis was conducted in positive ion mode due to its greater sensitivity and responsiveness towards the analytes being investigated. Compounds were tentatively identified through a combination of accurate mass measurements, predicted molecular formulae, and a comparative analysis of UV-, MS spectra, and primary fragmentation patterns compared to the data archived in the literature.

### Identification of FTPECs (I) in agarwood

PEC is distinguished as a category of chromones marked by the presence of a phenylethyl moiety at the C-2 position. Typically, PECs possess some common substituents such as hydroxy and methoxy. While chlorine substitution and PEC glycosides do occur in agarwood, they are encountered with relatively rare frequencies. The fragmentation pathway of FTPECs have been proposed in the literature [26, 27], and the ^6^A^+^ and ^6^B^+^ ions originated from the cleavages of the CH_2_–CH_2_ (C_7′_–C_8′_) bond connecting chromone moiety and phenyl moiety were typically produced as the diagnostic signal (Fig. S2B, Supporting Information). Therefore, distinct phenyl moieties produce characteristic fragment ions like *m/z* 91 [^6^B]^+^ ( C_7_H_7_), *m/z* 107 [^6^B + OH]^+^ (C_7_H_6_ + OH), *m/z* 121 [^6^B + OCH_3_]^+^(C_7_H_6_ + OCH_3_), and *m/z* 137 [^6^B + OCH_3_ + OH]^+^ (C_7_H_6_ + OCH_3_ + OH), whereas various substituted chromone moieties generate ions, such as *m/z* 161 [^6^A]^+^ (C_10_H_8_O_2_), *m/z* 177 [^6^A + OH]^+^ (C_10_H_8_O_2_ + OH), *m/z* 191 [^6^A + OCH_3_]^+^ (C_10_H_8_O_2_ + OCH_3_), *m/z* 207 [^6^A + OCH_3_ + OH]^+^ (C_10_H_8_O_2_ + OCH_3_ + OH), *m/z* 191 [^6^A + OH × 2]^+^ (C_10_H_8_O_2_ + OH × 2), *m/z* 221 [^6^A + OCH_3_ × 2]^+^ (C_10_H_8_O_2_ + OCH_3_ × 2), and *m/z* 210 [^6^A + Cl + OH]^+^ (C_10_H_8_O_2_ + Cl + OH). Moreover, the [^1,3^A]^+^ produced by the retro-Diels–Alder (RDA) fragmentation pathway of the C-ring were also characteristic for FTPECs. Fifty-two FTPECs in total were thereby detected according to their retention time (*t*_R_) as well as the characteristic fragment ions (Table S1).

Compounds **39**, **58**, and **121** were studied as representatives of FTPECs. The HRESIMS analysis of **39** revealed the presence of a chlorine atom. Its molecular formula, C_17_H_13_ClO_3_, was obtained from the HRESIMS *m/z* 301.0604 ([M + H]^+^). The fragment ion at *m/z* 210 was designated as [^6^A + OH + Cl]^+^, indicating the distributions of one hydroxy and a Cl-atom substitutes at A-ring. Therefore, **39** was putatively identified as 8-chloro-6-hydroxy-2-(2-phenylethyl)chromone [28]. Compound **58** (C_18_H_16_O_5_) had the protonated molecular [M + H]^+^ ions at *m/z* 313.1046, with 11 indices of hydrogen deficiency. It generated the tandem fragment ion at *m/z*192 [^6^A + OH × 2]^+^ and *m/z* 121 [^6^B + OCH_3_]^+^, revealing two hydroxy groups occurred in A-ring, one methoxy group in B-ring; hence, compound **58** was plausibly identified as 6,7-dihydroxy-2-[2-(4′-methoxyphenyl)ethyl]chromone [29]. Compound **121** shared the protonated molecular ion at *m/z* 299.0921 [M + H]^+^, yet displayed a distinct fragment ion at *m/z* 208 [^6^A + OH × 3]^+^ and *m/z* 169 [^1,3^A + OH × 3]^+^. Thus, **121** was putatively identified as trihydroxy-2-(2-phenylethyl)chromone, which has not been detected in previous reports, so it should be a new compound. In addition, compounds **48**, **53**, **87**, **89**, **51**, **55,** and **88** were classified as PEC glycoside analogues due to the neutral cleavage of 162 Da in their HR-MS/MS spectra. Compounds **48**, **53**, **87**, and **89** exhibited comparable mass spectral patterns, featuring the protonated molecular ion at *m/z* 429 ([M + H]^+^) and the product ions of *m/z* 267 [M + H-162]^+^, *m/z* 176 [^6^A + OH]^+^ and *m/z* 137 [^1,3^A + OH]^+^; hence, the structure of **48**, **53**, **87**, and **89**, were tentatively elucidated as 2-(2-phenylethyl)chromone-8-*O*-*β*-D-glucopyranoside or isomer [30], which was previously purified from *Imperata cylindrica*. The molecular weight of **51**, **55**, and **88** was 458 Da, the same as 2-[2-(4-glucosyloxy-3-methoxyphenyl)ethyl]chromone, which was acquired from Chinese agarwood previously [31]. Nevertheless, they displayed distinct product ion profiles. The fragment ion at *m/z* 297 [M + H-162]^+^, *m/z* 206 [^6^A + OH + OCH_3_]^+^ and *m/z* 167 [^1,3^A + OH + OCH_3_]^+^, indicated that one hydroxy and one methoxy occurred in A-ring. Thus, compounds **51**, **55**, and **88** were assigned as methoxy 2-(2-phenylethyl)chromone-8-O-β-D-glucopyranoside or isomer, which has not been reported.

### Identification of THPECs (II) in agarwood

The representative mass cracking rules of the THPECs are the successive neutral losses of two H_2_O molecules (18 Da + 18 Da) and then two CO molecules (28 Da + 28 Da) (Fig. S2C, Supporting Information). In addition, the substituted benzyl ions such as *m/z* 91, *m/z* 107, *m/z* 121, and *m/z* 137 are also characteristic ions of THPECs. On the basis of the above MS fragmentation rules, forty-six THPECs were detected from Chinese agarwood.

Compounds **12**, **16**, **26**, **29**, and **57** were classified into THPECs on the basis of their characteristic mass cracking rules. Compounds **16** and **26** exhibited comparable mass spectral patterns, featuring the neutral cleavages of MeOH and H_2_O; hence, the structure of compounds **16** and **26** were tentatively assigned to methylated THPECs. The molecular formula of compound **16** was predicted as C_18_H_20_O_6_, featuring the fragment ion at *m/z* 301 [M + H–MeOH]^+^, *m/z* 283 [M + H–MeOH–H_2_O]^+^, and *m/z* 255 [M + H–MeOH–H_2_O–CO]^+^. Consequently, **16** were identified as 5,6,7-trihydroxy-8-methoxy-5,6,7,8-tetrahydro-2-(2-phenylethyl) chromone [32]. The molecular weight of **26** was 346 Da, 15 Da (CH_3_) more than **16**. Compound **26** yielded the fragment ions at 315 [M + H–MeOH]^+^, *m/z* 283 [M + H–2MeOH]^+^, *m/z* 255 [M + H–2MeOH–CO]^+^, and *m/z* 227 [M + H–2MeOH–2CO]^+^, indicating that A-ring had two methoxy groups, and it was tentatively determined as 6,7-dihydroxy-5,8-dimethoxy-5,6,7,8-tetrahydro-2-(2-phenylethyl)chromone, which was previously undescribed. Compound **29 (**C_17_H_17_ClO_5_**)** was assigned to chlorinated THPECs, which supported by the approximate 3:1 intensity ratio of the ions [M + H]^+^/[M + 2H]^+^ and the neutral cleavages of HCl. The fragment ions at *m/z* 319 [M + H–H_2_O]^+^, *m/z* 301 [M + H–2H_2_O]^+^, 283 [M + H–H_2_O–HCl]^+^, *m/z* 265 [M + H–2H_2_O–HCl]^+^, and *m/z* 255 [M + H–H_2_O–HCl–CO]^+^,were observed for **29**; hence, compound **29** was identified as 8-chloro-5,6,7-trihydroxy-2-(2-phenylethyl)−5,6,7,8-tetrahydrochromone isomers [33]. Compounds **12** and **57** have the same molecular weight of 284 Da, with the molecular formula C_17_H_18_O_5_, and produced the same fragment ion at *m/z* 267 [M + H–H_2_O]^+^ and *m/z* 239 [M + H–H_2_O–CO]^+^, indicating that they contained one double bond and two hydroxy group in A ring. Moreover, [M + H–H_2_O–CO–^6^B]^+^ at *m/z* 148 for **12** and [M + H–H_2_O–^6^B]^+^ at *m/z* 176 for **57** inferred that the B ring is presumably unsubstituted. Therefore, compounds **12** and **57** was putatively identified as 5,6-dihydroxy-2-phenethyl-5,6-dihydro-4H-chromen-4-one isomers, marking their initial discovery in the extracts of agarwood.

### Identification of DEPECs (III) in agarwood

In general, successive neutral cleavage of two molecules of CO, along with the substituted benzyl ions was the diagnostic properties of the DEPECs (Fig. S2D, Supporting Information). As a result, compounds **4**, **126**, and **171** were tentatively yielded as DEPECs, and the speculative details are summarized in Table S1. Compound **49** had a [M + H]^+^ ion at *m/z* 299 in accord with a C_17_H_14_O_5_ formula, and afforded the fragment ion at *m/z* 253 [M + H–H_2_O–CO]^+^, *m/z* 162 [M + H–H_2_O–CO–^6^B]^+^, *m/z* 123 [M + H–H_2_O–2CO–^5^B]^+^, and *m/z* 91 [^6^B]^+^. Hence, compound **49** was assembled as 5,6:7,8-diepoxy-2-[2-(7′-hydroxyphenyl)ethyl]−5,6,7,8-tetrahydrochromone, which is a potential new compounds.

### Identification of EPECs (IV) in agarwood

The neutral elimination of one H_2_O (18 Da) and two CO (28 Da + 28 Da) were the characteristic ions of EPECs. Consequently, nine EPECs were assigned. Compounds **3**, **34**, **83**, **123**, and **190** were preliminarily detected based one the presence of signals at *m/z* 283 [M + H–H_2_O]^+^, *m/z* 255 [M + H–H_2_O–CO]^+^, and *m/z* 227 [M + H–H_2_O–2CO]^+^, and the detailed information are elucidated in Table S1. Compounds **10, 52**, **122**, and **54** exhibited comparable mass spectral patterns, featuring the neutral cleavages of H_2_O and 2CO. In the MS spectrum, the [^6^B + OCH_3_]^+^ fragment ion at *m/z* 121 was observed for compounds **10**, **52**, and **122**, while the [^6^B + OH]^+^ fragment ion at *m/z* 107 was observed for compound **54**; hence, the structure of compounds **10, 52**, and **122** were tentatively elucidated as rel-(1a*R*,2*R*,3*R*,7b*S*)−1a,2,3,7b-tetrahydro-2,3-dihydroxy-5-[2-(4-metoxyphenyl)ethyl]- 7*H*-oxireno[f] [1]benzopyran-7-one [34], while **54** detected as 5,6-epoxy-7,8-dihydroxy-2-[2-(4′-hydroxyphenyl)ethyl]−5,6,7,8-tetrahydrochromone.

### Identification of PEC dimers in agarwood

As far as we know, dimeric PECs are a unique group of polyphenols exclusively found in *Aquilaria* species (Thymelaeaceae). These compounds consist of two PEC units linked through carbon–carbon and/or carbon–oxygen bonds. According to the connection form of substructure, PEC dimers can be divided into six types (Fig. S3, Supporting Information): two FTPECs monomers connected via carbon–carbon bonds (A), one FTPECs monomers and one THPECs monomers connected via carbon–oxygen bonds (B), one FTPECs monomers and one THPECs monomers connected via dioxan (C), one FTPECs monomers and one THPECs monomers connected via 6,7-dihydro-5H-1,4-dioxepine moiety (D), one FTPECs monomers and one EPECs monomers or two EPECs connected via carbon–oxygen bonds (E), one FTPECs monomers and one THPECs monomers connected via 3,4-dihydro-2H-pyran ring (F). Eighteen authentic PEC dimers were gathered and analyzed by IT-TOF–MS to acquire the mass fragmentation rules as well as UV absorption features of this essential chemical component in Chinese agarwood. Typical MS^n^ data for the eighteen PEC dimers are illustrated in Table 1.

**Table 1:** UV Data, molecular formulae, and primary MS/MS spectral information of eighteen 2-(2-phenylethyl)chromone dimers

Compounds	Type^a^	UV data *λ*_max_ (nm)	Molecular formulae	[M + H]^+^		Error (ppm)	MS/MS^b^
				Measured	Calcd		
Crassin A	A	206, 231, 328	C_36_H_30_O_8_	591.2104	591.2103	0.17	573, 471*, 453, 431, 351, 297, 269
Aquisinenone N	B	205, 227, 278, 314	C_37_H_36_O_12_	673.2294	673.2280	2.08	655*, 637, 609, 475, 343, 313, 285
Aquisinenone G	D	206, 234, 312	C_34_H_28_O_8_	565.1859	565.1857	0.35	547, 529, 474, 410, 283*, 255, 192, 153
4′-methoxy-aquisinenone G	D	205, 225, 314	C_35_H_30_O_9_	595.1961	595.1963	–0.34	577, 559, 474, 313, 283*, 255, 192
4′,4‴-dimethoxyaquisinenone K	E	203, 251, 325	C_36_H_34_O_11_	643.2175	643.2174	0.16	625, 607, 331, 313*, 285, 121
aquisinenone I	E	209, 225, 321	C_34_H_28_O_7_	549.1886	549.1908	–4.01	531, 503, 283, 267*, 176, 137, 91
7″-methoxyaquisinenone I	E	207, 225, 320	C_35_H_30_O_8_	579.2015	579.2013	0.35	561, 533, 297*, 283, 206, 191, 167, 121
4′,7″-dimethoxyaquisinenone I	E	209, 228, 312	C_36_H_32_O_9_	609.2100	609.2119	–3.12	591, 563, 327, 313, 297*, 206, 191, 167, 121
Aquisinenone A	F	207, 235, 331	C_34_H_28_O_7_	549.1907	549.1908	–0.18	531, 485, 458*, 423, 357, 329, 266,
4′-methoxyaquisinenone A	F	206, 230, 329	C_35_H_30_O_8_	579.2018	579.2013	0.86	561, 515, 487, 458*, 423, 357, 329, 303, 266
Aquisinenone C	F	207, 250, 350	C_34_H_28_O_8_	565.1872	565.1857	2.65	547, 529*, 501, 474, 373, 283, 192
Aquisinenone B	F	206, 237, 306	C_34_H_28_O_7_	549.1900	549.1908	–1.46	531*, 513, 485, 458, 423, 357, 329, 279, 266, 253, 225
6′′-hydroxyaquisinenone B	F	206. 245, 333	C_34_H_28_O_8_	565.1882	565.1857	4.42	547, 529, 501, 474*, 373, 345, 283, 192
6′′-hydroxy-4′,4′′′-dimethoxyaquisinenone B	F	224, 243, 333	C_36_H_32_O_10_	625.2073	625.2068	0.80	607, 589, 579*, 561, 533, 375, 401, 347, 311, 297
aquisinenone D	F	207, 225, 324	C_37_H_34_O_10_	639.2197	639.2225	–4.38	603, 489, 449, 431, 325*, 310, 293, 265, 151, 121
4′-demethoxyaquisinenone D	F	207, 225, 324	C_36_H_32_O_9_	609.2116	609.2119	–0.49	573, 459, 419, 401, 325*, 310, 293, 265, 151
Aquisinenone E	F	205, 224, 321	C_36_H_32_O_9_	609.2134	609.2119	2.46	591, 573, 459, 429, 411, 401, 387, 295
Aquisinenone F	F	207, 224, 322	C_35_H_30_O_8_	579.2034	579.2013	3.63	561*, 543, 429, 447, 411, 387, 371, 295

^a^A-type: two FTPEC monomers are connected by a single C–C bond, B-type: one FTPEC monomer and one THPEC monomer are connected by a C–O–C bond, C-type: one FTPEC monomer and one THPEC monomer are connected by two C–O–C bonds forming a 2,3-dihydro-1,4-dioxine ring, D-type: one FTPEC monomer and one THPEC monomer are connected by two C–O–C bonds forming a 6,7-dihydro-5*H*−1,4-dioxepine ring, E-type: one FTPEC/EPEC monomer and one EPEC monomer are connected by a C–O–C bond, F-type: one FTPEC monomer and one THPEC monomer are connected by a C–C bond and a C–O–C bond forming a 3,4-dihydro-2*H*-pyran ring. ^b^Fragment ions marked with *were the base peak of MS^2^

### Mass fragmentation pathways

Eighteen reference compounds, comprising one A type, one B type, two D type, four E type, and ten F type PEC dimers, were utilized to characterize the mass fragmentation rules and UV absorption features of PEC dimers. On the basis of the fragmentation rules in previous reports [35], the cleavages of the C_7′_–C_8′_ bond and the breakage of the carbon–carbon bonds were demonstrated as the primary features of A type PEC dimers. Taken crassin A as an example compound, its molecular formula was deduced to be C_36_H_30_O_8_ due to the protonated molecular ion ([M + H]^+^) at *m/z* 591.2104. The tandem fragment ions observed, including *m/z* 573, 471, 453, 351, 297, 269, were in close alignment with the values reported for A type PEC dimers (Table 1).

As a typical B type PEC dimers, the protonated molecular ion of aquisinenone N was detected at *m/z* 673.2294 (Table 1), and the elementary fragments were collected at *m/z* 655 ([M + H–H_2_O]^+^), 637 ([M + H–2H_2_O]^+^), and 609 ([M + H–2H_2_O–CO]^+^), indicating the successive neutral loss of H_2_O (18 Da) and one CO (28 Da), while the product ion of *m/z* 343 ([M_unit B_ + H]^+^), *m/z* 313 ([M_unit A_ + H–2H_2_O]^+^), and *m/z* 285([M_unit A_ + H–2H_2_O–CO]^+^) corresponded to the ester bond cleavage (Table 1). The MS/MS pattern of B type dimer is consistent with previous studies on agarwood using LC–MS/MS analysis [35, 36].

The fragmentation pathway of C type PEC dimers has been previously summarized by Li et al. [35], and dissociation of H_2_O and CO molecule along with the cleavage of the ester bond between two monomers were revealed as the primary characteristics. The fragmentation pathway of this type closely resembles that of B type, with the notable exception that C type exhibits 21 indices of hydrogen deficiency.

**Fig. 2:**
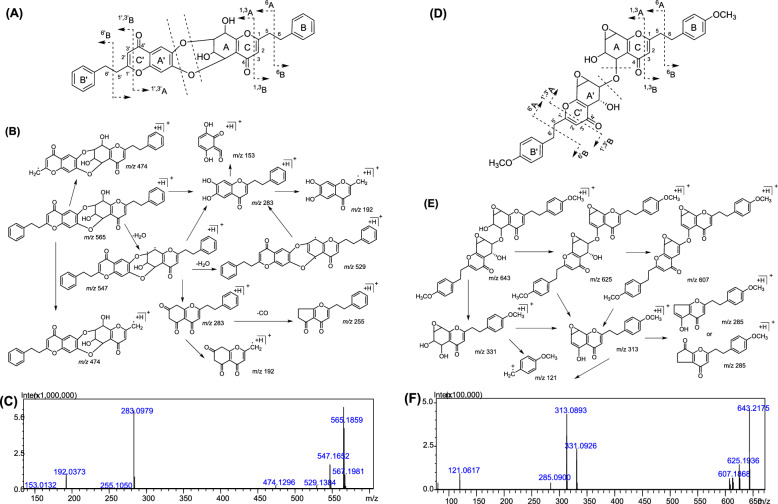
The ion nomenclature, proposed mass fragmentation pathways, and the MS/MS of chemical reference standard of aquisinenone G (representative D-type dimer, **A**, **B**, **C**) and 4′,4‴-dimethoxyaquisinenone K (representative E-type dimer, **D**, **E**, **F**)

For the first time, the fragmentation patterns of D type dimers were discussed using two purified standards (aquisinenone G and 4′-methoxy-aquisinenone G). Aquisinenone G serves as an exemplary compound to elucidate its distinct mass fragmentation behaviors. Its molecular formula, C_34_H_28_O_8_, corresponds to a mass of *m/z* 565.1859 and features 21 degrees of unsaturation. In the MS spectrum, the fragment ion at *m/z* 547([M + H–H_2_O]^+^), 529 ([M + H–2H_2_O]^+^), 474 ([M + H − ^6^B]^+^)/([M + H − ^6′^B]^+^), and the MS^2^ base peak ion *m/z* 283 stemming from the breakage of the ester bonds between two monomers, were observed for aquisinenone G. The monomer-derived fragment ions (*m/z* 283) further produced [M_unit A_ + H–CO]^+^ at *m/z* 255. In addition, the monomer-derived fragment ions also afforded the fragment ion at *m/z* 192 and *m/z* 153 resulting from the dissociation of the C_7′_–C_8′_ bond and the RDA dissociation (Fig. 2B).

Although E type dimer and B type dimer showed similar MS fragmentation pathways, the monomer-derived fragment ions of E type dimer were the base peak in MS^2^ (Table 1). 4′,4′′′-dimethoxyaquisinenone K and aquisinenone I and were studied as representatives of E type. 4′,4′′′-dimethoxyaquisinenone K (*m/z* 643.2175) continuously lost two molecules of H_2_O to produce fragment *m/z* 625 ([M + H − H_2_O]^+^) and *m/z* 607 [M + H − 2H_2_O]^+^, and it could also break the ester bonds between two monomers to produce the monomer-derived fragment ions at *m/z* 331. The monomer-derived fragment ions produced MS^2^ base peak ion at *m/z* 313[M_unit A_ + H − H_2_O]^+^, which further produced *m/z* 285 [M_unit A_ + H − H_2_O − CO]^+^ and *m/z* 121 [^6^B + OCH_3_]^+^ (Fig. 2E). The tandem mass spectrum of aquisinenone I showed two characteristic cracking ions of *m/z* 283 and *m/z* 267, corresponding to the breakage of the ester bonds between two monomers. The monomer-derived fragment ions *m/z* 267 further generated the prominent fragment ion at *m/z* 176, *m/z* 91, and *m/z* 153 resulting from the disaggregation of the C_7′_–C_8′_ bond and the RDA dissociation (Fig. S4, Supporting Information). In addition, its [M + H]^+^ (*m/z* 549) could also yield a fragment *m/z* 531 ([M + H − H_2_O]^+^), which further produces [M + H − 2H_2_O]^+^ at *m/z* 503.

**Fig. 3:**
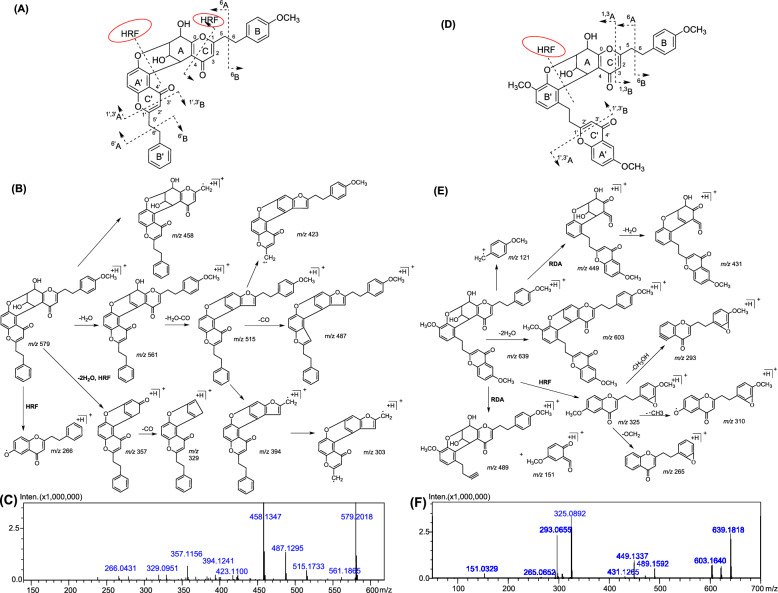
The ion nomenclature, proposed mass fragmentation pathways, and the MS/MS of chemical reference standard of 4′-methoxyaquisinenone A (representative F-type dimer connected via A and A′ ring, **A**, **B**, **C**) and aquisinenone G (representative F-type dimer connected via A and B′ ring, **D**, **E**, **F**)

According to the position of linkages, F type dimers are divided into two categories: two monomers connected via A and A′ ring and two monomers connected via A and B′ ring group, and their characteristic fragments and primary cleavage pathways were sketched in Fig. 3. For F-type dimer connected by A and A′ ring, the heterocyclic ring fission (HRF)-initiated synergetic cleavage of the linkages of units A and B and bonds 1/4 to produce the monomer-derived fragment ions and ^1,4^A, and the dissociation of the C_7′_–C_8′_ bond to generate ^6^A and ^6′^A were considered a diagnostic feature characteristic of this class of dimers. 4′-methoxyaquisinenone A (C_35_H_30_O_8_) served as an exemplary compound to demonstrate their fragmentation characteristics (Fig. 3B). In the MS spectrum of compound 4′-methoxyaquisinenone A, we observed *m/z* 266 produced by the HRF-initiated synergetic cleavage of the linkages of units A and B, *m/z* 458 [M + H–^6^B]^+^ produced by the disaggregation of the C_7′_–C_8′_ bond, and *m/z* 357 produced by the neutral dissociation of two H_2_O molecules and the HRF-initiated collaborative cracking of bonds 1/4, which further produced [^1,4^A − 2H_2_O − CO]^+^ at *m/z* 329. In the MS spectrum, the fragment ion at *m/z* 561([M + H–H_2_O]^+^), 515 ([M + H–2H_2_O − CO]^+^), and 487 ([M + H–2H_2_O − 2CO]^+^) were also observed for 4′-methoxyaquisinenone A. [M + H–2H_2_O − CO]^+^ (*m/z* 515) could also break the C_7′_–C_8′_ bond to yield ([M + H–2H_2_O–CO–^6′^B]^+^) (*m/z* 423) and ([M + H–2H_2_O–CO–^6^B]^+^) (*m/z* 394), which further produced [M + H–2H_2_O–CO–^6^B–^6′^B]^+^ at *m/z* 303. The HRF-initiated synergetic cleavage of the linkages of units A and B, RDA cracking of C and C′ ring to produce^1,3^A and ^1′,3′^B, and the dissociation of the C_7′_–C_8′_ bond to generate ^6^A and ^6′^A were considered a diagnostic feature characteristic of F-type dimer connected by A and B′ ring. Using aquisinenone D as an illustrative case, its predicted molecular formula was C_37_H_34_O_10_. Upon HRF-mediated synergistic cleavage of the bonds connecting units A and B, a fragment ion at *m/z* 325 ([M_unit B_ + H]^+^) was generated. This cleavage cascade further yielded [M_unit B_ + H–·CH_3_]^+^ at *m/z* 310, [M_unit B_ + H–CH_3_OH]^+^ at *m/z* 293, and [M_unit B_ + H–2OCH_2_]^+^ at *m/z* 265. The [M + H]^+^ (*m/z* 639.2197) could also underwent RDA dissociation of C′-ring and C-ring to derive 489 [^1′,3′^B]^+^, *m/z* 151 [^1′,3′^A]^+^, and *m/z* 449 [^1′,3′^A]^+^, which further produced [^1′,3′^A–H_2_O]^+^ at *m/z* 431. In addition, the typical [M + H–2H_2_O]^+^ (*m/z* 603) and [^6^B + OCH_3_]^+^ (*m/z* 121) ions were also detected for aquisinenone D (Fig. 3E). So far as we know this is the first time to systematically discuss the F-type dimer cleavage pathway using purified standards.

### A type PEC dimers

In this study, only four A type PEC dimers were found and identified. Compound **164** had a [M + H]^+^ ion at *m/z* 531 in accord with a C_34_H_26_O_6_ formula, and afforded the fragment ion at *m/z* 513 [M + H–H_2_O]^+^, *m/z* 453 [M + H–H_2_O–^7^B]^+^, *m/z* 440 [M + H–H_2_O–^6^B]^+^. Additionally, compound **164** exhibited a feature fragment at *m/z* 267, which was produced by the breakage of the carbon–carbon bonds. Hence, compound **164** was tentatively determined to be 6,8′-dihydroxy-2,2′-diphenethyl-4*H*,4′*H*-[5,5′-bichromene]−4,41-dione (AH_11_) or isomer [37]. The molecular ion of compound **82**, protonated at *m/z* 547 [M + H]^+^, and its fragment ions, notably *m/z* 529 [M + H–H_2_O]^+^ and *m/z* 456 [M + H–H_2_O–^6^B]^+^, each exhibited 16 Da greater than **164**. Furthermore, the compound produced an additional fragment at *m/z* 282, stem from from the cleavage of carbon–carbon bonds between its constituent monomers. Thus, compound **82** was preliminarily assigned as hydroxy-AH_11_. Analogously, compounds **71** and **167** was tentatively classified as dehydroxy-dimethoxy-AH_11_ and methoxy-AH_11_, respectively. Detailed information is provided in Table S1.

### B type PEC dimers

In total, twenty-three B type PEC dimers were determined utilizing both reference compounds and literature data. Compounds **175**, **124**, and **160** were studied as representatives of FTPECs. The molecular weight of both **175** was 596 Da, 30 Da (OCH_3_) more than AH_13_ [35], and the common fragments at *m/z* 565 ([M + H–MeOH]^+^), 547 ([M + H–MeOH–H_2_O]^+^), and 283 ([M_unit A_ + H–MeOH–H_2_O]^+^) were observed for **175**. Besides that, compound **175** also yielded the monomer-derived feature fragments at *m/z* 297 ([M_unit A_ + H − H_2_O]^+^), 206 ([M_unit A_ + H − H_2_O − ^6^B]^+^), and 91 ([^6^B]^+^); therefore, compound **175** was tentatively identified as 5-methoxy-AH_13_. Compound **124** had a protonated ion at *m/z* 619.2215 ([M + H]^+^) and conform to the formula of C_34_H_34_O_11_, which had 18 indices of hydrogen deficiency. Compound **127** yielded unique fragments at *m/z* 601 ([M + H–H_2_O]^+^), 301 ([M_unit A_ + H–H_2_O]^+^), 255 ([M_unit A_ + H–H_2_O–CO]^+^), and 227 ([M_unit A_ + H–H_2_O–2CO]^+^). Therefore, compound **124** was assembled as a dimer formed by the connection of two THPECs through carbon–oxygen bonds. The molecular weight of compound **160** was 618 Da, which is the same as **124**. However, the molecular formula of compound **160** was C_36_H_36_O_10_, with 19 indices of hydrogen deficiency, exhibited feature fragments at *m/z* 601 [M + H–H_2_O]^+^, 319 ([M_unit A_ + H]^+^), 255 ([M_unit A_ + H–2H_2_O]^+^), and 227 ([M_unit A_ + H–2H_2_O–2CO]^+^). Thus, compound **160** was also assigned as B type dimer comprising one THPEC and one EPEC, where the EPEC unit contains two methoxy groups. It is worth mentioning that compounds **124** and **160** were reported for the first time.

### C type PEC dimers

Compounds **112** and **186** had same protonated ion at *m/z* 565 ([M + H]^+^) and conform to the formula of C_34_H_28_O_8_, which had 21 indices of hydrogen deficiency. They produced primary fragment ions at *m/z* 547 ([M + H–H_2_O]^+^), 529 ([M + H–2H_2_O]^+^), 283 ([M_unit A_ + H–2H_2_O]^+^), and 206 ([M_unit A_ + H − 2H_2_O − ^6^B]^+^), conclusively confirming the presence of AH_21_ due to the identical mass fragmentation pattern reported in the literature [35]. According to a series of distinctive mass spectrometry data, a pair of isomers, **81** and **103** (C_35_H_30_O_9_) were preliminary identified as methoxy-AH_21_, and compound **70** was proposed as dimethoxy-AH_21_. Detailed information can be found in Table S1.

### D type PEC dimers

D type PEC dimers had 21 degrees of unsaturation and the monomer-derived fragment ions were the base peak in the MS^2^ spectrum. Taking **100** as an exemplary compound, the same molecular formula, C_34_H_28_O_8_, was predicted for both compounds **100** and aquisinenone G, and they exhibited similar fragmentation pathways, including the fragment ion at *m/z* 547 ([M + H − H_2_O]^+^) and *m/z* 529 ([M + H − 2H_2_O]^+^) and the MS^2^ base peak ion at *m/z* 283 corresponding to the damage of the carbon–carbon bonds between two monomers. The monomer-derived fragment ion (*m/z* 283) could also neutral loss of one CO molecule to produce [M_unit A_ + H − CO]^+^ (*m/z* 255) ion. Therefore, compound **100** was tentatively identified as aquisinenone G isomers [22]. Analogously, through the comprehensive analysis of MS/MS spectra, compounds **153**, **165**, and **185** were plausibly characterized as 4′-methoxyaquisinenone G isomers. Detailed information was elaborated in Table S1.

### E type PEC dimers

The structure of E type PEC dimers corresponds to the B-type, with the exception that the monomer-derived fragment ions were the base peak in the MS^2^ spectrum of the E-type dimer. Compound **141** exhibited the molecular formula C_34_H_30_O_9_ in the light of the protonated molecular ion at *m/z* 583.1960 [M + H]^+^, suggesting 20 indices of hydrogen deficiency. A series of characteristic ions such as *m/z* 565 [M + H–H_2_O]^+^, *m/z* 547 [M + H–2H_2_O]^+^and *m/z* 283 produced by breaking the ester bonds between two monomers were detected in the mass spectra of **141**. Among them, fragment ion *m/z* 283 is the base peak in MS^2^, which further produced *m/z* 255 [M_unit A_ + H − H_2_O − CO]^+^, *m/z* 255 [M_unit A_ + H − H_2_O − ^6^B]^+^, and *m/z* 91 [^6^B]^+^, and its cleavage pathway is the same as that of aquisinenone K. Therefore, compound **141** was identified as isomers of aquisinenone K [21]. Compound **161** (C_37_H_34_O_10_) had one more methoxy than aquisinenone J (C_36_H_32_O_9_) [21], and produced the *m/z* 621 [M + H–H_2_O]^+^ and *m/z* 593 [M + H–H_2_O–CO]^+^ ions. The mass of these ions increased by 30 Da, relative to the corresponding fragment ions identified in aquisinenone J. The monomer-derived fragment ions at *m/z* 357 [M_unit B_ + H]^+^, *m/z* 221 [^6^A + 2OCH_3_]^+^, and 137 [^6^B + OCH_3_ + OH]^+^ indicated an additional methoxy group on the A-ring. Therefore, compound **161** was tentatively classified as 6′′-methoxy-aquisinenone J.

### F type PEC dimers

F type PEC dimers could be readily distinguished based on their distinctive MS dissociation behaviors in positive mode. The molecular weight of **101** was 594 Da, 46 Da (OH + OCH_3_) more than aquisinenone A. Compound **101** generated feature fragments at *m/z* 577 [M + H–H_2_O]^+^, 559 [M + H–2H_2_O]^+^, 489 ([M + H–^6^B]^+^), 368 ([M + H–^6^B–^6′^B]^+^), and *m/z* 297 [M_unit B_ + H]^+^ ions, indicating B′-ring possessed a methoxy group substitution and B-ring possessed a hydroxyl group substitution. Thusly, compound **101** was tentatively assigned as 4′-hydroxy-4′′′-methoxy-aquisinenone A. In the mass spectra, a series of characteristic ions such as *m/z* 561 [M + H–H_2_O]^+^, 543 [M + H–2H_2_O]^+^, 488 ([M + H–^6^B]^+^), 453 ([M + H–^6^B–^6′^B]^+^), and *m/z* 359 produced by the successive neutral loss of two H_2_O molecules, one CO molecule and the HRF-initiated synergetic cleavage of bonds 1/4 were detected in the mass spectra of compound **187**. Notably, all these ions exhibited masses 30 units higher than those of aquisinenone B, indicating that compound **187** is likely an *O*-methyl derivative of aquisinenone B. Accordingly, compound **187** was tentatively elucidated as 6′′-methoxy-aquisinenone B.

### Identification of trimers in agarwood

According to the formerly summarized mass fragmentation rules [35], the neutral cleavages of H_2_O and the cleavage of the ester bond between three monomers were revealed as the diagnostic behaviors for PEC trimers. In this study, only five trimers were found and identified. Compound **113** exhibited protonated molecular ion of [M + H]^+^ at *m/z* 883.2913, stemming from the molecular formula of C_51_H_46_O_14_. They derived fragment ions at *m/z* 865 ([M + H–H_2_O]^+^), 847 ([M + H–2H_2_O]^+^), 829 ([M + H–3H_2_O]^+^), 811 ([M + H–4H_2_O]^+^), 583 ([M + H–M_unit A_]^+^), 565 ([M + H–M_unit A_ − H_2_O]^+^), 547 ([M + H–M_unit A_ − 2H_2_O]^+^), and 519 ([M + H–M_unit A_ − 2H_2_O − CO]^+^). These ions were consistent with the mass fragmentation pattern recorded in the previous research [35], leading to the identification of the compound as tri-2-(2-phenylethyl)chromone. In a similar way, compounds **106**, **114**, **163**, and **168** were proposed as tri-2-(2-phenylethyl)chromone isomer, which has not been reported. Detailed information can be found in Table S1.

Above all, a total of 199 PECs were identified in Chinese agarwood through matching the information with reference compounds, comparing and analyzing the distinctive mass spectrometry data, and utilizing the proposed mass cracking rules for chemical assignment, including 56 FTPECs, 48 THPECs, six DEPECs, ten EPECs, 74 PEC dimers, and five PEC trimers. Notably, 42 of these compounds were identified or preliminary identified as new compounds, with detailed information provided in Table S1.

### Structural elucidation of the purified PEC dimers

**Fig. 4:**
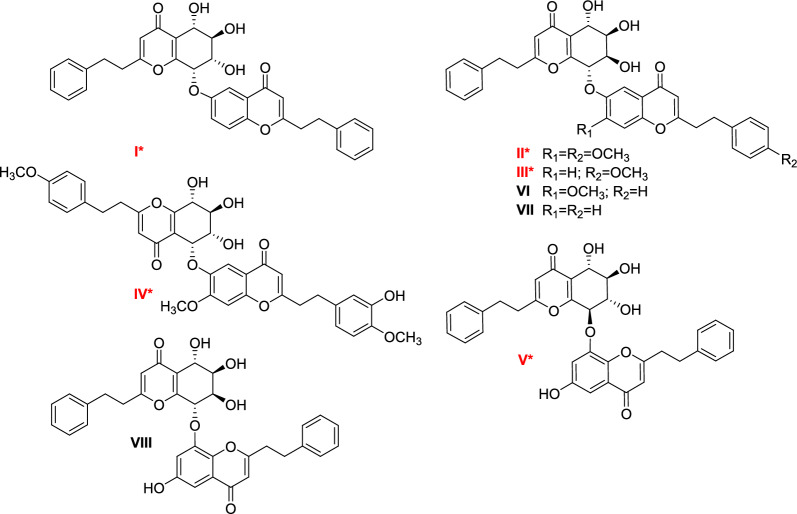
Chemical structures of compounds **I**–**VIII** from Chinese agarwood

To enhance structural annotation confidence, an HPLC–MS guided fractionation procedure was executed for targeted purification of PEC dimers. Subsequent purification, guided by LC–MS, resulted in the purification of eight new PEC dimers, comprising three pairs of scalemic mixtures (**IIa**/**IIb**, **IIIa**/**IIIb**, and **IVa**/**IVb**), and two optically pure PEC dimers (**I** and **V**) (Fig. 4). Their structures were determined through extensive spectroscopic analysis (HRMS, UV, IR, and 1D/2D NMR) and ECD data. Three known compounds were identified as AH_12_ (**VI**)[38], AH_13_ (**VII**)[38], and diaquilariachromone B (**VIII**) [39] by comparing their 1D NMR and MS information with published data. And more importantly, all the structures were able to verify structural annotation relied on LC–MS/MS.

Aquisinenone P (**I**), isolated as a white amorphous powder with [*α*]25 D –72 (*c* 0.1, MeOH), was characterized by its molecular formula C_34_H_30_O_8_, which was inferred from comprehensive ^13^C NMR spectroscopic data and confirmed by the HRESIMS positive ion at *m/z* 567.2023 ([M + H]^+^, calcd for C_34_H_31_O_8_, 567.2013), requiring 20 indices of hydrogen deficiency. The existence of hydroxy (3344 cm^−1^) and *α*,*β*-unsaturated carbonyl (1656 cm^−1^) functionalities was provided by the IR spectrum. The ^1^H NMR data of **I** (Table 2) contains the signals of a pair of aromatic protons [*δ*_H_ 5.98(1H, s), 5.94 (1H, s)], two sets of phenylethyl groups [*δ*_H_ 7.11 (4H, m), 7.03 (3H, m), 6.97 (1H, m), 6.84 (2H, d, *J* = 7.2 Hz); 2.54 (2H, m), 2.56 (2H, m), 2.75 (2H, t, *J* = 8.0 Hz), 2.85 (2H, t, *J* = 8.0 Hz)], an ABX pattern benzene ring [*δ*_H_ 7.69 (1H, d, *J* = 2.0 Hz), 7.15 (1H, dd, *J* = 8.8, 2.0 Hz),7.19 (1H, d, *J* = 8.8 Hz)], and four consecutive methines [*δ*_H_ 4.83 (1H, d, *J* = 5.6 Hz), 4.08 (1H, m), 4.32 (1H, m), 5.19 (1H, m)]. The ^13^C NMR and HSQC spectrum displayed 34 carbon resonances comprising two carbonyl (*δ*_C_ 180.8, 178.4), two sets of phenylethyl group, and four oxygenated *sp*^*3*^ tertiary (*δ*_C_ 76.0, 71.9, 69.9, 67.5). These data suggested that compound **I** was a B type PEC dimer, comprising a THPEC and a FTPEC [40]. The ^1^H and ^13^C NMR data (Table 2) were nearly superimposable on those of (5*R*,6*R*,7*R*,8*S*)−2-(2-phenylethyl)−5,6,7-trihydroxy-5,6,7,8-tetrahydro-8-[2(2-phenylethyl)chromonyl-6-oxy]chromone, which was previously isolated from artificial agarwood [40]. Further 2D NMR experiments (Fig. S5, Supporting Information) facilitated the definitive determination of the planar structure of **I**, which matched that of the aforementioned compound. In the NOESY spectrum, the NOE correlations of H-5/H-7, H-7/H-8, 5′′/H-7, and 5′′/H-8 indicated that H-5, H-7 and H-8 were cofacial (Fig. S6, Supporting Information). Moreover, the relatively large coupling constants of H-5/H-6 revealed their *trans* relationships [41]. Subsequently, a notable consistency was observed between the experimental ECD spectrum and that calculated for (5S,6R,7S,8S)-**I** (Fig. S7, Supporting Information). This correspondence confirmed the absolute configuration of compound **I**. Therefore, aquisinenone P (**I**) was defined as shown.

**Table 2:** ^1^H and ^13^C NMR information and signal assignments of compounds *I*–*V* (*δ* in ppm, *J* in Hz)

No	I^a^		II^b^		III^b^		IV^b^		V^b^	
	δ_*H*_	δ_*C*_	δ_*H*_	δ_*C*_	δ_*H*_	δ_*C*_	δ_*H*_	δ_*C*_	δ_*H*_	δ_*C*_
2		169.6		171.0		171.7		171.0		170.6
3	5.94, s	113.7	6.14, s	114.5	6.15, s	114.5	6.11, s	114.1	6.18, s	114.4
4		180.8		181.6		181.5		181.1		182.0
5	4.83 d (5.6)	67.5	4.82 d (4.0)	66.6	4.80 d (3.2)	66.3	5.41, d (3.0)	74.5	4.80 d (6.8)	70.3
6	4.08, m	71.9	4.13, t(2.8)	74.3	4.11, br s	74.5	4.24, t(3.0)	70.6	3.86, t (8.4)	74.9
7	4.32, m	69.9	4.43 d (7.2)	71.0	4.38 d (8.0)	70.8	4.15, dd (8.4, 1.8)	72.5	4.08, t (8.4)	73.6
8	5.19, m	76.0	5.46, d (7.2)	78.6	5.45, d (7.2)	78.2	4.65, dd (8.4, 1.8)	69.8	5.50 d (4.8)	79.5
9		158.5		162.5		162.7		166.9		160.5
10		122.6		122.9		122.7		119.2		122.5
1'		139.8		140.9		140.9		133.2		140.9
2'	6.84, d (7.2)	128.3	6.96,m	129.2	6.93, m	129.2	7.15, d (8.4)	130.4	6.94, d (7.2)	129.2
3'	7.10 m	128.8	7.14,m	129.5	7.15, m	129.6	6.84, d (8.4)	115.0	7.16 m	129.5
4'	7.03 m	126.7	7.26,m	127.4	7.23, m	127.4		159.8	7.11 m	127.4
5'	7.10 m	128.8	7.22,m	129.5	7.12, m	129.6	6.84, d (8.4)	115.0	7.16 m	129.5
6'	6.84, d (7.2)	128.3	7.18,m	129.2	7.22, m	129.2	7.15, d (8.4)	130.4	6.94, d (7.2)	129.2
7'	2.56, m	32.7	2.68, m	33.2	2.65, m	34.0	2.99, m	32.9	2.73, m;	33.5
8'	2.54, m	35.5	2.62, m	36.3	2.61, m	36.2	2.94, m	36.6	2.60, m	36.4
2"		169.1		170.9		170.9		171.4		171.2
3"	5.98, s	109.6	6.10, s	110.1	6.14, s	110.1	6.09, s	110.6	6.10, s	109.7
4"		178.4		179.7		180.2		179.8		180.4
5"	7.69, d (2.0)	108.2	7.92, s	110.7	7.89, s	110.5	7.81, s	110.1	7.05, m	102.2
6"		155.5		148.9		158.6		147.8		157.6
7"	7.15, dd (8.8, 2.0)	124.9		157.5	7.61, d (4.0)	126.2		157.9	7.22, m	111.5
8"	7.19, d (8.8)	119.8	7.22, s	101.6	7.59, d (4.0)	120.8	7.12, s	101.5		150.6
9"		152.0		155.0		153.4		155.1		142.6
10"		124.2		117.4		125.0		117.4		126.2
1'"		139.5		133.2		133.1		134.2		141.3
2'"	7.02 m	128.7	6.87 d (8.4)	130.2	6.83 d (8.0)	130.1	6.68, d (1.8)	116.5	7.14 m	129.4
3'"	7.11 m	128.4	6.71 d (8.4)	114.5	6.69 d (8.0)	114.9		147.7	7.19 m	129.5
4'"	6.97 m	126.7		159.8		159.8		147.6	7.11 m	127.4
5'"	7.11 m	128.4	6.79 d (8.0)	114.9	6.77 d (7.6)	115.0	6.80 d (8.4)	112.9	7.19 m	129.5
6'"	7.02 m	128.7	7.12 d (8.0)	130.4	7.11 d (7.6)	130.4	6.63, dd (8.4, 1.8)	120.6	7.14 m	129.4
7'"	2.85, t (8.0)	33.1	3.09, m	33.4	3.06, m	33.2	2.96, m	33.5	3.02, m	33.9
8'"	2.75, t(8.0)	36.2	3.00, m	37.2	3.00, m	37.3	2.95, m	37.2	2.98, m	36.8
4'-OCH_3_							3.75, s	56.9		
7"-OCH_3_			4.00, s	57.1			3.93, s	55.6		
4"'-OCH_3_			3.72, s	55.6	3.71, s	55.6	3.80, s	56.4		

Compound **I** measured at 600 MHz for ^1^H NMR, 150 MHz for ^13^C NMR; others measured at 400 MHz for ^1^H NMR, 100 MHz for ^13^C NMR

^a^Measured in CDCl_3_; ^b^Measured in methanol-*d*_4_

Aquisinenone Q (**II)** was separated as white amorphous powder. The HR-ESI–MS produce a molecular formula of C_36_H_34_O_10_, by *m/z* 625.2093 [M – H]^–^, (calcd for C_36_H_33_O_10_, 625.2079), 60 mass units more than that of **I**. Analysis of the 1D NMR information (Table 2) of compound **II** evidenced a intimate structural similarity to **I**, with the exception of two methoxy group [*δ*_H_ 4.00 (3H, s), 3.72 (3H, s); *δ*_C_ 57.1, 55.6] in compound **II**. The deshielded resonances of C-7′′ (*δ*_C_ 157.5; Δ*δ*_C_ + 32.6) and C-4′′′ (*δ*_C_ 159.8; Δ*δ*_C_ + 33.1) suggested that the methoxy groups were located to C-7′′ and C-4′′′, which was substantiated by the HMBC correlations of 7′′-OCH_3_ to C-7′′, of 4′′′-OCH_3_ to C-4′′′. Additionally, NOE correlations observed between 7′′-OCH_3_/H-8′′, 4′′′-OCH_3_/H-3′′′, and 4′′′-OCH_3_/5′′′ further confirmed these assignments (Figs. S5 and S6, Supporting Information). The relative configuration of compound **II** was inferred to be identical to that of crassin N [41], based on their comparable ^3^*J*_H–H_ coupling constants for H-5/H-6/H-7/H-8. Notably, Compound II displayed a negligible optical rotation, coupled with the absence of a Cotton effect in its ECD spectrum, indicative of its racemic character. Subsequently, a chiral HPLC separation of compound **II** successfully yielded a pair of enantiomers, **IIa** and **IIb**. Through qualitative analysis of the ECD data (Fig. S7, Supporting Information), their absolute configurations at C-5, C-6, C-7 and C-8 were assigned as *R*,*S*,*S*,*R* and *S*,*R*,*R*,*S*, respectively.

7′′-Demethoxyaquisinenone Q (**III)** was established to hold the molecular formula C_35_H_32_O_9_, which showed a mass difference of 30 units compared to **II,** indicating that **III** is a demethoxy analogue of **II**. The 1D NMR spectroscopic information (Table 2) of **III** were closely resembled those of **II**, with the key differences lying in the substitution pattern on the phenyl ring and the absence of the 7′′-OCH_3_ resonance in **III**. Specifically, the 1,2,4,5-tetrasubstituted phenyl group in **II** is replaced by a 1,2,4-disubstituted phenyl group in **III**, which was proved by the HMBC spectrum (Fig. S5, Supporting Information). The NOESY data (Fig. S6, Supporting Information) confirmed that compound **III** has the same relative configuration as **II**. Chiral-phase HPLC analysis revealed that compound **III** is also a scalemic mixture. Enantiomers **IIIa** and **IIIb** were subsequently isolated, showing antipodal ECD (Fig. S7, Supporting Information) and specific rotations data. Further, compound **IIIa** was found to have the same absolute configuration as **IIa**, as evidenced by the matching signs of their specific rotations and the tendency of their ECD curves (Fig. S7, Supporting Information). Hence, **IIIa** and **IIIb** were unambiguously assigned as (5*R*,6*S*,7*S*,8*R*) and (5*S*,6*R*,7*R*,8*S*), respectively.

Aquisinenone R (**IV)**, a white amorphous powder, had the molecular formula of C_37_H_36_O_12_. The UV, IR, and NMR spectroscopic data of **IV** revealed that it contained structural units resembling THPEC and FTPEC units, akin to aquisinenone N previously obtained from Chinese agarwood (*Aquilaria sinensis*) [21]. The major difference lies in the connection between two units through a (5, O, 7″)-ether bond in **IV**. This deduction was supported by the HMBC correlations of H-5 to C-4/C-7/C-9/C-6′′ and H-5′′ to C-4′′/C-7′′/C-9′′ (Fig. S5, Supporting Information). The observed NOE correlations involving H-5/H-6, H-5/H-5′′, and H-6/H-5′′ (Fig. S6, Supporting Information), along with the *J*_H-5,6_ (3.0 Hz) indicated their *cis* relationship. Conversely, the *J*_H-7,8_ (8.4 Hz) suggested their *trans* relationship. The chiral HPLC separation of IV produced two enantiomers, **IVa** and **IVb**, and their absolute configurations were determined to be 5*S*,6*S*,7*R*,8*S* and 5*R*,6*R*,7*S*,8*R* on the basis of their ECD curves (Fig. S7, Supporting Information).

Aquisinenone S (**V),** [*α*]25 D –12 (*c* 0.1, MeOH), was gained a molecular formula C_34_H_30_O_9_, deriving from the ^13^C NMR and negative-ion HRESIMS data. The ^1^H and ^13^C NMR spectroscopic data obtained for compound **V** demonstrated a high degree of concordance with the corresponding data for crassin B [42]. Notable differences included a significant downfield shift of C-6″ (*δ*_C_ 157.6; Δ*δ*_C_ + 31.3) and the absence of resonances for the 4′′′-OCH_3_ in **V**. Taking the molecular formula into account, the significant downfield shift of C-6″ (*δ*_C_ 157.6; Δ*δ*_C_ + 31.3) displayed that a hydroxy group was located to C-6′′ in **V**. This assignment was verified by the HMBC correlations from H-5″ to C-4″/C-7″/C-9″ and from H-7″ to C-6″/C-8″/C-9″ (Fig. S5, Supporting Information). The structure including the absolute configuration of compound **V** was defined to match that of crassin B, supported by their fantastic NMR similarity, identical rotatory properties, and consistent ECD curves (Fig. S7, Supporting Information). Hence, the (5*R*,6*S*,7*R*,8*S*) absolute configuration of **V** was defined unambiguously.

## Discussion

TCM is highly complex, containing diverse compounds with varied properties and contents. Micro-components, though present in very low concentrations, often show strong bioactivity, making their isolation and characterization crucial for drug discovery. However, this process is challenging due to their scarcity, interference from other abundant ingredients, and structural diversity, which complicates detection using routine LC–MS/MS methods. Two-dimensional liquid chromatography, with its enhanced selectivity, peak capacity, and resolution, is an attractive tool for separating complex samples. In this study, we used a traditional C18 stationary phase to enrich micro-components from agarwood extract. These components were further separated on silica particles bonded with F5 phase and analyzed by UHPLC-DAD-IT-TOF–MS. Among diverse chromatographic columns, perfluorinated stationary phases offer excellent selectivity through a combination of dispersive interactions similar to those of C18 and C8 phases, dipole interactions from fluorine ligands, π-π interactions from phenyl ligands, as well as charge-transfer and ion-exchange interactions. Additionally, columns packed with silica particles excel at separating eluents with highly similar chemical and physical properties [43]. Nevertheless, the critical challenge in TCM chemical profiling is structural annotation.

**Fig. 5:**
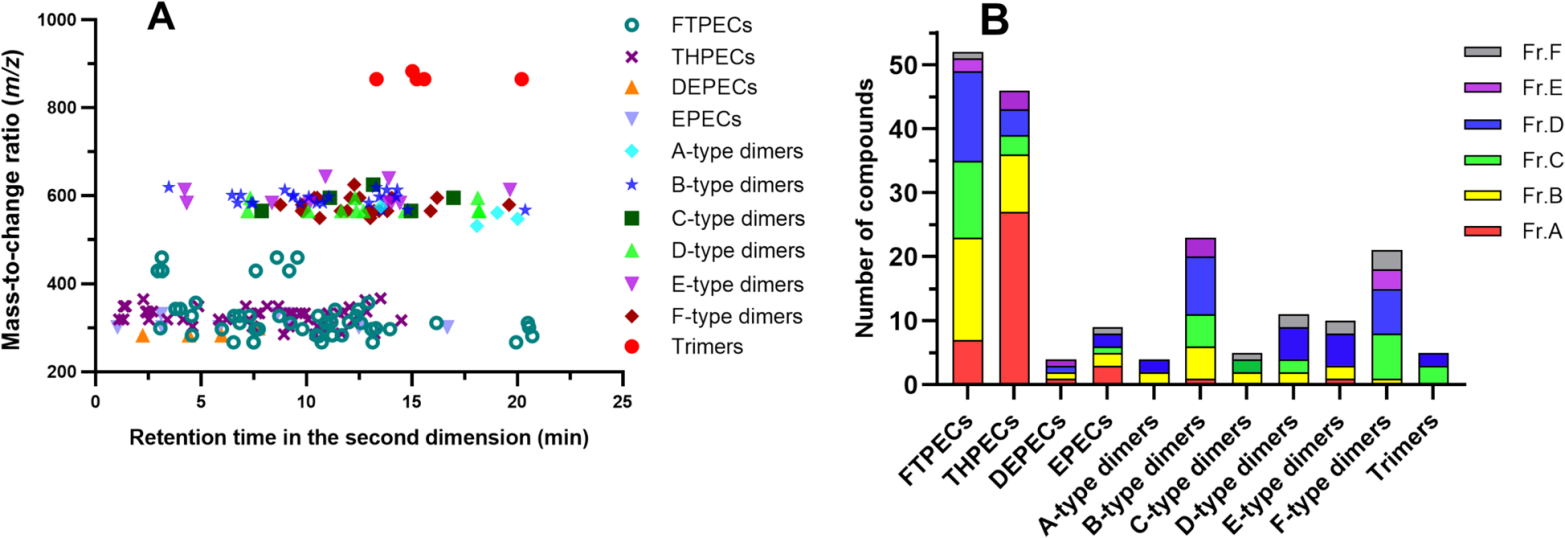
Structural diversity features of the characterized 2-(2-phenylethyl)chromones from Chinese agarwood (**A**-a scatter plot of different subtypes; **B**-comparison the number of different subtypes in different fractions)

In this work, the mass fragmentation patterns of D type PEC dimers, E type PEC dimers, and F type PEC dimers were firstly proposed to assist structural identification. In the MS spectrum, the monomer-derived fragment ions corresponding to the breakage of the ester bonds between two monomers in D type and E type dimers were the base peak in MS^2^. The HRF-initiated synergetic cleavage of the linkages of units A and B, RDA cracking of C and C′ ring, and the dissociation of the C_7′_–C_8′_ bond to derive ^6^A and ^6′^A were considered a diagnostic feature characteristic of F-type dimer. The calculated double bond equivalents could also provide clues for classifying PECs, as A type PEC dimers consistently exhibit 22 indices of hydrogen deficiency. D type and F type PEC dimers exhibited 21 degrees of unsaturation. At the meanwhile, B type and E type PEC dimers had 20 indices of hydrogen deficiency. Additionally, oligomers of PECs can be screened based on their higher molecular weight, typically exceeding 500 Da. The integration of a 2D-LC system with high-resolution IT-TOF–MS significantly enhanced the separation and characterization capabilities, leading to the identification of 199 PECs. The PECs identified from Chinese agarwood exhibits distinct structural diversity, including 11 distinct subtypes (Fig. 5). Among these compounds, 79 of them were PEC oligomers, accounting for 39.7% of the total. The THPECs have been demonstrated the unique distribution in agarwood and could be utilized as the diagnostic markers for authenticity and quality control, taking a proportion of 24.1% of the total amount. As shown in Fig. 5B, the FTPECs and THPECs are predominantly distributed in Fr. A and Fr. B, while PEC oligomers are mainly distributed in Fr. D, Fr. E, and Fr. F.

## Conclusion

Currently, it is challenging to comprehensively and in depth understand the chemical composition of herbal medicines, especially the trace components, owing to the characteristics of chemical diversity for herbal medicines, the limitation of the current phytochemical techniques and chromatographic resolution. Therefore, in the present study, an off-line 2D LC/UHPLC-IT-TOF/MS system was employed for the overall and in-depth comprehending of the chemical profiling of PECs in agarwood. Then, the mass fragmentation rules of PEC dimers were proposed for the first time using purified standards to assist structural identification. Afterwards, a total of 199 compounds, including 74 PEC dimers and five PEC trimers, were detected and structurally annotated from using 2D LC/UHPLC-IT-TOF/MS, of which 42 compounds were potential new compounds. Following chemical profiling, a total of eight trace 2-PEC dimers, including three pairs of scalemic mixtures (**IIa**/**IIb**, **IIIa**/**IIIb**, and **IVa**/**IVb**) and two optically pure PEC dimers (**I** and **V**) were acquired by LC/MS-guided procedure and definitely identified by analyzing the NMR spectroscopic dataset. Fortunately, all the definite identities were consistent with the structural annotation relied on LC–MS/MS. More importantly, the current study provided a feasible and efficient analytical strategy that is fully suitable for the rapid discovery of new PEC derivatives from Chinese agarwood, as well as other herbal medicines. The newly discovered PEC derivatives also laid the foundation for the elucidation of effective material basis of Chinese agarwood.

## Supplementary Information

Supplementary Material 1

## Data Availability

The datasets used and/or analyzed during the current study are available from the corresponding author on reasonable request.

## Abbreviations

LC–MS/MS: Liquid chromatography − mass spectrometry/mass spectrometry
PEC: 2-(2-Phenylethyl)chromone
NMR: Nuclear magnetic resonance
TCMs: Traditional Chinese medicines
COVID-19: Corona Virus Disease 2019
IT-TOF-MS: Hybrid ion trap time-of-flight mass spectrometer
LC–MS: Liquid chromatography-mass spectrometry
MS: Mass spectrometry
2D LC: Two-dimensional liquid chromatography
HPLC-DAD: High performance liquid chromatography-diode array detector
ACN: Acetonitrile
UHPLC: Ultra-high performance liquid chromatography
UV: Ultraviolet
CID: Collision-induced dissociation
ODS: Octadecylsilane
ECD: Electronic circular dichroism
RPLC: Reverse phase liquid chromatography
Frs.: Fractions
FTPECs: Flindersia type
THPECs: 5,6,7,8-Tetrahydro-2-(2-phenylethyl)chromones
DEPECs: 5,6:7,8-Diepoxy-2-(2-phenylethyl)chromones
EPECs: Mono-epoxy-2-(2-phenylethyl)chromones
RDA: Retro-diels–alder
HRESIMS: High resolution electron spray ionization mass spectrometry
HRF: Heterocylic ring fission
*t*_R_: Retention time
*m/z*: Mass-to-charge ratio
IR: Infrared spectroscopy
NOESY: Nuclear overhauser enhancement sepectroscophy
HMBC: Heteronuclear multiple bond coherencele
