# Supplementary material for: In-depth characterization of minor 2-(2-phenylethyl)chromone oligomers from Chinese agarwood by integrating offline two-dimensional liquid chromatography and hybrid ion trap time-of-flight mass spectrometry
Source: Chin Med. 2025 Feb 27;20:26. doi: 10.1186/s13020-025-01073-6 (PMC11866864; doi:10.1186/s13020-025-01073-6)
Supplement: Supplementary file 1 — Supplementary Material 1 [file 13020_2025_1073_MOESM1_ESM.docx]

**Supplemental information**

In-depth characterization of minor 2-(2-phenylethyl)chromone oligomers from Chinese agarwood by integrating offline two-dimensional liquid chromatography and hybrid ion trap time-of-flight mass spectrometry

Huixia Huo^a^, Hang Zhang ^a,b^, Huiting Liu ^a,b^, Jiale Ma ^a,b^, Qian Zhang^a^, Yunfang Zhao^a^, Jiao Zheng^a^, Pengfei Tu ^b^, Yuelin Song ^a,^*****, Jun Li ^b,^**^[[1]](#footnote-1)^***

**Contents**

**Fig. S1** The chemical structures of eighteen 2-(2-phenylethyl)chromone dimers used as reference in this study.

**Fig. S2** The ion nomenclature (A) and proposed mass fragmentation pathways of 2-(2-phenylethyl)chromone compounds (B: type I; C: type II; C: type III).

**Fig. S3** The main structures of 2-(2-phenylethyl)chromone dimers (A-type, B-type, C-type, C-type, D-type, and F-type).

**Fig. S4** The ion nomenclature (A), proposed mass fragmentation pathways(B), and the MS/MS of chemical reference standard(C) of aquisinenone I (representative E-type dimer).

**Fig. S5** Selected HMBC (arrows point from protons to carbons) and ^1^H−^1^H COSY correlations of compounds I–V.

**Fig. S6** Selected ROESY correlations of compounds I–V.

**Fig. S7** Experimental and calculated ECD spectra of compounds I–V (in MeOH).

**Table S1** Retention times, mass spectral data, molecular formulae, and plausible identities of detected compounds in Chinese agarwood using UHPLC-PDA-IT-TOF-MS.

*Structural elucidation of PECs by LC/MS*

*Purification of new PEC dimers*

*Physicochemical Properties of New Compounds*

*MS, UV, IR, and NMR Spectra of New Compounds*

**Fig. S1** The chemical structures of eighteen 2-(2-phenylethyl)chromone dimers used as reference in this study.

crassin A (**1**), aquisinenone N (**2**), aquisinenone G (**3**), 4′-methoxy-aquisinenone G (**4**), aquisinenone A (**5**), 4′-methoxyaquisinenone A (**6**), aquisinenone B (**7**), 6′′-hydroxy- aquisinenone B (**8**), 6′′-hydroxy-4′,4′′′-dimethoxyaquisinenone B (**9**), aquisinenone C (**10**), aquisinenone D (**11**), 4′-demethoxyaquisinenone D (**12**), aquisinenone E (**13**), aquisinenone F (**14**), 4′,4‴-dimethoxyaquisinenone K (**15**), aquisinenone I (**16**), 7″-methoxyaquisinenone I (**17**), and 4′,7″-dimethoxyaquisinenone I (**18**).

**Fig. S2** The ion nomenclature (A) and proposed mass fragmentation pathways of 2-(2-phenylethyl)chromone compounds (B: type I; C: type II; D: type III).

**Fig. S3** The main structures of 2-(2-phenylethyl)chromone dimers (A-type, B-type, C-type, D-type, E-type, and F-type)

**Fig. S4** The ion nomenclature (A), proposed mass fragmentation pathways(B), and the MS/MS of chemical reference standard(C) of aquisinenone I (representative E-type dimer)

**Fig. S5** Selected HMBC (arrows point from protons to carbons) and ^1^H−^1^H COSY correlations of compounds **I**–**V**.

**Fig. S6** Selected ROESY correlations of compounds **I**–**V**.


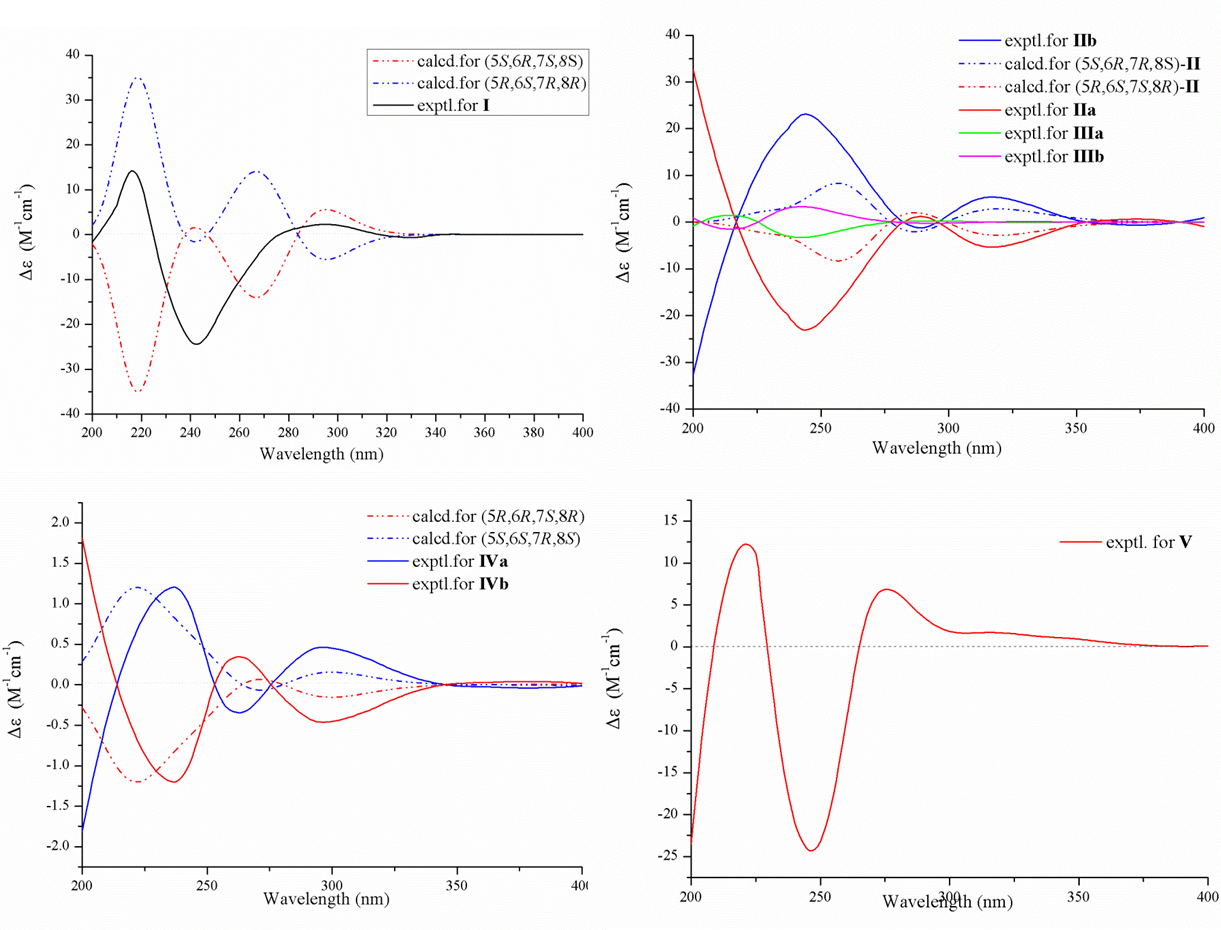


**Fig. S7** Experimental and calculated ECD spectra of compounds **I**–**V** (in MeOH).

**Table S1**. Retention Times, Mass Spectral Data, Molecular Formulae, and Plausible Identities of Detected Compounds in Chinese agarwood Using UHPLC-PDA-IT-TOF-MS

| **No.** | ***t*_R_**  **(min)** | | | **[M + H]+** | | **Error**  **(ppm)** | | **Molecular Formulae** | | **MS/MS** | **Substituent** | | | **Plausible identity** |
| --- | --- | --- | --- | --- | --- | --- | --- | --- | --- | --- | --- | --- | --- | --- |
|  |  |  |  |  |  |  |  |  |  |  | **Type** | **A-ring** | **B-ring** |  |
| **Fr.A** | | | | | | | | | | |  |  |  |  |
| **1** | | 4.480 | | 319.1180 | | 1.25 | | C_17_H_18_O_6_ | | 301, 255, 227 | II | – | – | 5,6,7,8-tetrahydroxy-2-(2-phenylethyl)-5,6,7,8-tetrahydrochromone or isomer |
| **2** | | 5.838 | | 319.1155 | | –6.58 | | C_17_H_18_O_6_ | | 301, 255, 227 | II | – | – | 5,6,7,8-tetrahydroxy-2-(2-phenylethyl)-5,6,7,8-tetrahydrochromone or isomer |
| **3** | | 5.870 | | 301.1064 | | –2.32 | | C_17_H_16_O_5_ | | 283, 255, 227, 164 | IV | – | – | 5,6-epoxy-7,8-dihydroxy-2-(2-phenylethyl)-5,6,7,8-tetrahydrochromone or isomer |
| **4** | | 5.962 | | 283.0975 | | 3.53 | | C_17_H_14_O_4_ | | 255, 227, 192, 164 | III | – | – | 5,6:7,8-diepoxy-2-(2-phenylethyl)-5,6,7,8-tetrahydrochromone or isomer |
| **5** | | 6.205 | | 319.1151 | | –7.83 | | C_17_H_18_O_6_ | | 301, 283, 255, 173 | II | – | – | 5,6,7,8-tetrahydroxy-2-(2-phenylethyl)-5,6,7,8-tetrahydrochromone or isomer |
| **6** | | 7.122 | | 349.1254 | | –8.02 | | C_18_H_20_O_7_ | | 331, 313, 285, 121 | II | – | OCH_3_ | 5,6,7,8-tetrahydroxy-2-[2-(4′ methoxyphenyl)ethyl]-5,6,7,8-tetrahydrochromone or isomer |
| **7** | | 7.275 | | 319.1146 | | –9.40 | | C_17_H_18_O_6_ | | 301, 283, 255, 227, 164 | II | – | – | 5,6,7,8-tetrahydroxy-2-(2-phenylethyl)-5,6,7,8-tetrahydrochromone or isomer |
| **8** | | 7.758 | | 333.1277 | | –16.81 | | C_18_H_20_O_6_ | | 301, 283, 255 | II | OCH_3_ |  | 5,6,7-trihydroxy-8-methoxy-5,6,7,8-tetrahydro-2-(2-phenylethyl)chromone or isomer |
| **9** | | 8.128 | | 349.1282 | | 0 | | C_18_H_20_O_7_ | | 331, 313, 285, 121 | II | – | OCH_3_ | 5,6,7,8-tetrahydroxy-2-[2-(4′ methoxyphenyl)ethyl]-5,6,7,8-tetrahydrochromone or isomer |
| **10** | | 8.602 | | 331.1167 | | –2.72 | | C_18_H_18_O_6_ | | 313, 285, 121 | IV | – | OCH_3_ | rel-(1a*R*,2*R*,3*R*,7b*S*)-1a,2,3,7b-tetrahydro-2,3-dihydroxy-5-[2-(4-methoxyphenyl)ethyl]-7H-oxireno[f][1]benzopyran-7-one or isomer |
| **11** | | 8.665 | | 349.1227 | | –15.75 | | C_18_H_20_O_7_ | | 331, 313, 285, 121 | II | – | OCH_3_ | 5,6,7,8-tetrahydroxy-2-[2-(4′ methoxyphenyl)ethyl]-5,6,7,8-tetrahydrochromone or isomer |
| **12** | | 8.918 | | 285.1105 | | –5.61 | | C_17_H_16_O_4_ | | 267, 239, 148, | II | – | – | 5,6-dihydroxy-2-phenethyl-5,6-dihydro-4*H*-chromen-4-one or isomer |
| **13** | | 9.162 | | 303.1207 | | –6.60 | | C_17_H_18_O_5_ | | 285, 267, 239 | II | – | – | 5,6,7-trihydroxy-2-(2-phenylethyl)-5,6,7,8-tetrahydrochromone or isomer |
| **14** | | 9.193 | | 333.1309 | | –7.20 | | C_18_H_20_O_6_ | | 301, 283, 255 | II | OCH_3_ |  | 5,6,7-trihydroxy-8-methoxy-5,6,7,8-tetrahydro-2-(2-phenylethyl)chromone or isomer |
| **15** | | 9.352 | | 333.1370 | | 11.11 | | C_18_H_20_O_6_ | | 315, 297, 269, 121 | II | – | OCH_3_ | 5,6,7-trihydroxy-2-[2-(4′-methoxyphenyl)ethyl]-5,6,7,8-tetrahydrochromone or isomer |
| **16** | | 9.70 | | 333.1317 | | –4.80 | | C_18_H_20_O_6_ | | 301, 283, 255 | II | OCH_3_ |  | 5,6,7-trihydroxy-8-methoxy-5,6,7,8-tetrahydro-2-(2-phenylethyl)chromone or isomer |
| **17** | | 9.922 | | 333.1311 | | –6.60 | | C_18_H_20_O_6_ | | 315, 121 | II | – | OCH_3_ | 5,6,7-trihydroxy-2-[2-(4′-methoxyphenyl)ethyl]-5,6,7,8-tetrahydrochromone or isomer |
| **18** | | 10.480 | | 283.0960 | | –1.77 | | C_17_H_14_O_4_ | | 192, 153, 91 | I | OH, OH | – | 6,8-dihydroxy-2-(2-phenylethyl)chromone or isomer |
| **19** | | 10.522 | | 303.1202 | | –8.25 | | C_17_H_18_O_5_ | | 285, 267, 239 | II | – | – | 5,6,7-trihydroxy-2-(2-phenylethyl)-5,6,7,8-tetrahydrochromone or isomer |
| **20** | | 10.710 | | 267.1008 | | –3.00 | | C_17_H_14_O_3_ | | 176, 137,148, 110 | I | OH | – | 6-hydroxy-2-(2-phenylethyl)chromone or isomer |
| **21** | | 11.022 | | 333.1291 | | –12.61 | | C_18_H_20_O_6_ | | 315, 283, 255, 227 | II | OCH_3_ |  | 5,6,7-trihydroxy-8-methoxy-5,6,7,8-tetrahydro-2-(2-phenylethyl)chromone or isomer |
| **22** | | 11.053 | | 303.1234 | | 1.98 | | C_17_H_18_O_5_ | | 285, 267, 239 | II | – | – | 5,6,7-trihydroxy-2-(2-phenylethyl)-5,6,7,8-tetrahydrochromone or isomer |
| **23** | | 11.537 | | 333.1307 | | –7.80 | | C_18_H_20_O_6_ | | 315, 297, 121 | II | – | OCH_3_ | 5,6,7-trihydroxy-2-[2-(4′-methoxyphenyl)ethyl]-5,6,7,8-tetrahydrochromone or isomer |
| **24** | | 11.662 | | 287.1263 | | –5.22 | | C_17_H_18_O_4_ | | 269, 251, 160 | II | – | – | 6,7-cis-dihydroxy-2-(2-phenylethyl)-5,6,7,8-tetrahydrochromone or isomer |
| **25** | | 11.757 | | 337.0832 | | –1.48 | | C_17_H_17_ClO_5_ | | 319, 301, 283, 273, 265, 255 | II | – | – | 8-chloro-5,6,7-trihydroxy-2-(2-phenylethyl)-5,6,7,8-tetrahydrochromone or isomer |
| **26** | | 12.032 | | 347.1488 | | –0.29 | | C_19_H_22_O_6_ | | 315, 283, 255, 227 | II | OCH_3_  OCH_3_ |  | 6,7-dihydroxy-5,8-dimethoxy-5,6,7,8-tetrahydro-2-(2-phenylethyl)chromone or isomer |
| **27** | | 12.217 | | 317.1339 | | –14.19 | | C_18_H_20_O_5_ | | 299, 281, 121 | II | – | OCH_3_ | 6,7-cis-dihydroxy-5,6,7,8-tetrahydro-2-(2-(4-methoxyphenyl)ethyl)chromone or isomer |
| **28** | | 12.783 | | 363.1407 | | -8.54 | | C_19_H_22_O_7_ | | 345, 313, 285, 121 | II | OCH_3_ | OCH_3_ | 5,6,7-trihydroxy-8-methoxy-5,6,7,8-tetrahydro-2-[2-(4′-methoxyphenyl)ethyl]chromone or isomer |
| **29** | | 12.847 | | 337.0821 | | –5.04 | | C_17_H_17_ClO_5_ | | 319, 301, 283, 265, 255 | II | – | – | 8-chloro-5,6,7-trihydroxy-2-(2-phenylethyl)-5,6,7,8-tetrahydrochromone or isomer |
| **30** | | 13.227 | | 287.1257 | | –6.97 | | C_17_H_18_O_4_ | | 269, 251, 160 | II | – | – | 6,7-cis-dihydroxy-2-(2-phenylethyl)-5,6,7,8-tetrahydrochromone or isomer |
| **31** | | 13.290 | | 299.0894 | | –6.69 | | C_17_H_14_O_5_ | | 193, 107 | I | OH, OH | OH | 5,7-dihydroxy-2-[2-(4-hydroxyphenyl)ethyl]chromone or isomer |
| **32** | | 13.512 | | 367.0948 | | 1.36 | | C_18_H_19_ClO_6_ | | 349, 331, 121 | II | – | OCH_3_ | rel-(5*R*,6*S*,7*S*,8*R*)-8-Chloro-5,6,7,8-tetrahydro-5,6,7-trihydroxy-2-[2-(4-methoxyphenyl)ethyl]-4H-1-benzopyran-4-one or isomer |
| **33** | | 14.492 | | 317.1403 | | 5.99 | | C_18_H_20_O_5_ | | 299, 281, 121 | II | – | OCH_3_ | 6,7-cis-dihydroxy-5,6,7,8-tetrahydro-2-(2-(4-methoxyphenyl)ethyl)chromone or isomer |
| **34** | | 16.675 | | 301.1056 | | –4.98 | | C_17_H_16_O_5_ | | 283, 255, 227, 164 | IV | – | – | 5,6-epoxy-7,8-dihydroxy-2-(2-phenylethyl)-5,6,7,8-tetrahydrochromone or isomer |
| **35** | | 19.650 | | 613.2042 | | –4.24 | | C_35_H_32_O_10_ | | 595, 577, 441, 379, 313*, 283 | E |  |  | methoxy aquisinenone K or isomer |
| **36** | | 19.933 | | 267.1019 | | 1.12 | | C_17_H_14_O_3_ | | 176, 137, 110, 91 | I | OH | – | 6-hydroxy-2-(2-phenylethyl)chromone or isomer |
| **37** | | 20.365 | | 567.1918 | | –16.75 | | C_34_H_30_O_8_ | | 549, 531, 513, 357, 283, 267 | B |  |  | AH_13_ or isomer |
| **38** | | 20.488 | | 311.1252 | | –8.63 | | C_19_H_18_O_4_ | | 220, 205, 177 | I | OCH_3,_ OCH_3_ | – | 6,7-dimethoxy-2-(2-phenylethyl)chromone or isomer |
| **39** | | 20.548 | | 301.0604 | | –7.31 | | C_17_H_13_ClO_3_ | | 210, 182 | I | Cl, OH | – | 8-chloro-6-hydroxy-2-(2-phenylethyl)chromone or isomer |
| **40** | | 20.705 | | 281.1147 | | –8.89 | | C_18_H_16_O_3_ | | 190, 151 | I | OCH_3,_ | – | 6-methoxy-2-(2-phenylethyl)chromone or isomer |
| **Fr.B** | | | | | | | | | | |  |  |  |  |
| **41** | | | 2.263 | | 365.1212 | | –5.20 | | C_18_H_20_O_8_ | 347, 329, 301, 137 | II | – | OCH_3_, OH | 5,6,7,8-tetrahydroxy-2-[2-(3′-hydroxy-4′-methoxyphenyl) ethyl]-5,6,7,8-tetrahydrochromone or isomer |
| **42** | | | 2.422 | | 335.1099 | | –7.76 | | C_17_H_18_O_7_ | 317, 299, 271, 243 | II | – | OH | aquilarone F |
| **43** | | | 3.392 | | 319.1151 | | –7.83 | | C_17_H_18_O_6_ | 301, 283, 255, 227, 164 | II | – | – | 5,6,7,8-tetrahydroxy-2-(2-phenylethyl)-5,6,7,8-tetrahydrochromone or isomer or isomer |
| **44** | | | 4.143 | | 319.1152 | | –7.52 | | C_17_H_18_O_6_ | 301, 283, 255, 227, 164 | II | – | – | 5,6,7,8-tetrahydroxy-2-(2-phenylethyl)-5,6,7,8-tetrahydrochromone or isomer |
| **45** | | | 4.585 | | 303.1221 | | –5.28 | | C_17_H_18_O_5_ | 285, 267, 239, 148, 91 | II | – | – | 5,6,7-trihydroxy-2-(2-phenylethyl)-5,6,7,8-tetrahydrochromone or isomer |
| **46** | | | 4.870 | | 349.1277 | | –1.43 | | C_18_H_20_O_7_ | 331, 313, 285, 121 | II | – | OCH_3_ | 5,6,7,8-tetrahydroxy-2-[2-(4′-methoxyphenyl)ethyl]-5,6,7,8-tetrahydrochromone or isomer |
| **47** | | | 7.435 | | 303.1199 | | –9.24 | | C_17_H_18_O_5_ | 285, 267, 239 | II | – | – | 5,6,7-trihydroxy-2-(2-phenylethyl)-5,6,7,8-tetrahydrochromone or isomer |
| **48** | | | 7.593 | | 429.1536 | | –1.86 | | C_23_H_24_O_8_ | 267, 176, 137, 91 | I | OH | – | 2-(2-phenylethyl)chromone-8-*O*-β-D-glucopyranoside or isomer |
| **49** | | | 7.720 | | 299.0930 | | 5.35 | | C_17_H_14_O_5_ | 253, 209, 162, 123, 91 | III | – | R_4_=OH | 5,6:7,8-diepoxy-2-[2-(7′-hydroxyphenyl)ethyl]-5,6,7,8-tetrahydrochromone or isomer |
| **50** | | | 7.625 | | 333.1340 | | 2.10 | | C_18_H_20_O_6_ | 315, 287, 269, 121 | II | – | OCH_3_ | 5,6,7-trihydroxy-2-[2-(4′-methoxyphenyl)ethyl]-5,6,7,8-tetrahydrochromone or isomer |
| **51** | | | 8.587 | | 459.1632 | | –3.92 | | C_24_H_26_O_9_ | 297, 167 | I | OCH_3_, OH | – | methoxy 2-(2-phenylethyl)chromone-8-O-β-D-glucopyranoside or isomer |
| **52** | | | 8.998 | | 331.1144 | | –9.66 | | C_18_H_18_O_6_ | 313, 285, 121 | IV | – | OCH_3_ | rel-(1aR,2R,3R,7bS)-1a,2,3,7b-tetrahydro-2,3-dihydroxy-5-[2-(4-methoxyphenyl)ethyl]-7H-oxireno[f][1]benzopyran-7-one or isomer |
| **53** | | | 9.178 | | 429.1581 | | 8.62 | | C_23_H_24_O_8_ | 267, 176, 137 | I | OH | – | 2-(2-phenylethyl)chromone-8-*O*-β-D-glucopyranoside or isomer |
| **54** | | | 9.210 | | 317.1031 | | 3.47 | | C_17_H_16_O_6_ | 285, 257, 227, 107 | IV | – | OH | 5,6-epoxy-7,8-dihydroxy-2-[2-(4′-hydroxyphenyl)ethyl]-5,6,7,8-tetrahydrochromone or isomer |
| **55** | | | 9.557 | | 459.1644 | | –1.31 | | C_24_H_26_O_9_ | 297, 206, 167, 121 | I | OCH_3_, OH | – | methoxy 2-(2-phenylethyl)chromone-8-*O*-β-D-glucopyranoside or isomer |
| **56** | | | 9.683 | | 583.2134 | | 29.32 | | C_31_H_34_O_11_ | 547, 519, 371, 283 | B |  |  | AH_15_ or isomer |
| **57** | | | 10.408 | | 285.1094 | | –9.47 | | C_17_H_16_O_4_ | 267, 239, 176, 137 | II | – | – | 5,6-dihydroxy-2-phenethyl-5,6-dihydro-4H-chromen-4-one or isomer |
| **58** | | | 10.933 | | 313.1046 | | –7.98 | | C_18_H_16_O_5_ | 192, 121 | I | OH, OH | OCH_3_ | 6,7-dihydroxy-2-[2-(4′-methoxyphenyl)ethyl]chromone (isomer 1) |
| **59** | | | 11.232 | | 283.0907 | | –20.49 | | C_17_H_14_O_4_ | 177,137, 107 | I | OH | OH | 6-hydroxy-2-[2-(4-hydroxyphenyl)ethyl]chromone or isomer |
| **60** | | | 11.200 | | 283.0943 | | –7.77 | | C_17_H_14_O_4_ | 192, 153 | I | OH, OH | – | 6,8-dihydroxy-2-(2-phenylethyl)chromone or isomer |
| **61** | | | 11.175 | | 313.1053 | | –5.75 | | C_18_H_16_O_5_ | 177, 137, 121 | I | OH | OH, OCH_3_ | 6-hydroxy-2-[2-(4′-hydroxy-3′-methoxyphenyl)ethenyl]chromone or isomer |
| **62** | | | 11.642 | | 565.1944 | | 15.39 | | C_34_H_28_O_8_ | 547, 529, 474, 283*, 192 | D |  |  | aquisinenone G or isomer |
| **63** | | | 11.672 | | 283.0924 | | –14.48 | | C_17_H_14_O_4_ | 192, 153 | I | OH, OH | – | 5,8-dihydroxy-2-(2-phenylethyl)chromone or isomer |
| **64** | | | 12.007 | | 313.1075 | | 1.28 | | C_18_H_16_O_5_ | 121 | I | OH, OH | OCH_3_ | 6,7-dihydroxy-2-[2-(4′-methoxyphenyl)ethyl]chromone or isomer |
| **65** | | | 12.353 | | 327.1198 | | –8.87 | | C_19_H_18_O_5_ | 221, 181, 205 | I | OCH_3_, OCH_3_ | OH | 6,7-dimethoxy-2-[2-(4-hydroxyphenyl)ethyl]chromone or isomer |
| **66** | | | 12.918 | | 357.1336 | | 0.84 | | C_20_H_20_O_6_ | 220, 137, 122 | I | OCH_3_, OCH_3_ | OH, OCH_3_ | 6,7-dimethoxy-2-[2-(3′-hydroxy-4′-methoxyphenyl)ethyl]chromone or isomer |
| **67** | | | 12.950 | | 583.1881 | | –14.06 | | C_34_H_30_O_9_ | 565, 547, 519, 395, 327, 283 | B |  |  | AH_15_ or isomer |
| **68** | | | 13.073 | | 297.1076 | | –15.15 | | C_18_H_16_O_4_ | 206, 167 | I | OH, OCH_3_ | – | 6-hydroxy-7-methoxy-2-(2-phenylethyl)chromone or isomer |
| **69** | | | 13.137 | | 267.1001 | | –5.62 | | C_17_H_14_O_3_ | 176, 137, 91 | I | OH, | – | 6-hydroxy-2-(2-phenylethyl)chromone or isomer |
| **70** | | | 13.163 | | 625.2001 | | –10.72 | | C_36_H_32_O_10_ | 607, 589, 343 | C |  |  | dimethoxy AH_21、_or isomer |
| **71** | | | 13.517 | | 575.1976 | | –15.30 | | C_36_H_30_O_7_ | 267, 176, 137 | A |  |  | dehydroxy-dimethoxy-AH_11_ |
| **72** | | | 13.768 | | 583.1897 | | –11.32 | | C_34_H_30_O_9_ | 565, 547, 301, 283*, 192, 153 | E |  |  | aquisinenone K or isomer |
| **73** | | | 13.800 | | 613.2089 | | 3.42 | | C_35_H_32_O_10_ | 595, 577, 475, 415, 313, 283, 121 | B |  |  | methoxy AH_15_ or isomer |
| **74** | | | 13.952 | | 297.1111 | | –3.37 | | C_18_H_16_O_4_ | 121 | I | OH | OCH_3_ | 6-hydroxy-2-[2-(4′-methoxyphenyl)ethyl]chromone or isomer |
| **75** | | | 14.298 | | 613.2054 | | –2.28 | | C_35_H_32_O_10_ | 595, 577, 313, 283, 121 | B |  |  | methoxy AH_15_ or isomer |
| **76** | | | 14.425 | | 583.1913 | | –8.57 | | C_34_H_30_O_9_ | 547, 519, 435, 283* | E |  |  | aquisinenone K or isomer |
| **77** | | | 14.678 | | 565.1816 | | –7.25 | | C_34_H_28_O_8_ | 547.529, 373, 283*, 192 | D |  |  | aquisinenone G or isomer |
| **78** | | | 14.805 | | 567.1967 | | –8.11 | | C_34_H_30_O_8_ | 549, 531, 375, 283 | B |  |  | AH_13_ or isomer |
| **79** | | | 16.163 | | 311.1271 | | –2.25 | | C_19_H_18_O_4_ | 220, 205 | I | OCH_3，_ OCH_3_ |  | 6,7-dimethoxy-2-(2-phenylethyl)chromone or isomer |
| **80** | | | 16.193 | | 595.1940 | | –3.86 | | C_35_H_30_O_9_ | 577, 503, 474*, 385, 283 | F |  |  | methoxy 6′′-hydroxy-aquisinenone B or isomer |
| **81** | | | 16.960 | | 595.1910 | | –8.9 | | C_35_H_30_O_9_ | 577, 559, 549, 283, 192, | C |  |  | methoxy AH_21_ or isomer |
| **82** | | | 20.005 | | 547.1693. | | –10.60 | | C_34_H_26_O_7_ | 529, 456, 373, 282, 267, 176, 137 | A |  |  | hydroxy AH_11_ |
| **Fr.C** | | |  | |  | |  | |  |  |  |  |  |  |
| **83** | | | 1.038 | | 301.1059 | | –3.99 | | C_17_H_16_O_5_ | 283, 255, 227, 164 | IV | – | – | 5,6-epoxy-7,8-dihydroxy-2-(2-phenylethyl)-5,6,7,8-tetrahydrochromone or isomer |
| **84** | | | 1.323 | | 349.1280 | | –0.57 | | C_18_H_20_O_7_ | 331, 313, 285, 121 | II | – | OCH_3_ | 5,6,7,8-tetrahydroxy-2-[2-(4′-methoxyphenyl)ethyl]-5,6,7,8-tetrahydrochromone or isomer |
| **85** | | | 2.463 | | 337.0846 | | 2.67 | | C_17_H_17_O_5_Cl | 319, 301, 265 | II | – | – | 8-chloro-5,6,7-trihydroxy-2-(2-phenylethyl)-5,6,7,8-tetrahydrochromone or isomer |
| **86** | | | 2.527 | | 319.1205 | | 9.09 | | C_17_H_18_O_6_ | 301, 255 | II | – | – | 5,6,7,8-tetrahydroxy-2-(2-phenylethyl)-5,6,7,8-tetrahydrochromone or isomer |
| **87** | | | 2.938 | | 429.1504 | | –9.32 | | C_23_H_24_O_8_ | 267, 176, 91 | I | OH | – | 2-(2-phenylethyl)chromone-8-*O*-β-D-glucopyranoside or isomer |
| **88** | | | 3.128 | | 459.1631 | | –4.14 | | C_24_H_26_O | 297, 206, 167 | I | OCH_3_, OH | – | methoxy 2-(2-phenylethyl)chromone-8-O-β-D-glucopyranoside or isomer |
| **89** | | | 3.160 | | 429.1512 | | –7.46 | | C_23_H_24_O_8_ | 267, 176, 137 | I | OH | – | 2-(2-phenylethyl)chromone-8-*O*-β-D-glucopyranoside or isomer |
| **90** | | | 4.005 | | 343.1177 | | 0.29 | | C_19_H_18_O_6_ | 207, 137 | I | OCH_3_, OH | OCH_3_, OH | 7-hydroxy-6-methoxy-2-[2-(3′-hydroxy-4′-methoxyphenyl)ethyl]chromone or isomer |
| **91** | | | 4.567 | | 283.0941 | | –8.48 | | C_17_H_14_O_4_ | 192, 153 | I | OH, OH | – | 5,8-dihydroxy-2-(2-phenylethyl)chromone or isomer |
| **92** | | | 5.977 | | 297.1135 | | 4.71 | | C_18_H_16_O_4_ | 191, 176 | I | OCH_3_, | OH | 4′-hydroxy-6-methoxy-2-(2-phenyl)ethylchromone or isomer |
| **93** | | | 6.535 | | 267.1000 | | –5.99 | | C_17_H_14_O_3_ | 161, 107 | I | – | OH | 2-[2-(3-hydroxyphenyl)ethyl]chromone or isomer |
| **94** | | | 6.567 | | 327.1206 | | –6.42 | | C_19_H_18_O_5_ | 191, 176 | I | OCH_3_, | OCH_3_, OH | 6-methoxy-2-[2-(3-methoxy-4-hydroxyphenyl)ethyl]chromone or isomer |
| **95** | | | 7.463 | | 583.1875 | | –15.09 | | C_34_H_30_O_9_ | 565, 547, 519, 355,283 | B |  |  | AH_15_ or isomer |
| **96** | | | 7.495 | | 267.1006 | | –3.37 | | C_17_H_14_O_3_ | 176, 91 | I | OH | – | 6-hydroxy-2-(2-phenylethyl)chromone or isomer |
| **97** | | | 7.558 | | 297.1104 | | –5.72 | | C_18_H_16_O_4_ | 282, 206, 167 | I | OH, OCH_3_ | – | 6-hydroxy-7-methoxy-2-(2-phenylethyl)chromone or isomer |
| **98** | | | 8.700 | | 327.1200 | | –7.95 | | C_19_H_18_O_5_ | 206, 121 | I | OH, OCH_3_, | OCH_3_ | 7-hydroxy-6-methoxy-2-[2-(4-methoxyphenyl)ethyl]-4H-1-benzopyran-4-one or isomer |
| **99** | | | 9.855 | | 565.1889 | | 5.66 | | C_34_H_28_O_8_ | 547, 529, 474*, 373, 283，192 | F |  |  | 6′′-hydroxyaquisinenone B or isomer |
| **100** | | | 10.077 | | 565.1859 | | 0.35 | | C_34_H_28_O_8_ | 547, 529, 435, 283*, 255 | D |  |  | aquisinenone G or isomer |
| **101** | | | 10.362 | | 595.1904 | | –5.9 | | C_35_H_30_O_9_ | 577, 559, 489, 471, 368, 293, 297 | F |  |  | hydroxy and methoxy aquisinenone A or isomer |
| **102** | | | 11.045 | | 597.2092 | | –4.52 | | C_35_H_32_O_9_ | 565, 547, 283, 192 | B |  |  | 5-methoxy-AH_13_ isomer |
| **103** | | | 11.108 | | 595.1970 | | 1.18 | | C_35_H_30_O_9_ | 577, 559, 283, 153 | C |  |  | methoxyAH_21_ or isomer |
| **104** | | | 12.577 | | 311.1252 | | –8.36 | | C_19_H_18_O_4_ | 220, 205, 181 | I | OCH_3_, OCH_3_ | – | 6,7-dimethoxy-2-(2-phenylethyl)chromone or isomer |
| **105** | | | 12.952 | | 565.1805 | | –9.20 | | C_34_H_28_O_8_ | 529, 474, 283 | F |  |  | 6′′-hydroxyaquisinenone B or isomer |
| **106** | | | 13.318 | | 865.2855 | | 0.81 | | C_51_H_44_O_13_ | 847, 829, 811, 565, 547, 529, 283 |  |  |  | tri-2-(2-phenylethyl)chromone or isomer |
| **107** | | | 13.450 | | 597.2087 | | –5.19 | | C_35_H_32_O_9_ | 579, 561, 297 | B |  |  | AH_12_ or isomer |
| **108** | | | 13.543 | | 567.1989 | | –4.23 | | C_34_H_30_O_8_ | 549, 531, 283, 267, 176 | B |  |  | AH_13_ or isomer |
| **109** | | | 13.820 | | 565.1812 | | –7.96 | | C_34_H_28_O_8_ | 529, 474, 283 | F |  |  | 6′′-hydroxyaquisinenone B or isomer |
| **110** | | | 14.038 | | 595.1947 | | –2.69 | | C_35_H_30_O_9_ | 577, 474*, 373, 283 | F |  |  | methoxy 6′′-hydroxyaquisinenone B or isomer |
| **111** | | | 14.258 | | 597.2080 | | –6.53 | | C_35_H_32_O_9_ | 579, 561, 297 | B |  |  | AH_12_ or isomer |
| **112** | | | 14.955 | | 565.1856 | | –0.18 | | C_34_H_28_O_8_ | 547, 529, 474, 283, 192 | C |  |  | AH_21_ or isomer |
| **113** | | | 15.018 | | 883.2913 | | –5.32 | | C_51_H_46_O_14_ | 865, 847, 829, 811, 583, 565, 547, 519, 283, 192 |  |  |  | tri-2-(2-phenylethyl)chromone |
| **114** | | | 15.570 | | 865.2814 | | –4.74 | | C_51_H_44_O_13_ | 847, 829, 811, 565, 501, 319, 267 |  |  |  | tri-2-(2-phenylethyl)chromone or isomer |
| **115** | | | 15.887 | | 565.1881 | | 4.25 | | C_34_H_28_O_8_ | 547, 529, 438, 283, 192 | F |  |  | aquisinenone C or isomer |
| **116** | | | 18.205 | | 565.1891 | | 6.02 | | C_34_H_28_O_8_ | 547, 474, 283*, 192 | D |  |  | aquisinenone G or isomer |
| **117** | | | 19.607 | | 579.2018 | | 0.86 | | C_34_H_28_O_8_ | 561, 515, 487, 458, 394, 357, 303, 266 | F |  |  | 4′-methoxyaquisinenone A or isomer |
| **Fr.D** | | | | | | | | | | |  |  |  |  |
| **118** | | | 1.105 | | 319.1143 | | –8.46 | | C_17_H_18_O_6_ | 301, 283, 255,164 | II | – | – | 5,6,7,8-tetrahydroxy-2-(2-phenylethyl)-5,6,7,8-tetrahydrochromone or isomer |
| **119** | | | 1.422 | | 349.1301 | | 5.44 | | C_18_H_20_O_7_ | 331, 313, 285 | II | – | OCH_3_ | 5,6,7,8-tetrahydroxy-2-[2-(4′-methoxyphenyl)ethyl] -5,6,7,8-tetrahydrochromone or isomer |
| **120** | | | 2.688 | | 337.0833 | | –1.19 | | C_17_H_17_O_5_Cl | 319, 301, 283, 265 | II | – | – | 8-chloro-5,6,7-trihydroxy-2-(2-phenylethyl)-5,6,7,8-tetrahydrochromone or isomer |
| **121** | | | 3.068 | | 299.0921 | | 2.34 | | C_17_H_14_O_5_ | 208, 169 | I | OH, OH, OH | – | trihydroxy-2-(2-phenylethyl)chromone |
| **122** | | | 3.100 | | 331.1184 | | 2.42 | | C_18_H_18_O_6_ | 313, 285, 121 | IV | – | OCH_3_ | rel-(1aR,2R,3R,7bS)-1a,2,3,7b-tetrahydro-2,3-dihydroxy-5-[2-(4-methoxyphenyl)ethyl]-7H-oxireno[f][1]benzopyran-7-one or isomer |
| **123** | | | 3.163 | | 301.1059 | | –3.99 | | C_17_H_16_O_5_ | 283, 255, 227 | IV | – | – | rel-(1aR,2R,3R,7bS)-1a,2,3,7b-Tetrahydro-2,3-dihydroxy-5-(2-phenylethyl)-7H-oxireno[f][1]benzopyran-7-one or isomer |
| **124** | | | 3.470 | | 619.2215 | | 6.62 | | C_34_H_34_O_11_ | 601, 301, 255, 227 | B |  |  | bi-phenylethylchromones I or isomer |
| **125** | | | 3.787 | | 343.1205 | | 8.45 | | C_19_H_18_O_6_ | 207, 137 | I | OH, OCH_3_ | OH, OCH_3_ | 7-hydroxy-6-methoxy-2-[2-(3′-hydroxy-4′-methoxyphenyl)ethyl]chromone or isomer |
| **126** | | | 4.420 | | 283.0946 | | –6.71 | | C_17_H_14_O_4_ | 255, 227, 192, 164 | III | – | – | 5,6:7,8-diepoxy-2-(2-phenylethyl)-5,6,7,8-tetrahydrochromone or isomer |
| **127** | | | 4.547 | | 327.1235 | | 2.46 | | C_19_H_18_O_5_ | 221, 205 | I | OCH_3_, OCH_3_ | OH | 6,7-dimethoxy-2-[2-(4-hydroxyphenyl)ethyl]chromone or isomer |
| **128** | | | 4.737 | | 357.1327 | | –1.68 | | C_20_H_20_O_6_ | 221, 205 | I | OCH_3_, OCH_3_ | OH, OCH_3_ | 6,7-dimethoxy-2-[2-(4′-hydroxy-3′-methoxyphenyl) ethyl]chromone or isomer |
| **129** | | | 6.460 | | 601.2015 | | –8.82 | | C_34_H_32_O_10_ | 583, 529, 301, 283, 227 | B |  |  | bi-phenylethylchromones II or isomer |
| **130** | | | 6.723 | | 583.1969 | | 1.03 | | C_34_H_30_O_9_ | 565, 547, 519, 373, 355, 301, 283 | B |  |  | AH_15_ or isomer |
| **131** | | | 6.882 | | 601.2027 | | –6.82 | | C_34_H_32_O_10_ | 583, 301, 283, 255, 227 | B |  |  | bi-phenylethylchromones II or isomer |
| **132** | | | 7.293 | | 327.1236 | | 2.75 | | C_19_H_18_O_5_ | 221, 205 | I | OCH_3_, OCH_3_ | OH, | 6,7-dimethoxy-2-[2-(4-hydroxyphenyl) ethyl]chromone or isomer |
| **133** | | | 7.483 | | 267.1016 | | 0 | | C_17_H_14_O_3_ | 161, 107 | I | – | OH | 2-[2-(3-hydroxyphenyl)ethyl]chromone or isomer |
| **134** | | | 7.725 | | 297.1095 | | –8.75 | | C_18_H_16_O_4_ | 206, 191, 167 | I | OH, OCH_3_ | – | 6-hydroxy-7-methoxy-2-(2-phenylethyl)chromone or isomer |
| **135** | | | 8.350 | | 583.2065 | | 9.09 | | C_34_H_30_O_9_ | 565, 547, 529, 301, 283*，255, 121 | E |  |  | Aquisinenone K or isomer |
| **136** | | | 8.972 | | 613.2118 | | 8.15 | | C_35_H_32_O_10_ | 595, 576, 446, 313, 121 | B |  |  | methoxy AH_15_ or isomer |
| **137** | | | 9.225 | | 311.1266 | | –3.86 | | C_19_H_18_O_4_ | 151 | I | OCH_3_ | OCH_3_ | 6-methoxy-2-[2-(3-methoxyphenyl)ethyl]chromone |
| **138** | | | 9.383 | | 601.2153 | | 14.14 | | C_34_H_32_O_10_ | 583, 565, 301, 283, 255, 227 | B |  |  | bi-phenylethylchromones II or isomer |
| **139** | | | 9.792 | | 297.1106 | | –5.05 | | C_18_H_16_O_4_ | 121 | I | OH | OCH_3_ | 6-hydroxy-2-[2-(4′-methoxyphenyl)ethyl]chromone or isomer |
| **140** | | | 10.077 | | 319.1192 | | 5.01 | | C_17_H_18_O_6_ | 301, 283, 255, 227 | II | – | – | 5,6,7,8-tetrahydroxy-2-(2-phenylethyl)-5,6,7,8-tetrahydrochromone or isomer |
| **141** | | | 10.140 | | 583.1960 | | –0.51 | | C_34_H_30_O_9_ | 565, 547, 283*, 255, 192, 91 | E |  |  | aquisinenone K or isomer |
| **142** | | | 10.453 | | 283.0939 | | –9.18 | | C_17_H_14_O_4_ | 192, 153 | I | OH, OH | – | 6,8-dihydroxy-2-(2-phenylethyl)chromone or isomer |
| **143** | | | 10.485 | | 583.1923 | | –6.86 | | C_34_H_30_O_9_ | 565, 547, 529, 474, 373, 283 | B |  |  | AH_15_ or isomer |
| **144** | | | 10.548 | | 327.1224 | | –0.92 | | C_19_H_18_O_5_ | 137 | I | OCH_3_ | OH, OCH_3_ | 6-methoxy-2-[2-(3-methoxy-4-hydroxyphenyl)ethyl]chromone or isomer |
| **145** | | | 10.770 | | 583.1931 | | –5.49 | | C_34_H_30_O_9_ | 565, 547, 474, 373, 283 | B |  |  | AH_15_ or isomer |
| **146** | | | 10.897 | | 643.2176 | | 0.31 | | C_36_H_34_O_11_ | 607, 283*, 227 | E |  |  | 4′,4‴-dimethoxyaquisinenone K or isomer |
| **147** | | | 10.897 | | 311.1249 | | –9.32 | | C_19_H_18_O_4_ | 220, 205, 181, 121, | I | OCH_3_, OCH_3_ | – | 6,7-dimethoxy-2-(2-(phenylethyl)chromone or isomer |
| **148** | | | 11.352 | | 341.1358 | | –7.62 | | C_20_H_20_O_5_ | 220, 105, 181, 121 | I | OCH_3_, OCH_3_ | OCH_3_ | 6,7-dimethoxy-2-[2-(4-methoxyphenyl)ethyl]chromone or isomer |
| **149** | | | 11.623 | | 565.1818 | | –6.90 | | C_34_H_28_O_8_ | 547, 529, 458*, 368, 267 | F |  |  | hydroxy aquisinenone A or isomer |
| **150** | | | 11.928 | | 565.1830 | | –4.78 | | C_34_H_28_O_8_ | 529, 474, 371, 283, 192 | F |  |  | 6′′-hydroxyaquisinenone B or isomer |
| **151** | | | 12.080 | | 595.1951 | | –2.02 | | C_35_H_30_O_9_ | 577, 458*, 423, 293, 266 | F |  |  | hydroxy 4′-methoxyaquisinenone A or isomer |
| **152** | | | 12.263 | | 625.1993 | | –12.00 | | C_36_H_32_O_10_ | 607, 487, 451, 423, 368, 332, 267 | F |  |  | methoxy and hydroxy 4′-methoxyaquisinenone A or isomer |
| **153** | | | 12.295 | | 595.1907 | | –9.41 | | C_35_H_30_O_9_ | 577, 559, 458, 313, 283, 255, 121 | D |  |  | 4′-methoxy-aquisinenone G or isomer |
| **154** | | | 12.358 | | 565.1828 | | –5.13 | | C_34_H_28_O_8_ | 547, 474, 283*, 107 | D |  |  | aquisinenone G or isomer |
| **155** | | | 12.450 | | 341.1371 | | –3.81 | | C_20_H_20_O_5_ | 220, 121 | I | OCH_3_, OCH_3_ | OCH_3_ | 6,7-dimethoxy-2-[2-(4-methoxyphenyl)ethyl]chromone or isomer |
| **156** | | | 12.510 | | 595.1920 | | –7.22 | | C_35_H_30_O_9_ | 577, 487, 458, 423, 357, 279, 266 | F |  |  | hydroxy 4′-methoxyaquisinenone A or isomer |
| **157** | | | 12.695 | | 565.1774 | | –14.69 | | C_34_H_28_O_8_ | 547, 529, 474, 373, 283*, 192 | D |  |  | aquisinenone G or isomer |
| **158** | | | 13.020 | | 549.1843 | | –11.84 | | C_34_H_28_O_7_ | 531, 513, 458, 423, 357, 283, 267 | F |  |  | aquisinenone A or isomer |
| **159** | | | 13.203 | | 565.1846 | | –1.96 | | C_34_H_28_O_8_ | 547, 529, 474, 438, 371, 283, 192 | F |  |  | 6′′-hydroxyaquisinenone B or isomer |
| **160** | | | 13.293 | | 619.1604 | | 0.81 | | C_36_H_36_O_10_ | 601, 319, 283, 227 | B |  |  | bi-phenylethylchromones II or isomer |
| **161** | | | 13.897 | | 639.2311 | | 13.45 | | C_37_H_34_O_10_ | 621, 593, 357*, 221, 137 | E |  |  | 6′′-methoxy-aquisinenone J or isomer |
| **162** | | | 14.018 | | 583.1971 | | 1.54 | | C_34_H_30_O_9_ | 565, 547, 301, 283*, 192, | E |  |  | aquisinenone K or isomer |
| **163** | | | 15.227 | | 865.2700 | | –17.91 | | C_51_H_44_O_13_ | 847, 829, 811, 565, 547, 474, 283 |  |  |  | tri-2-(2-phenylethyl)chromone or isomer |
| **164** | | | 18.083 | | 531.1730 | | –13.55 | | C_34_H_26_O_6_ | 513, 453, 440, 401, 373, 267 | A |  |  | AH_11_ or isomer |
| **165** | | | 18.113 | | 595.1894 | | –11.59 | | C_35_H_30_O_9_ | 577, 313*, 283, 267, 121 | D |  |  | 4′-methoxy-aquisinenone G or isomer |
| **166** | | | 18.143 | | 565.1792 | | –11.50 | | C_34_H_28_O_8_ | 547, 283*, 255, 192 | D |  |  | aquisinenone G or isomer |
| **167** | | | 19.047 | | 561.1908 | | 0 | | C_35_H_28_O_7_ | 484, 470, 431, 303, 279 | A |  |  | methoxy AH_11_ or isomer |
| **168** | | | 20.203 | | 865.2726 | | –14.91 | | C_51_H_44_O_13_ | 848, 829, 584, 565, 529, 283, 255, 192 |  |  |  | tri-2-(2-phenylethyl)chromone or isomer |
| **Fr.E** | | |  | |  | |  | |  |  |  |  |  |  |
| **169** | | | 1.312 | | 319.1160 | | –5.01 | | C_17_H_18_O_6_ | 301, 283, 255, 227 | II | – | – | 5,6,7,8-tetrahydroxy-2-(2-phenylethyl)-5,6,7,8-tetrahydrochromone or isomer |
| **170** | | | 1.342 | | 349.1273 | | –2.58 | | C_18_H_20_O_7_ | 331, 313, 285, 121 | II | – | OCH_3_ | 5,6,7,8-tetrahydroxy-2-[2-(4′-methoxyphenyl)ethyl] -5,6,7,8-tetrahydrochromone or isomer |
| **171** | | | 2.228 | | 283.0956 | | –3.18 | | C_17_H_14_O_4_ | 225, 227, 192 | III | – | – | 5,6:7,8-diepoxy-2-(2-phenylethyl)-5,6,7,8-tetrahydrochromone or isomer |
| **172** | | | 2.387 | | 337.0812 | | –7.42 | | C_17_H_17_O_5_Cl | 319, 301, 265, 255, 237 | II | – | – | 8-chloro-5,6,7-trihydroxy-2-(2-phenylethyl)-5,6,7,8-tetrahydrochromone or isomer |
| **173** | | | 6.807 | | 327.1220 | | –2.14 | | C_19_H_18_O_5_ | 221, 181, 205 | I | OCH_3_, OCH_3_ | OH | 6,7-dimethoxy-2-[2-(4-hydroxyphenyl)ethyl]chromone or isomer |
| **174** | | | 7.370 | | 583.2039 | | 13.03 | | C_34_H_30_O_9_ | 565, 547, 415, 283, 192 | B |  |  | AH_15_ or isomer |
| **175** | | | 9.335 | | 597.2094 | | –4.19 | | C_35_H_32_O_9_ | 565, 547, 297, 283, 206, 91 | B |  |  | 5-methoxy-AH13 isomer |
| **176** | | | 9.767 | | 565.1850 | | –1.24 | | C_34_H_28_O_8_ | 547, 459, 441, 368, 267 | F |  |  | hydroxy aquisinenone A or isomer |
| **177** | | | 10.108 | | 597.2119 | | –3.01 | | C_35_H_32_O_9_ | 579, 561, 474, 297, 283, 206, 191 | B |  |  | AH_12_ or isomer |
| **178** | | | 10.500 | | 595.1971 | | 1.34 | | C_35_H_30_O_9_ | 577, 503, 474, 439, 373, 319, 283, | F |  |  | methoxy 6′′-hydroxyaquisinenone B or isomer |
| **179** | | | 10.580 | | 281.1151 | | –7.47 | | C_18_H_16_O_3_ | 121 | I | – | OCH_3_ | 2-(2-4′-methoxyphenylethyl)chromone or isomer |
| **180** | | | 13.440 | | 565.1768 | | –15.75 | | C_34_H_28_O_8_ | 547, 529, 474, 439, 283, 193 | F |  |  | 6′′-hydroxyaquisinenone B or isomer |
|  | | |  | |  | |  | |  |  |  |  |  |  |
| **Fr.F** | | | | | | | | | | |  |  |  |  |
| **181** | | | 4.218 | | 613.1964 | | –16.96 | | C_35_H_32_O_10_ | 595, 577, 475, 313*, 283, 267 | E |  |  | methoxy aquisinenone K or isomer |
| **182** | | | 4.313 | | 583.1980 | | 2.91 | | C_34_H_30_O_9_ | 565, 547, 301, 283*, 192 | E |  |  | aquisinenone K or isomer |
| **183** | | | 6.822 | | 311.1234 | | –14.14 | | C_19_H_18_O_4_ | 220, 205, 91 | I | OCH_3_, OCH_3_ | – | 6,7-dimethoxy-2-(2-(phenylethyl)chromone or isomer |
| **184** | | | 7.202 | | 565.1945 | | 15.57 | | C_34_H_28_O_8_ | 547, 474, 283, 192 | D |  |  | aquisinenone G or isomer |
| **185** | | | 7.328 | | 595.1993 | | 5.04 | | C_35_H_30_O_9_ | 577, 559, 531, 435, 313, 121 | D |  |  | 4′-methoxy-aquisinenone G or isomer |
| **186** | | | 7.863 | | 565.1909 | | 9.20 | | C_34_H_28_O_8_ | 547, 529, 474, 283,192 | C |  |  | AH_21_ or isomer |
| **187** | | | 8.763 | | 579.1941 | | –12.43 | | C_35_H_30_O_8_ | 561,543, 488, 453, 359, 266 | F |  |  | 6′′-methoxy-aquisinenone B or isomer |
| **188** | | | 9.785 | | 579.1957 | | –9.67 | | C_35_H_30_O_8_ | 561, 543, 458, 357, 303, 266 | F |  |  | 4′-methoxy-aquisinenone A or isomer |
| **189** | | | 10.615 | | 549.1857 | | –9.29 | | C_34_H_28_O_7_ | 531, 513, 458, 423, 329, 267 | F |  |  | aquisinenone A or isomer |
| **190** | | | 12.485 | | 301.1052 | | –6.31 | | C_17_H_16_O_5_ | 283, 255, 227, 164 | IV | – | – | rel-(1a*R*,2*R*,3*R*,7b*S*)-1a,2,3,7b-Tetrahydro-2,3-dihydroxy-5-(2-phenylethyl)-7H-oxireno[f][1]benzopyran-7-one or isomer |

**Structural elucidation of PECs by LC/MS**

**Identification of FTPECs (I) in Agarwood**

Compounds **18**, **60**, **63**, **91**, and **142** produced the same [M+H]^+^ ions at *m/z* 283, corresponding to the molecular formula of C_17_H_14_O_4_, with 11 indices of hydrogen deficiency. They produced the same fragment ion at *m/z*192 [^6^A+OH×2]^+^ and *m/z* 153 [^1,3^A+OH×2]^+^ , indicating that they contained two hydroxy groups in A ring. Thus, compounds **18**, **60**, **63**, **91**, and **142** were tentatively identified as 6,8-dihydroxy-2-(2-phenylethyl)chromone [1] or isomer. Compound **59 (**C_17_H_14_O_4_**)** generated the fragment ion at *m/z* 177 [^6^A+OH]^+^, *m/z* 107 [^6^B+OH]^+^, and *m/z* 137 [^1,3^A+OH]^+^, revealing the distributions of one hydroxyl substitute at A- and B-ring, respectively; hence, **59** was plausibly identified as 6-hydroxy-2-[2-(4-hydroxyphenyl)ethyl]chromone[1]. In a similar manner, compounds **20**, **36**, **69**, and **96** were tentatively identified as 6-hydroxy-2-(2-phenylethyl)chromone or isomer[2], compounds **93** and **133** were tentatively identified as 2-[2-(3-hydroxyphenyl)ethyl]chromone or isomer[2]. Compound **31** generated the protonated molecular ion at *m/z* 299 [M+H]^+^ and the fragment ion at *m/z* 193 [^6^A+OH×2]^+^ and *m/z* 107 [^6^B+OH]^+^, indicating the presence of two hydroxy substitutes at A-ring and one hydroxy group at B-ring; hence, **31** was plausibly identified as 5,7-dihydroxy-2-[2-(4-hydroxyphenyl)ethyl]chromone[3]. The molecular formula of compound **137** was predicted as C_19_H_18_O_4_, the same as **38**, **79**, **104**, **147**, and **183**. However, the fragment ion of **137** was observed at *m/z* 151 [^1,3^A+ OCH_3_]^+^, indicating a single methoxy substitution occurred at A-ring, whereas compounds **38**, **79**, **104**, **147**, and **183** exhibited fragment ions at *m/z* 220 [^6^A+OCH_3_×2]^+^ and *m/z* 205 [^6^A+OCH_3_×2–·CH_3_]^+^. Thus, compound **137** was tentatively assigned as 6-methoxy-2-[2-(3-methoxyphenyl)ethyl]chromone[4], compounds **38**, **79**, **104**, **147**, and **183** were tentatively identified as 6,7-dimethoxy-2-(2-phenylethyl)chromone [4] or isomer. In the similar way, the structure of compound **40** was reasonably deduced as 6-methoxy-2-(2-phenylethyl)chromone[4], compound **179** was tentatively identified as 2-(2-4′-methoxyphenylethyl) chromone[5]. \. Analogous mass spectral profiles were exhibited for compounds **58** and **64**, including the protonated molecular ion at *m/z* 313 ([M+H]^+^) and the product ions at *m/z* 192 [^6^A+OH×2]^+^ and *m/z* 121 [^6^B+OCH_3_]^+^; hence, the structure of **58** and **64** were tentatively elucidated as 6,7-dihydroxy-2-[2-(4′- methoxyphenyl)ethyl]chromone[6]. In addition, the detection of the fragment ion at *m/z* 177 [^6^A+OH]^+^ and *m/z* 137 [^6^B+OCH_3_+OH]^+^ for compound **61**, the other isomer of **58**, indicated that the structure of **61** should be 6,3′-dihydroxy-4′-methoxy-2-(2-phenylethyl)chromone[6]. The same molecular formula of compounds **65**, **127**, **132**, **173, 94**, **144**, and **98** was determined as C_19_H_18_O_5_, however, they exhibited different product ions. Compounds **65, 127**, **132,** and **173** produced the same fragment ion at *m/z* 221 [^6^A+OCH_3_×2]^+^, indicating that they contained two methoxy substitutions in A ring. Thus, **65, 127**, **132**, and **173** were tentatively identified as 6,7-dimethoxy-2-[2-(4-hydroxyphenyl)ethyl]chromone[7] or isomer. The fragment ions at *m/z* 191 [^6^A+OCH_3_]^+^ and *m/z* 176 [^1,3^B+OCH_3_+OH]^+^were observed for **94**, and the fragment ion at *m/z* 137 [^6^B+OCH_3_+OH]^+^ was observed for **144,** indicating the distributions of one hydroxy and one methoxy substitutes at B-ring; hence, compounds **94** and **144** were tentatively identified as 6-methoxy-2-[2-(3-methoxy-4-hydroxyphenyl)ethyl]chromone[1] or isomer. Compound **98**, with the fragment ions at *m/z* 206 [^6^A+OCH_3_+OH]^+^ and *m/z* 121 [^6^B+OCH_3_]^+^, was tentatively identified as 7-hydroxy-6-methoxy-2-[2-(4-methoxyphenyl)ethyl]-4*H*-1-benzopyran-4-one[8]. The molecular weight of both **66** and **128** was 356 Da, 30 Da (OCH_3_) more than **65**. Compound **66** yielded the fragment ions at *m/z* 221 [^6^A+OCH_3_×2]^+^ and *m/z* 137 [^6^B+OCH_3_+OH]^+^, indicating that two methoxy groups occurred in A-ring, one hydroxy and one methoxy group in B-ring, and it was tentatively identified as 6,7-dimethoxy-2-[2-(3′-hydroxy-4′-methoxyphenyl)ethyl]chromone[6]. Meanwhile, compound **128** was assigned as 6,7-dimethoxy-2-[2-(3′-hydroxy-4′-methoxyphenyl)ethyl]chromone isomer. Using similar strategies, compounds **74** and **139** were tentatively identified as 6-hydroxy-2-[2-(4′-methoxyphenyl)ethyl]chromone[9], compound **92** was assigned as 4′-hydroxy-6-methoxy-2-(2-phenyl)ethylchromone [10]. Meanwhile, compounds **68, 97** and **134** were tentatively identified as -hydroxy-7-methoxy-2-(2-phenylethyl)chromone [1] or isomer. The molecular weight of either **90** or **125** was 342 Da. Compound **90** generated fragment ions at *m/z* 207 [^6^A+OCH_3_+OH]^+^ and *m/z* 137 [^6^B+OCH_3_+OH]^+^, indicating that one hydroxy and one methoxy occurred in both A- and B-ring. This data led to the identification of the compound **90** as 7-hydroxy-6-methoxy-2-[2-(3′-hydroxy-4′-methoxyphenyl)ethyl]chromone, as the mass fragmentation pattern was consistent with the information in the literature [6]. Simultaneously, compound **125** was identified as 7-hydroxy-6-methoxy-2-[2-(3′-hydroxy-4′-methoxyphenyl)ethyl]chromone isomer. The fragment ions at *m/z* 220 [^6^A+OCH_3_×2]^+^ and *m/z* 121 [^6^B+OCH_3_]^+^ were observed for **148** and **155**, respectively. In addition the fragment ion at *m/z* 181 [^1,3^A+OCH_3_×2]^+^ was observed for **148**, indicating the distributions of one methoxy substitute at A-ring and two methoxy substitutes at B-ring; hence, compounds **148** and **155** were tentatively identified as 6,7-dimethoxy-2-[2-(4-methoxyphenyl) ethyl]chromone [11] or isomer. Detailed information can be found in Table S1.

**Identification of** **THPECs (II) in Agarwood**

Compounds **1**, **2**, **5**, **7**, **43**, **44, 86**, **118**, **140**, and **169** were classified into THPECs on the basis of their characteristic MS fragmentation behaviors. These compounds produced the same protonated molecular ion at *m/z* 319 ([M+H]^+^) and fragment ion at *m/z* 301 [M+H–H_2_O]^+^ and *m/z* 255 [M+H–2H_2_O–CO]^+^, suggesting that they were isomer and displayed the similar MS fragmentation behaviors as agarotetrol [12]. Thus, Compounds **1**, **2**, **5**, **7**, **43**, **44, 86**, **118**, **140**, and **169** were tentatively identified as 5,6,7,8-tetrahydroxy-2-[2-(4′methoxyphenyl)ethyl]-5,6,7,8-tetrahydrochromone or isomer. In the similar manner, the structure of compounds **6**, **9**, **11**, **46**, **84**, **119**, and **170** were plausibly established as the isomer of 5,6,7,8-tetrahydroxy-2-[2-(4′-methoxyphenyl)ethyl]-5,6,7,8-tetrahydrochromone. Compound **41**, with a [M+H]^+^ ion at *m/z* 349 (C_18_H_20_O_8_), was tentatively identified as 5,6,7,8-tetrahydroxy-2-[2-(3′-hydroxy-4′-methoxyphenyl)ethyl]-5,6,7,8-tetrahydrochromone according to its accurate masses and the reported data [10]. Compound **42** was identified as aquilarone F [10]. Compounds **8**, **14**, **21**, and **28** exhibited comparable mass spectral patterns, featuring the neutral cleavages of MeOH and H_2_O; hence, the structure of compounds **8**, **14**, and **21** were tentatively elucidated as 5,6,7-trihydroxy-8-methoxy-5,6,7,8-tetrahydro-2-(2-phenylethyl) chromone [13]. Conversely, the detection of the fragment ion at *m/z* 121 [^6^B+OCH_3_]^+^ in the positive ionization mode for compound **28**, indicated that the structure of **28** should be 5,6,7-trihydroxy-8-methoxy-5,6,7,8-tetrahydro-2-[2-(4′-methoxyphenyl)ethyl]chromone.

Compounds **25**, **29**, **85**, **120**, **172**, and **32** were assigned to chlorinated THPECs, with their identity supported by the approximate 3:1 intensity ratio of the ions [M+H]^+^/[M+2H]^+^ and the neutral cleavages of HCl. The molecular formula of compounds **25**, **85**, **120**, and **172** was predicted as C_17_H_17_ClO_5_, and they exhibited similar fragmentation patterns, featuring the fragment ion at *m/z* 319 [M+H–H_2_O]^+^, *m/z* 301 [M+H–2H_2_O]^+^, and *m/z* 265 [M+H–2H_2_O–HCl]^+^. Consequently, **25**, **85**, **120**, and **172** were identified as 8-chloro-5,6,7-trihydroxy-2-(2-phenylethyl)-5,6,7,8-tetrahydrochromone isomers [14]. Given 30 Da greater than compound **25**, compound **32** was tentatively assigned as rel-(5*R*,6*S*,7*S*,8*R*)-8-chloro-5,6,7,8-tetrahydro-5,6,7-trihydroxy-2-[2-(4-methoxyphenyl)ethyl]-4H-1-benzopyran-4-one[8].

The molecular formula of compounds **13**, **19**, **22**, **45**, and **47** was predicted as C_17_H_18_O_5_, and they exhibited similar fragmentation patterns, featuring the fragment ion at *m/z* 285 and *m/z* 267 resulting from the successive neutral loss of two molecules of H_2_O·and the *m/z* 239 corresponding to the subsequent CO neutral loss. Thus, compounds **13**, **19**, **22**, **45**, and **47** was tentatively identified as 5,6,7-trihydroxy-2-(2-phenylethyl)-5,6,7,8-tetrahydrochromone isomers, which has been isolated from Chinese agarwood [8]. Compounds **15**, **17**, **50**, and **23** were tentatively identified as 5,6,7-trihydroxy-2-[2-(4′-methoxyphenyl)ethyl]-5,6,7,8-tetrahydrochromone isomers in a similar way. Compound **24** and **30** produced the protonated molecular ion at *m/z* 287 [M+H]^+^ and the fragment ion at *m/z* 269 [M+H–H_2_O]^+^, *m/z* 251 [M+H–2H_2_O]^+^, and *m/z* 160 [^6^A]^+^ suggesting the presence of two hydroxy group at A-ring; hence, compound **24** and **30** was plausibly identified as 6,7-cis-dihydroxy-2-(2-phenylethyl)-5,6,7,8-tetrahydrochromone isomers[14]. Compounds **27** and **33** were assigned as 6,7-*cis*-dihydroxy-5,6,7,8-tetrahydro-2-(2-(4-methoxyphenyl)ethyl)chromone [15] isomers owing to the signals at *m/z* 299 [M+H–H_2_O]^+^, *m/z* 281 [M+H–2H_2_O]^+^, and *m/z* 121 [^6^B+OCH_3_]^+^ in their HR-MS/MS spectra.

**Identification of B type PEC dimers in Agarwood**

Compounds **37**, **78**, and **108** have the same molecular weight of 566 Da, with the molecular formula C_34_H_30_O_8_. Compound **37** produced fragment ions at *m/z* 549 ([M+H–H_2_O]^+^), 531 ([M+H–2H_2_O]^+^), 513 ([M+H–2H_2_O–CO]^+^), 283 ([M_unit A_+H–2H_2_O]^+^), and 267 ([M_unit B_+H). These fragmentation patterns precisely aligned with the literature description, allowing for the identification of compound 37 as 2-phenethyl-6-(((5*S*,6*R*,7*R*,8*S*)-5,6,7-trihydroxy-4-oxo-2-phenethyl-5,6,7,8-tetrahydro-4*H*-chromen-8-yl)oxy)chromone (AH_13_) [16]. Meanwhile, compounds **78** and **108** was assigned as AH_13_ isomer. Compounds **107**, **111**, and, **177** were tentatively identified as AH_12_[17] isomers in a similar way. The molecular formula of compound **102** was predicted as C_35_H_32_O_9_, and the fragment ions at *m/z* 565 ([M+H–MeOH]^+^), 547 ([M+H–MeOH–H_2_O]^+^), and 283 ([M_unit A_+H–MeOH–H_2_O]^+^) were observed for **102**. Therefore, compound **102** was tentatively identified as 5-methoxy-AH_13_ isomer. The molecular formula of compounds **56**, **67**, **95**, **130**, **143**, **145**, and **174** was predicted as C_34_H_30_O_9_, all of which exhibited identical fragmentation pathways. Notably, the fragment ion at *m/z* 565, 547, and *m/z* 519 resulting from the successive neutral loss of two molecules of H_2_O and one·molecule of CO, while the fragment ion at *m/z* 283 indicated the cleavage of carbon-carbon bonds linking the two constituent monomers. therefore, compounds **56**, **67**, **95**, **130**, **143**, **145**, and **174** was tentatively identified as AH_15_ isomers. Similarly, three isomers, **73**, **75**, and **136** (C_35_H_32_O_10_) were putatively assigned as methoxyAH_15_. Moreover, compounds **129**, **131**, and **138** were also assigned as B type dimer comprising THPEC and a EPEC. Detailed information can be found in Table S1. It is worth mentioning that compounds **129**, **131**, and **138** were reported for the first time.

**Identification of D type PEC dimers in Agarwood**

The same molecular formula, C_34_H_28_O_8_, was predicted for both compounds **77** and aquisinenone G [18], and they exhibited similar fragmentation pathways, including the fragment ion at *m/z* 547 ([M+H−H_2_O]^+^) and *m/z* 529 ([M+H−2H_2_O]^+^) and the MS^2^ base peak ion at *m/z* 283 corresponding to the breakage of the carbon-carbon bonds between two monomers. The monomer-derived fragment ion (*m/z* 283) could also break the CH_2_–CH_2_ bond between chromone moiety and phenyl moiety to produce [M_unit A_+H−^6^B]^+^ (*m/z* 192) ion. Therefore, compound **77** was tentatively identified as aquisinenone G isomers. Through the comprehensive analysis of MS/MS spectra, compounds **62**, **116**, **154**, **157**, **166**, and **184** was also designated as aquisinenone G isomer. Detailed information was elaborated in Table S1.

**Identification of E type PEC dimers in Agarwood**

Compounds **72**, **76**, **135**, **162**, and **182** exhibited the molecular formula C_34_H_30_O_9_ according to the protonated molecular ion at *m/z* 583 [M+H]^+^, suggesting 20 indices of hydrogen deficiency. A series of characteristic ions such as *m/z* 565 [M+H–H_2_O]^+^, *m/z* 547 [M+H–2H_2_O]^+^, *m/z* 519 [M+H–2H_2_O–CO]^+^, and *m/z* 283 produced by breaking the ester bonds between two monomers were detected in the mass spectra of these compounds. Among them, fragment ion *m/z* 283 is the base peak in MS^2^, and its cleavage pathway is the same as that of aquisinenone K. Therefore, compounds **72**, **76**, **135**, **162**, and **182** were identified as isomers of aquisinenone K [19]. Analogously, a pair of compounds, **35** and **181** (C_35_H_32_O_10_) were tentatively classified as methoxy aquisinenone K. Furthermore, compound **146** was provisionally identified as 4′,4‴-dimethoxyaquisinenone K.

**Identification of F type PEC dimers in Agarwood**

Compounds **158** and **189** exhibited similar mass spectral profiles, featuring a precursor ion at *m/z* 549 ([M+H]^+^), and product ions at *m/z* 458 ([M+H–^6^B]^+^), *m/z* 531([M+H–H_2_O]^+^), *m/z* 513([M+H–2H_2_O]^+^), *m/z* 423 ([M+H–2H_2_O–^6^B]^+^), *m/z* 267 ([M_unit B_+H]^+^), and *m/z* 283 ([M_unit A_+H–2H_2_O]^+^); the structure of compounds **158** and **189** were tentatively elucidated as aquisinenone A [18]. Utilizing comparable methods, compounds **117** and **188** were assigned as 4′-methoxyaquisinenone A isomers. Compounds **149** and **176** (C_34_H_28_O_8_) exhibited one more hydroxyl than aquisinenone A (C_34_H_28_O_7_), and the fragment ions were observed at *m/z* 547 [M+H–H_2_O]^+^, *m/z* 529 [M+H–2H_2_O]^+^, 458 ([M+H–^6^B]^+^), and *m/z* 267 ions. The monomer-derived fragment ions at *m/z* 267 ([M_unit B_+H]^+^) indicated the presence of an additional hydroxyl group at B-ring. Therefore, the structure of compounds **149** and **176** were tentatively elucidated as hydroxy aquisinenone A. Similarly, a pair of isomers, **151** and **156** (C_35_H_30_O_9_) were tentatively characterized as hydroxy 4′-methoxyaquisinenone A, and compound **152** was putatively classied as methoxy and hydroxy 4′-methoxyaquisinenone A. Compounds **80**, **110**, and **178** had a molecular formula of C_35_H_30_O_9_, the same as **151** and **156**, and exhibited fragment ions at *m/z* 577 [M+H–H_2_O]^+^, 474 ([M+H–^6^B]^+^), and *m/z* 283 ions. Thus, compounds **80**, **110**, and **178** were tentatively identified as methoxy 6′′-hydroxyaquisinenone B or isomer.

Compounds **99**, **105**, **109**, **150**, **159**, and **180** exhibited the molecular formula C_34_H_28_O_8_ according to the protonated molecular ion at *m/z* 565 [M+H]^+^, indicating 21 indices of hydrogen deficiency. A series of characteristic ions such as *m/z* 547 [M+H–H_2_O]^+^, *m/z* 531 [M+H–2H_2_O]^+^, *m/z* 474 [M+H–^6^B]^+^, and *m/z* 283 produced by the HRF-initiated synergetic cleavage of the linkages of units A and B were detected in the mass spectra of these compounds. Among them, fragment ion *m/z* 474 is the base peak in MS^2^, and its cleavage pathway is the same as that of 6′′-hydroxyaquisinenone B. Therefore, compounds **99**, **105**, **109**, **150**, **159**, and **180** were identified as isomers of 6′′-hydroxyaquisinenone B [18]. Similarly, compound **115** was preliminarily determined as aquisinenone C. And most remarkably, redshift (from to 250 nm) was observed in the UV spectrum of **99**, **105**, **109**, **150**, **159**, **180**, and **115** in comparison of the other dimers due to the presence of hydroxyl groups at C-6′′.

***Purification of new PEC dimers***

The dried Chinese agarwood sample (10.0 kg) was extracted with 95% EtOH (3 × 120 L, each 2.0 h) under reflux. Following the removal of the solvent under reduced pressure, the EtOH extract was suspended in 80% aqueous MeOH (1 L). Subsequently, petroleum ether (80.9 g) and EtOAc (1901.5 g) soluble extracts were obtained by successive extraction with petroleum ether and EtOAc. The EtOAc extract (1901.5 g) was applied to silica gel (200–300 mesh) vacuum liquid chromatography and eluted with CHCl_3_-MeOH (1: 0–0:1) to provide 18 fractions (Fr.A−Fr.R). Subsequently, LC-MS-guided isolation and purification targeted the subfractions of Fr.M including compounds with *m/z* > 500. Therefore, Fr.M (8.9 g) was isolated on silica gel column eluting with CHCl_3_-MeOH (1:0–0:1) to yield Fr.M1−Fr.M5. Fr.M3 (4.0 g) was subjected to an ODS gel column eluting with MeOH-H_2_O (*v*/*v*, 40:60–100:0) to give Fr.M3-1−Fr.M3-8. Fr.M3-3 (110.0 mg) was separated by semipreparative HPLC eluted with an isocratic 23% aqueous ACN to produce compound **VIII** (*t*_R_ 12.5 min 4.2 mg) and compound **VII** (*t*_R_ 33.0 min; 6.0 mg). Fr.M3-5 (212.0 mg) was purified by semipreparative HPLC (ACN/H_2_O, 25:75, 3 mL/min) to give compound **V** (*t*_R_ 66.0 min; 1.3 mg); Fr.M3-6 (1.44 g) was purified on Sephadex LH-20 gel column eluting with CHCl_3_-MeOH (3:2) to give 5 fractions, namely Fr.M3-6-1−Fr.M3-6-5, Fr.M3-6-4 (937 mg) was followed by ODS gel column eluting with MeOH-H_2_O (50:50–100:0) to give Fr.M3-6-4-1−M3-6-4-6. Fr.M3-6-4-1 (25.0 mg) was purified by semipreparative HPLC (ACN/H_2_O, 25:75, 3.0 mL/min) to give compound **IV** (*t*_R_ 43.0 min; 1.6 mg), compound **IV** was further isolated using a chiral-phase HPLC (CHIRALPAKIBN-3 column) eluting with *n*-hexane−EtOH (60:40) to yield I**Va** (0.5 mg) and **IVb** (0.7 mg).; Fr.M3-6-4-4 (20 mg) was followed by semipreparative HPLC on an ODS column (ACN/H_2_O, 45:55, 3 mL/min) to give compound **II** (*t*_R_ 20.5 min; 3.3 mg), compound **I** (*t*_R_ 30.0 min; 2.4 mg), and compound **VI** (*t*_R_ 32.5 min; 1.6 mg), compound **II** was further separated using a chiral-phase HPLC (CHIRALPAKIBN-3 column) eluting with *n*-hexane−EtOH (60:40) to yield **IIa** (1.0 mg) and **IIb** (1.3 mg). Fr.M3-5 (500 mg) was separated by ODS gel column with MeOH/H_2_O (70:30–100:0) to afford 5 fractions (Fr.M3-5-1−M3-5-6). Fr.M3-5-5 (82.0 mg) was purified by semipreparative HPLC (ACN/H_2_O, 65:35, 3 mL/min) to give compounds **III** (*t*_R_ 32.5 min; 2.6 mg), Furthermore, compound **III** was analysed using a chiral-phase HPLC (CHIRALPAKIBN-3 column) eluting with *n*-hexane−EtOH (20:80) to yield **IIIa** (1.0 mg) and **IIIb** (1.1 mg).

Thereafter, extensively assays were conducted for structural identification of those obtained components. Optical rotations were obtained on a Rudolph Autopol IV automatic polarimeter (NJ, USA). The HRESIMS were acquired through an LCMS-IT-TOF system, which was seamlessly integrated a Prominence UFLC system and an ESI interface (Shimadzu, Kyoto, Japan). UV spectra were recorded on a Hitachi UH-5300 spectrophotometer (Hitachi, Japan). ECD spectra were measured on a J-810 CD spectrophotometer (JASCO, Japan). IR spectra were obtained using a Thermo Nicolet iS50 FT-IR spectrophotometer (Nicolet Company, USA). NMR spectra were measured with a Bruker AVANCE NEO 400 spectrometer, operating at 600 MHz and 400 MHz for ^1^H, 150 MHz and 100 MHz for ^13^C.

***Physicochemical Properties of New Compounds***

Aquisinenone P (**I**): colorless oil; [*α*]25 D–72 (*c* 0.1, MeOH); UV *λ*MeOH max(log *ε*): 206 (4.17) nm; ECD (*c* 0.001, MeOH): 216 (*Δε* +14.20), 242 (*Δε* –24.44), 295 (*Δε* +2.28); IR (film) *ν*_max_: 3344, 2967, 2926, 1656, 1632, 1602, 1478, 1453, 1375, 1286, 1190, 1076, 1044, 1007, 965, 748, 699, 556 cm^-1^; positive-ion HRESIMS *m/z* 567.2023 [M + H]^+^ (calcd for C_34_H_31_O_8_, 567.2013); ^1^H and ^13^C NMR data see Table 2 in the main text.

Aquisinenone Q racemic mixture (**II**): white amorphous powder; [*α*]25 D–8 (*c* 0.1, MeOH); UV *λ*MeOH max(log *ε*): 205 (4.30) nm; IR (film) *ν*_max_: 3342, 2929, 1659, 1636, 1601, 1512, 1451, 1374, 1269, 1246, 1193, 1076, 1035, 849, and 700 cm^-1^; negative-ion HRESIMS *m/z* 625.2093 [M – H]^–^ (calcd for C_36_H_33_O_10_, 625.2079); ^1^H and ^13^C NMR data see Table 2 in the main text.

(+)-Aquisinenone Q (**IIa**): [*α*]25 D+32 (*c* 0.1, MeOH); ECD (*c* 0.001, MeOH): 246 (*Δε* –23.32), 289 (*Δε* +1.22), 317 (*Δε* –5.37)

(–)-Aquisinenone Q (**IIb**): [*α*]25 D–36 (*c* 0.1, MeOH); ECD (*c* 0.001, MeOH): 246 (*Δε* +23.01), 288 (*Δε* –1.21), 317 (*Δε* +5.32)

7′′-Demethoxyaquisinenone Q Scalemic Mixture (**III**): colorless oil; [*α*]25 D–8 (*c* 0.1, MeOH); UV *λ*MeOH max(log *ε*): 209 (4.49) nm; IR (film) *ν*_max_: 3342, 2968, 2929, 1657, 1633, 1608, 1512, 1478, 1452, 1375, 1245, 1190, 1041, 966, 824, 700 cm^-1^; negative-ion HRESIMS *m/z* 595.1952 [M – H]^–^ (calcd for C_35_H_31_O_9_, 595.1974); ^1^H and ^13^C NMR data see Table 2 in the main text.

(+)-7′′-Demethoxyaquisinenone Q (**IIIa**): [*α*]25 D+32 (*c* 0.1, MeOH); ECD (*c* 0.001, MeOH): 214 (*Δε* –1.46), 243 (*Δε* +3.28), 293 (*Δε* –0.24)

(–)-7′′-Demethoxyaquisinenone Q (**IIIb**): [*α*]25 D–36 (*c* 0.1, MeOH); ECD (*c* 0.001, MeOH): 214 (*Δε* +1.46), 243 (*Δε* –3.28), 293 (*Δε* +0.24)

(±)-Aquisinenone R racemic mixture (**IV**): white amorphous powder; [*α*]25 D0 (*c* 0.1, MeOH); UV *λ*MeOH max(log *ε*): 207 (4.54), 230 (4.48) nm; IR (film) *ν*_max_: 3323, 2967, 1659, 1633, 1594, 1512, 1451, 1377, 1272, 1245, 1209, 1190, 1129, 1080, 1031, 993 cm^-1^; negative-ion HRESIMS *m/z* 671.2140 [M – H]^–^ (calcd for C_37_H_35_O_12_, 671.2134); ^1^H and ^13^C NMR data see Table 2 in the main text.

(+)-Aquisinenone R (**IVa**): [*α*]25 D+12 (*c* 0.1, MeOH); ECD (*c* 0.001, MeOH): 238 (*Δε* +1.23), 263 (*Δε* –0.35), 299 (*Δε* +0.51)

(–)-Aquisinenone R (**IVb**): [*α*]25 D–12 (*c* 0.1, MeOH); ECD (*c* 0.001, MeOH): 238 (*Δε* –1.22), 264 (*Δε* +0.35), 297 (*Δε* –0.50)

Aquisinenone S (**V**): white amorphous powder; [*α*]25 D–12 (*c* 0.1, MeOH); UV *λ*MeOH max(log *ε*): 206 (4.30), 243 (4.15) nm; ECD (*c* 0.001, MeOH): 220 (*Δε* +12.23), 244 (*Δε* –24.30), 275 (*Δε* +6.84); IR (film) *ν*_max_: 3315, 2971, 2928, 1656, 1600, 1454, 1381, 1087, 1046, 879, 699 cm^-1^; negative-ion HRESIMS *m/z* 581.1822 [M – H]^–^ (calcd for C_34_H_29_O_9_, 581.1817); ^1^H and ^13^C NMR data see Table 2 in the main text.

***MS, UV, IR, and NMR Spectra of New Compounds***


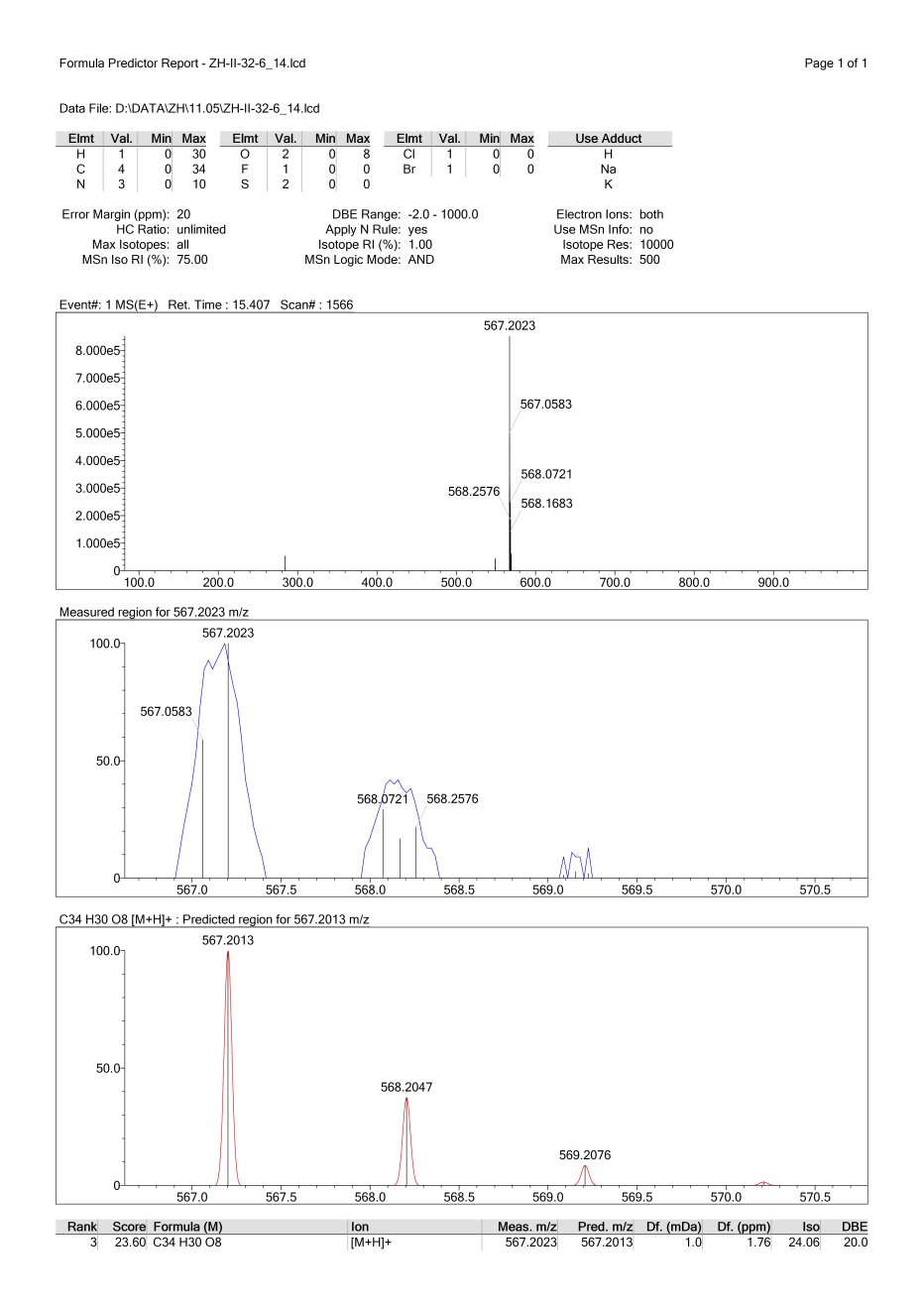


HRESIMS spectrum of compound **I**


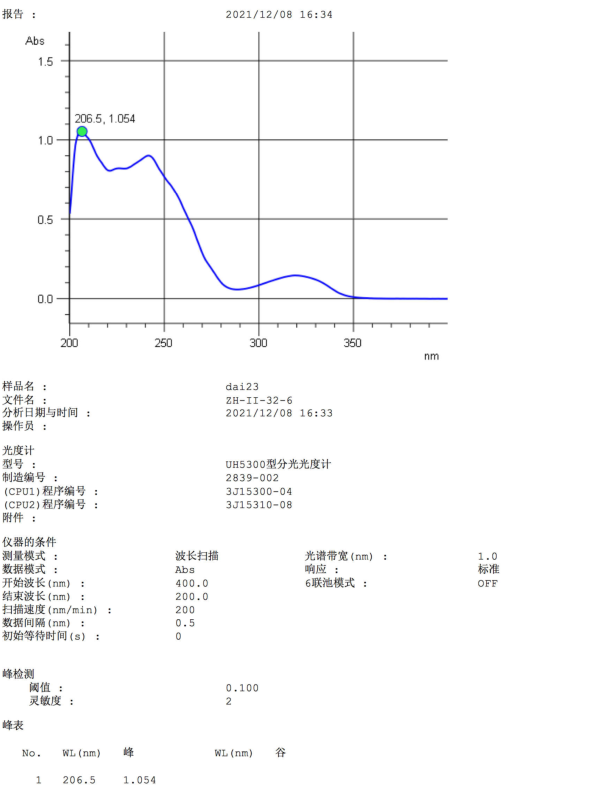


UV spectrum of compound **I**


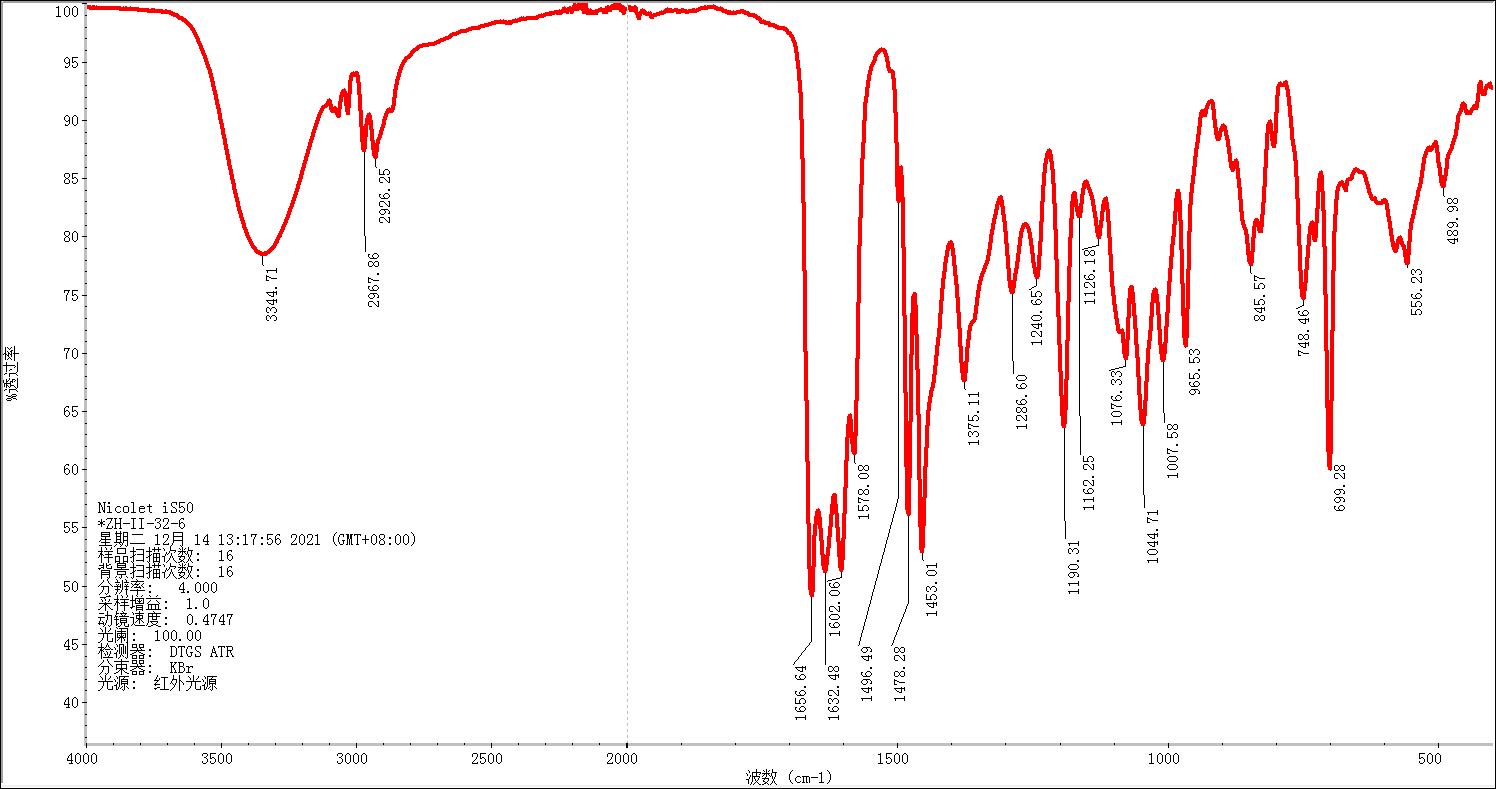


IR spectrum of compound **I**


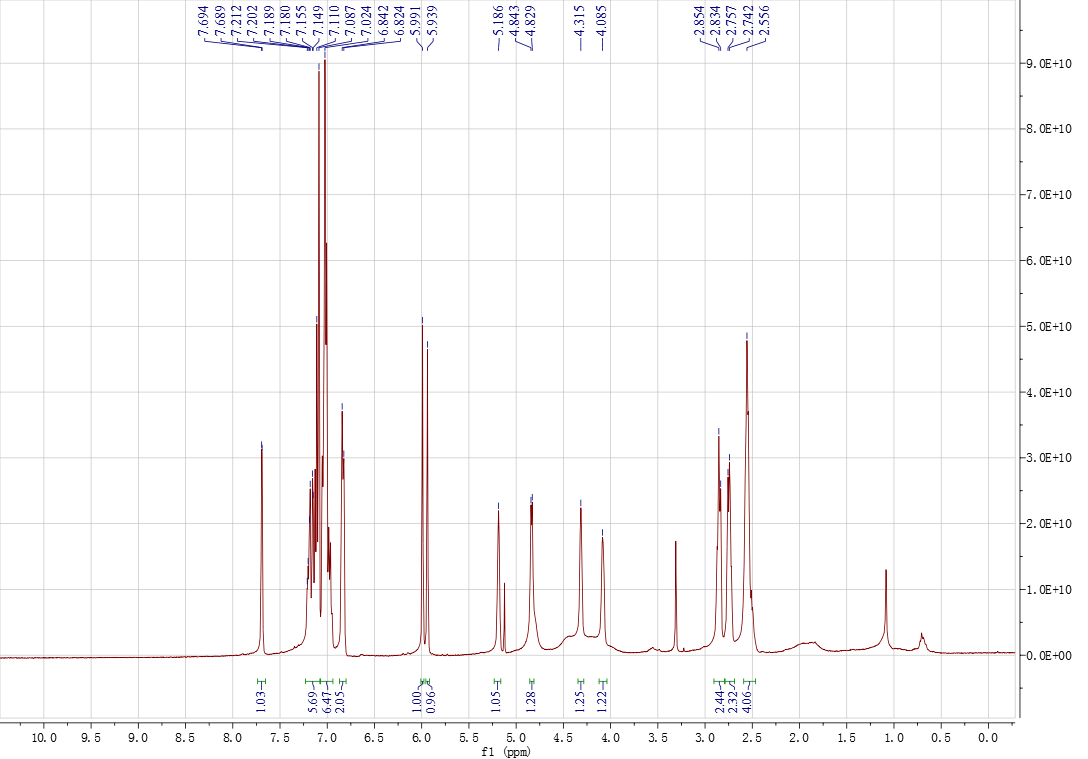


^1^H NMR spectrum of compound **I** in CDCl_3_


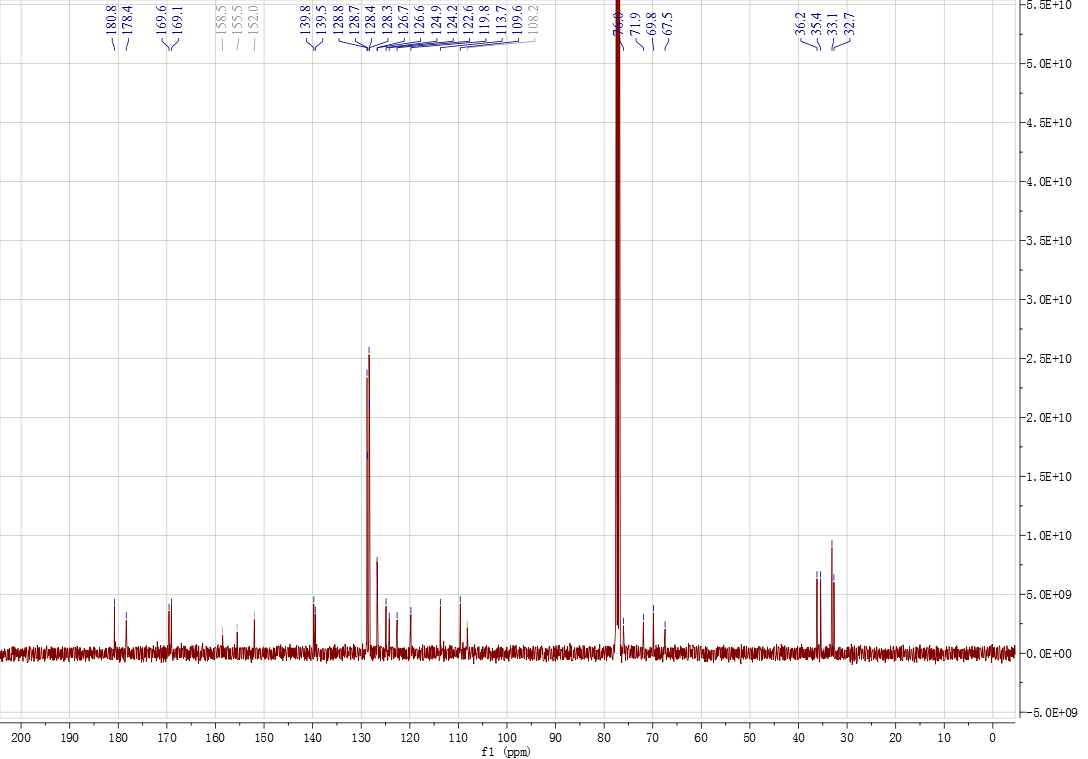


^13^C NMR spectrum of compound **I** in CDCl_3_


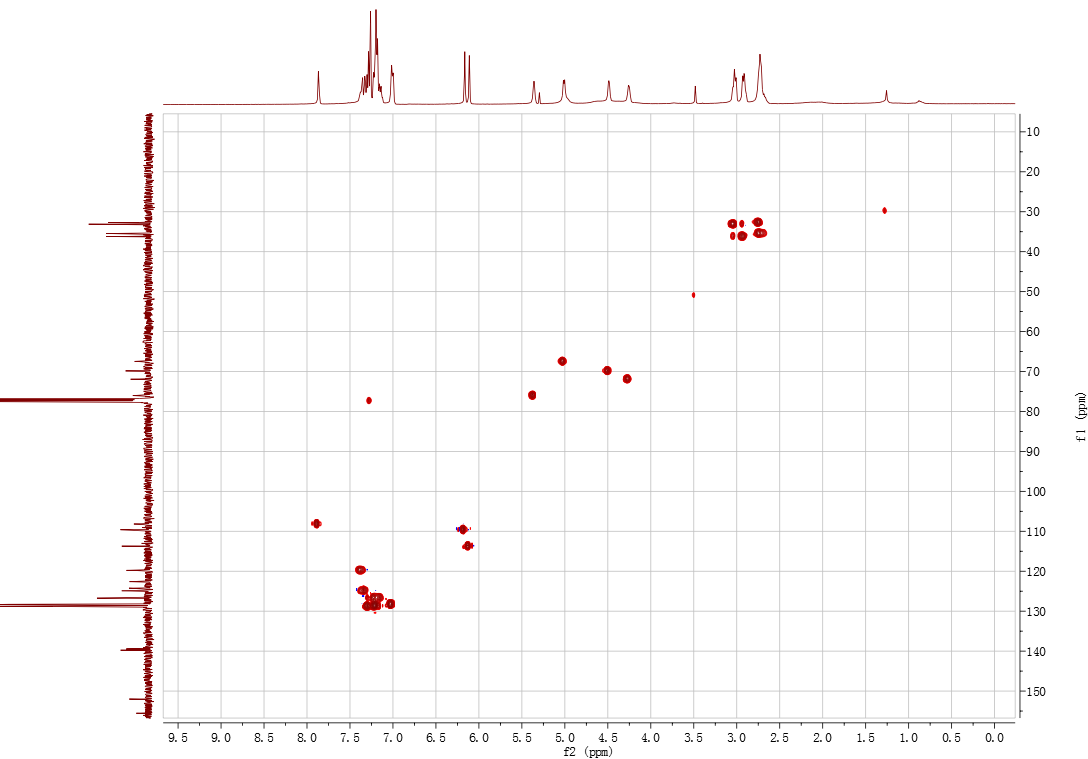


gHSQC spectrum of compound **I** in CDCl_3_


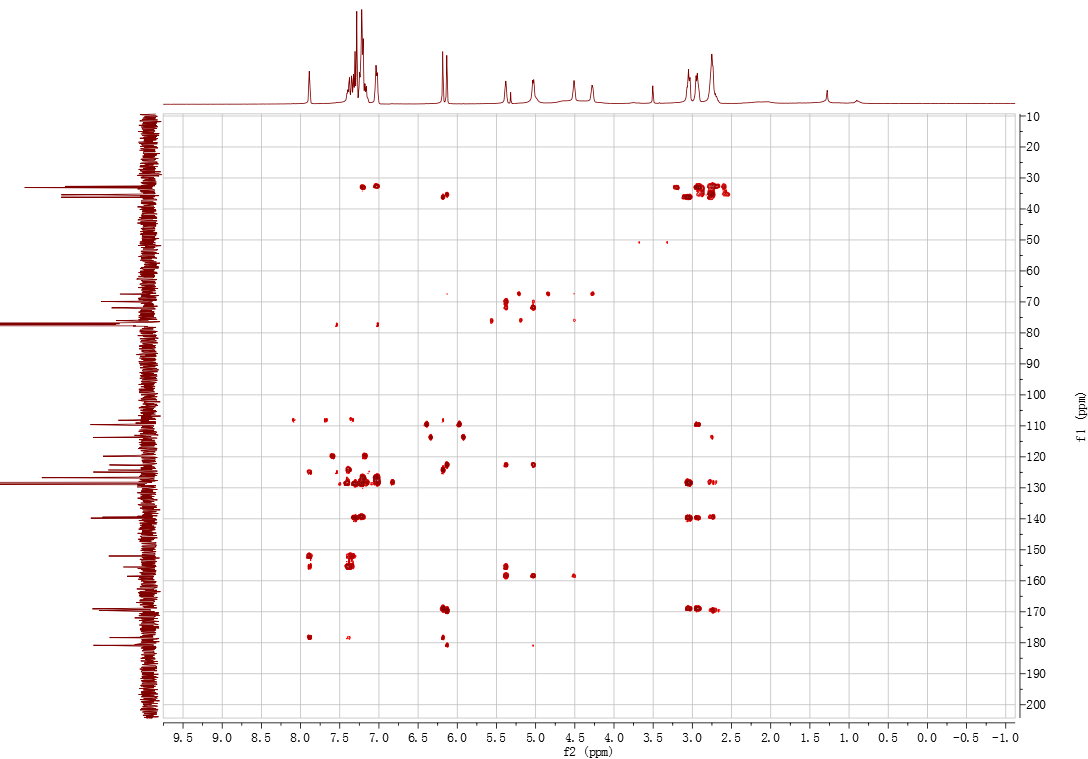


gHMBC spectrum of compound **I** in CDCl_3_


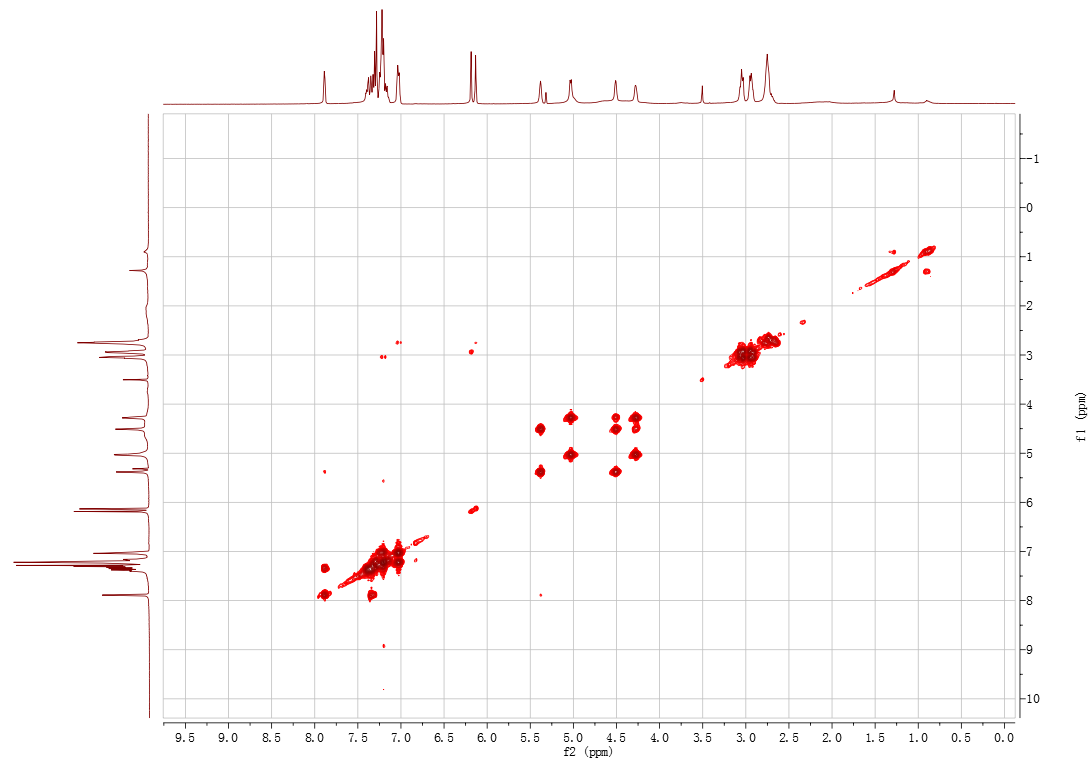


^1^H-^1^H COSY spectrum of compound **I** in CDCl_3_


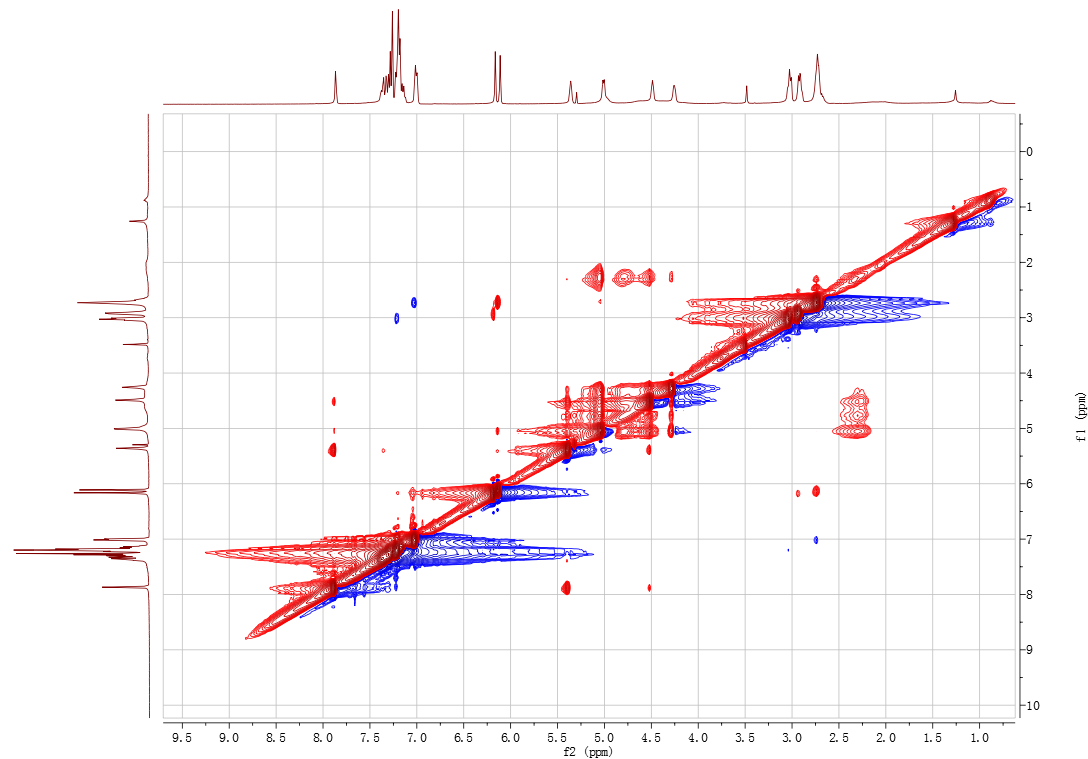


NOESY spectrum of compound **I** in CDCl_3_


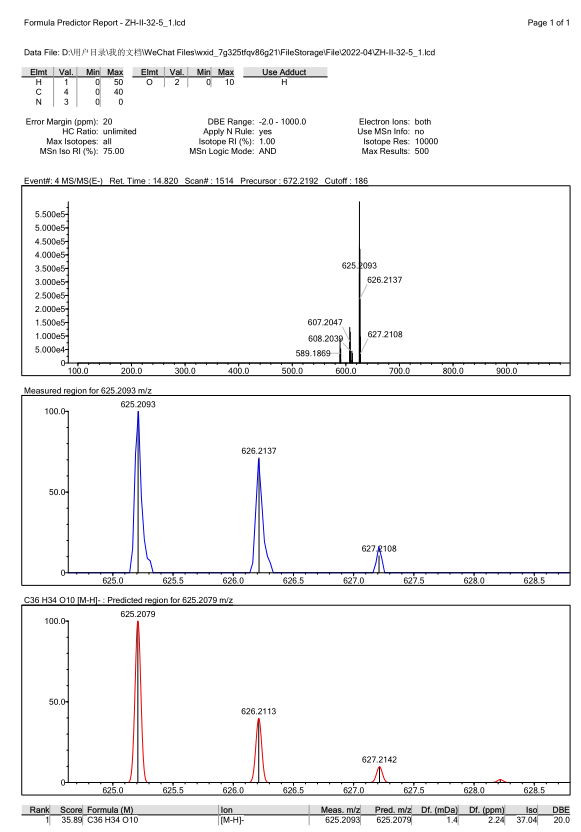


HRESIMS spectrum of compound **II**


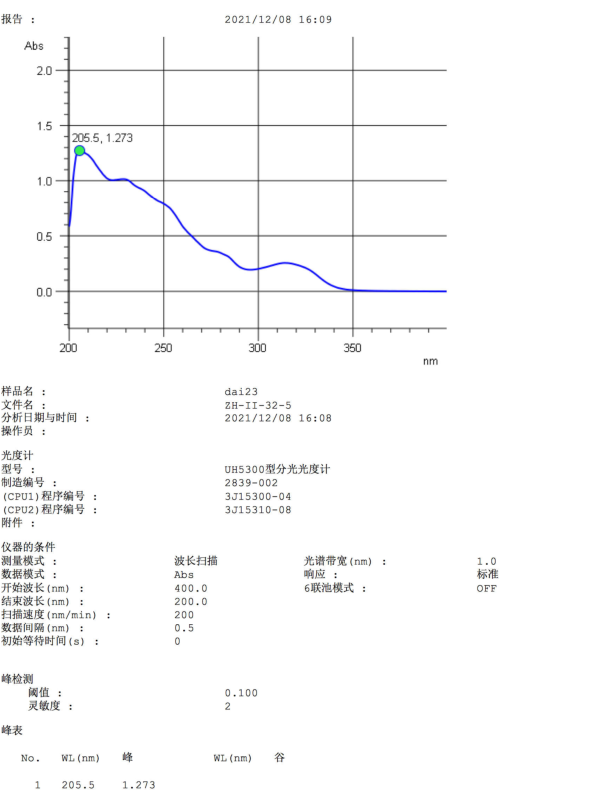


UV spectrum of compound **II**


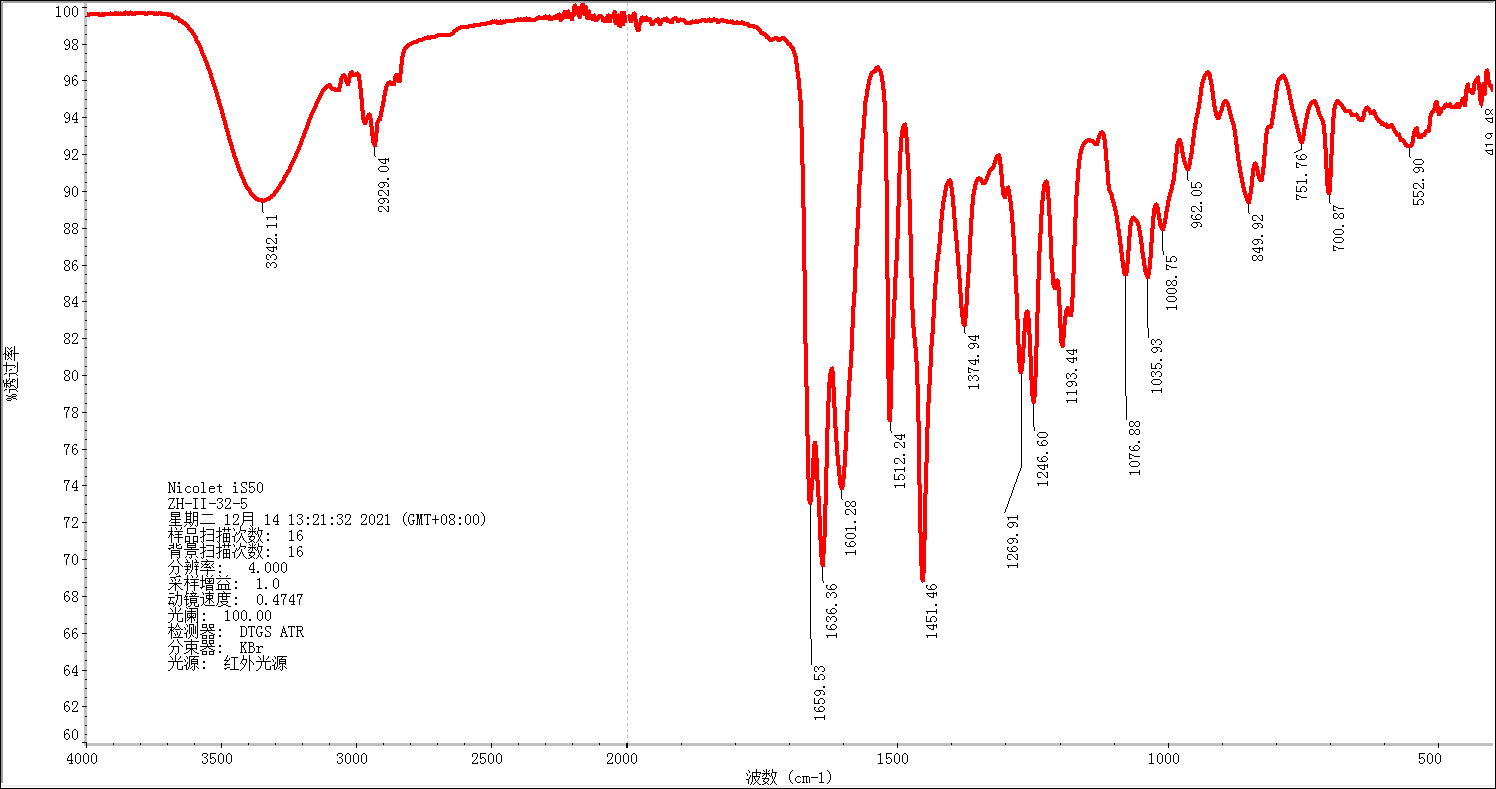


IR spectrum of compound **II**


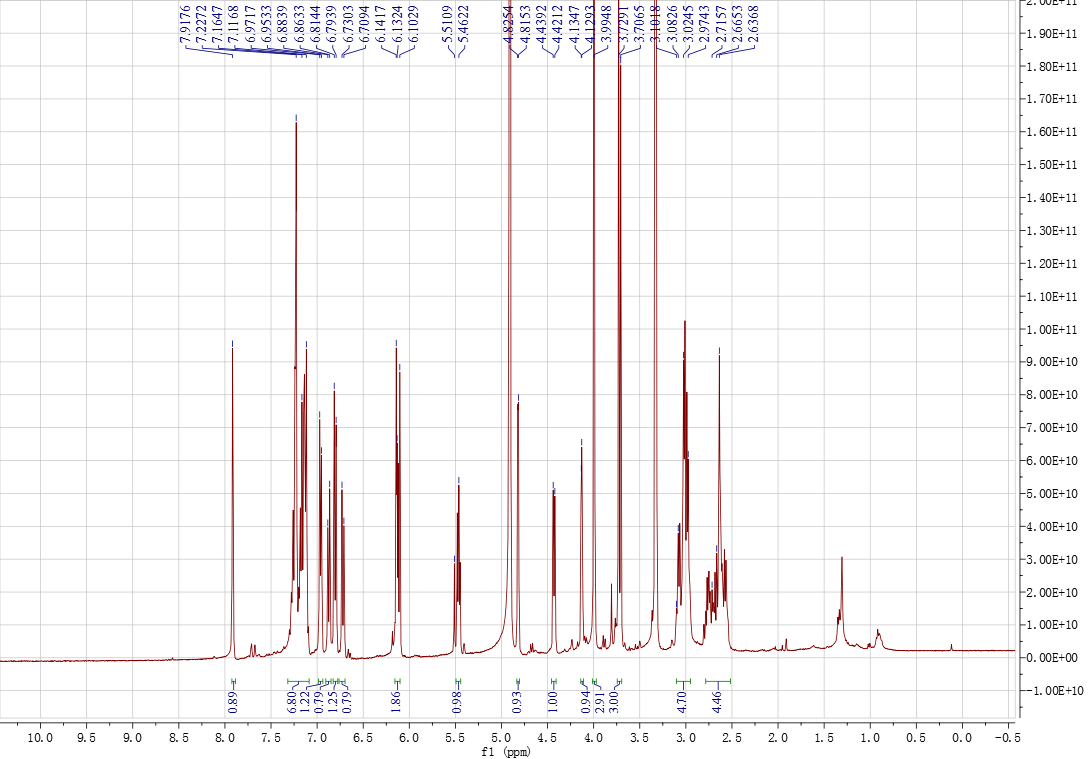


^1^H NMR spectrum of compound **II** in methanol-*d*_4_


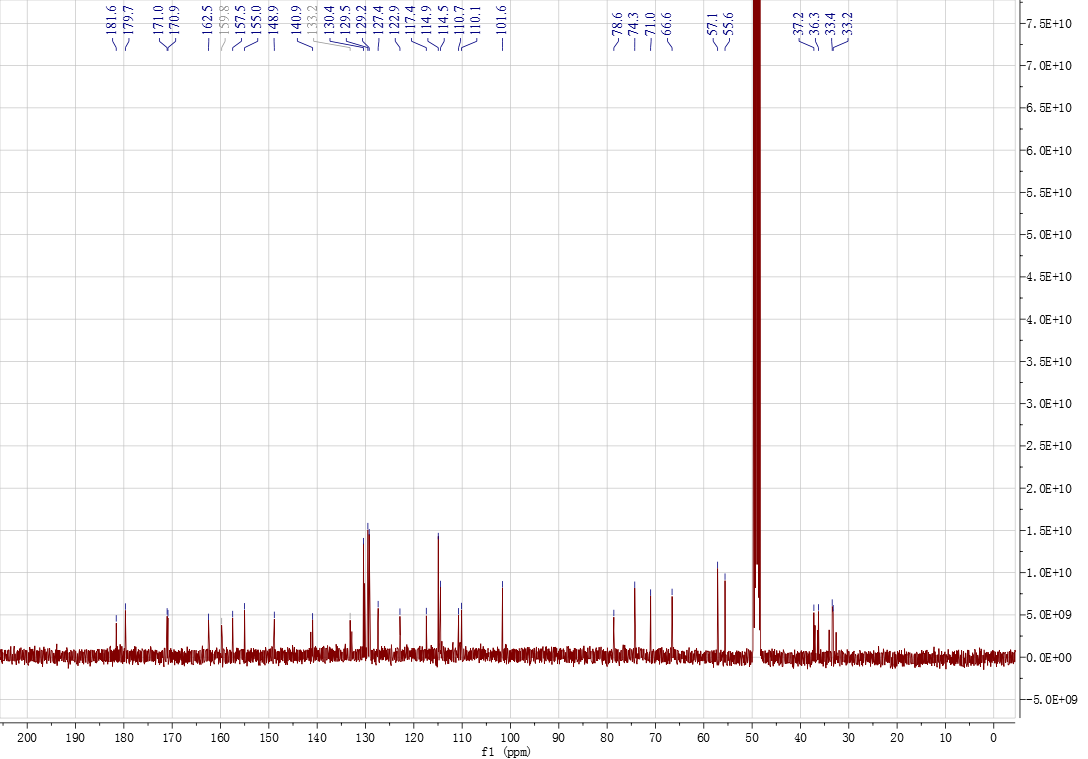


^13^C NMR spectrum of compound **II** in methanol-*d*_4_


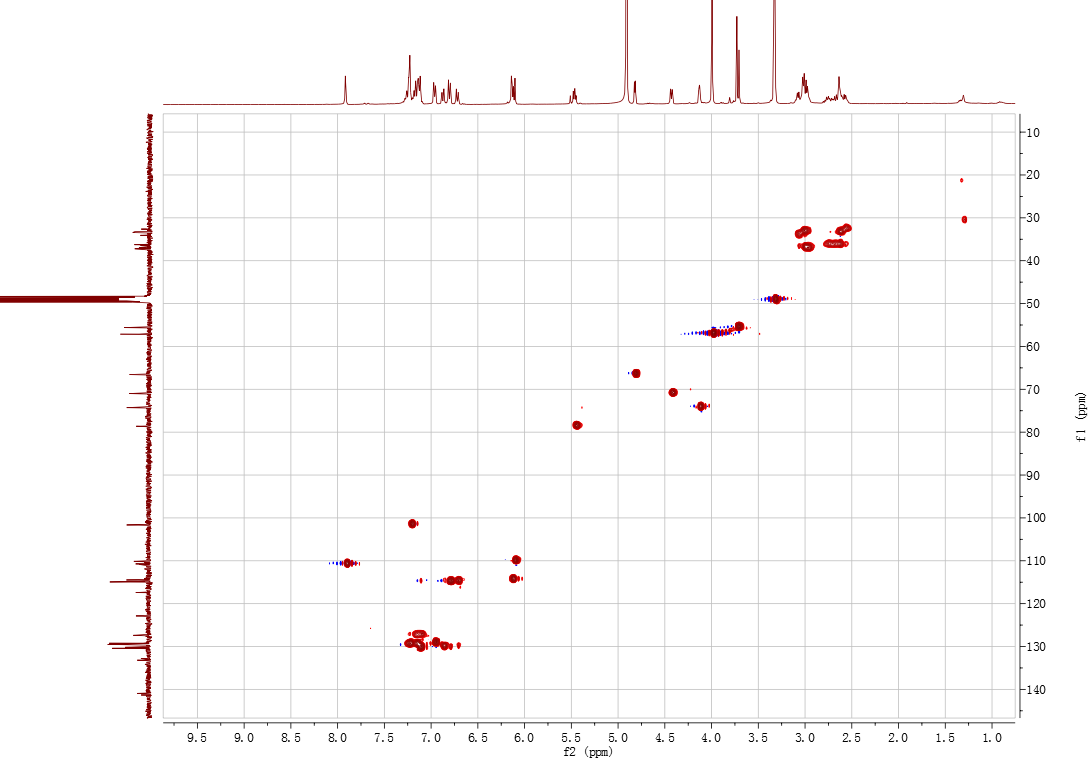


gHSQC spectrum of compound **II** in methanol-*d*_4_


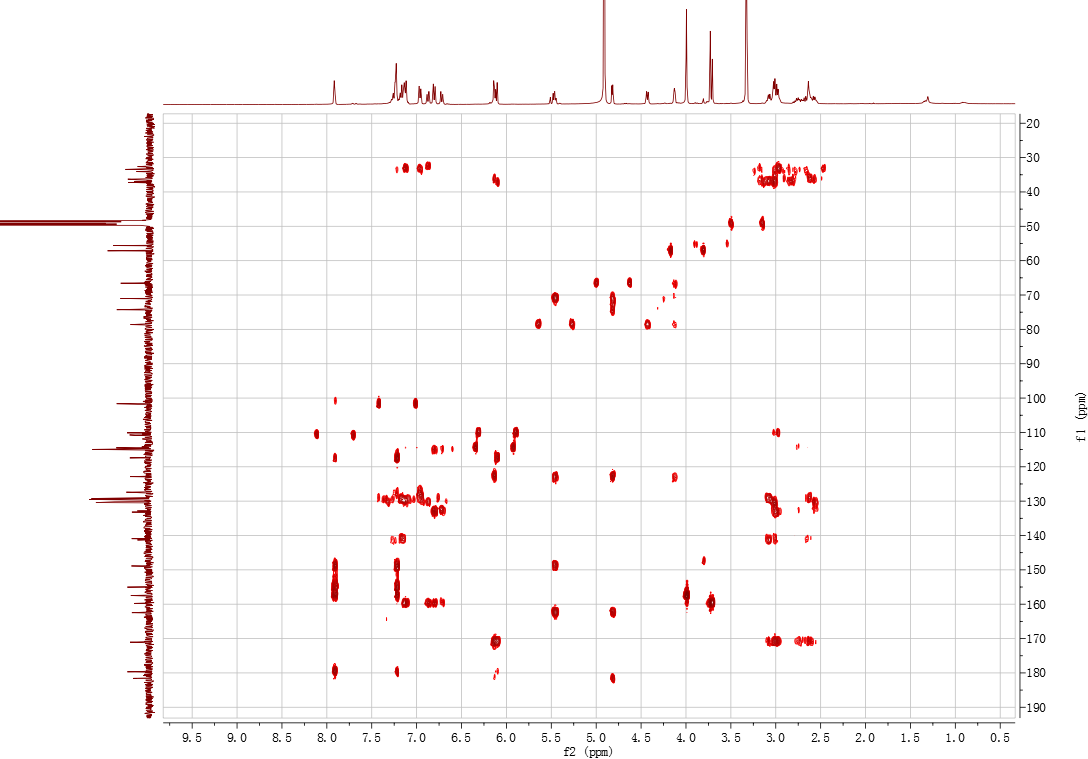


gHMBC spectrum of compound **II** in methanol-*d*_4_


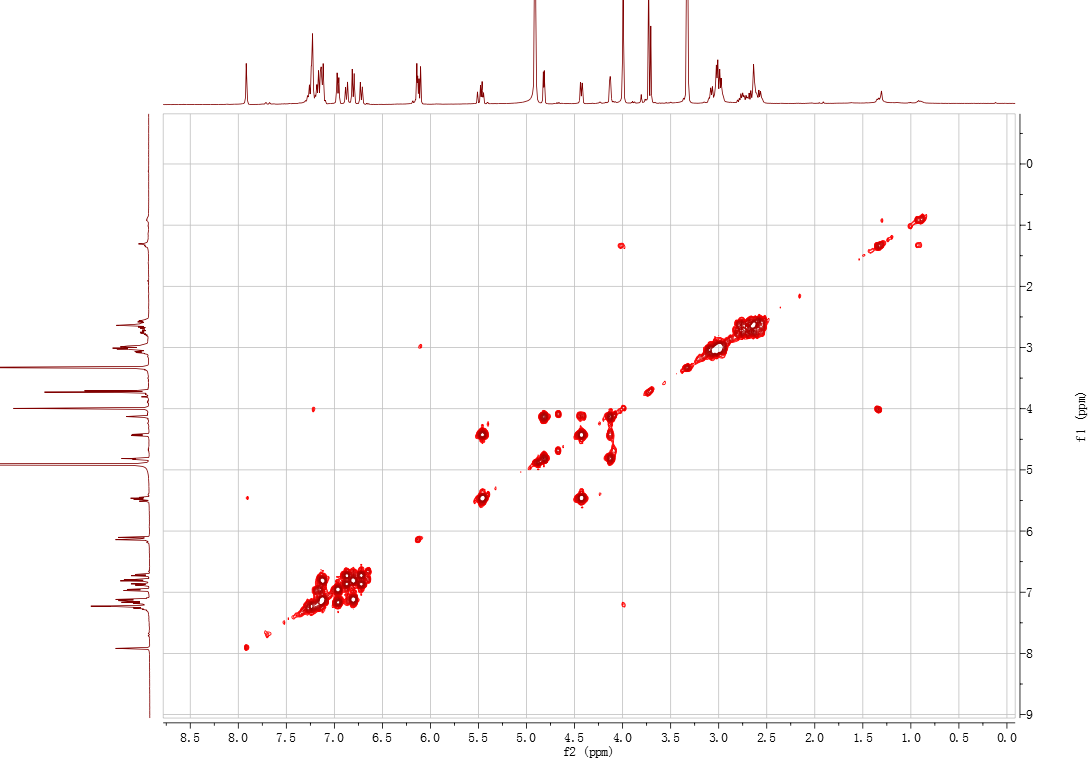


^1^H-^1^H COSY spectrum of compound **II** in methanol-*d*_4_


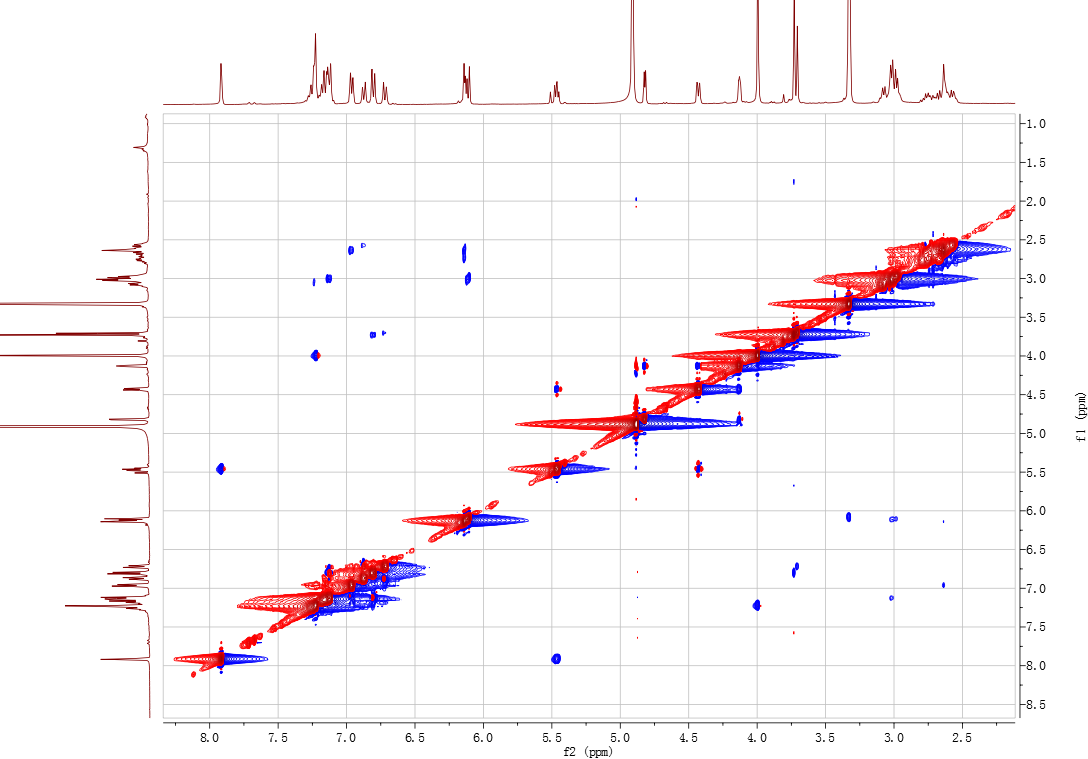


NOESY spectrum of compound **II** in methanol-*d*_4_


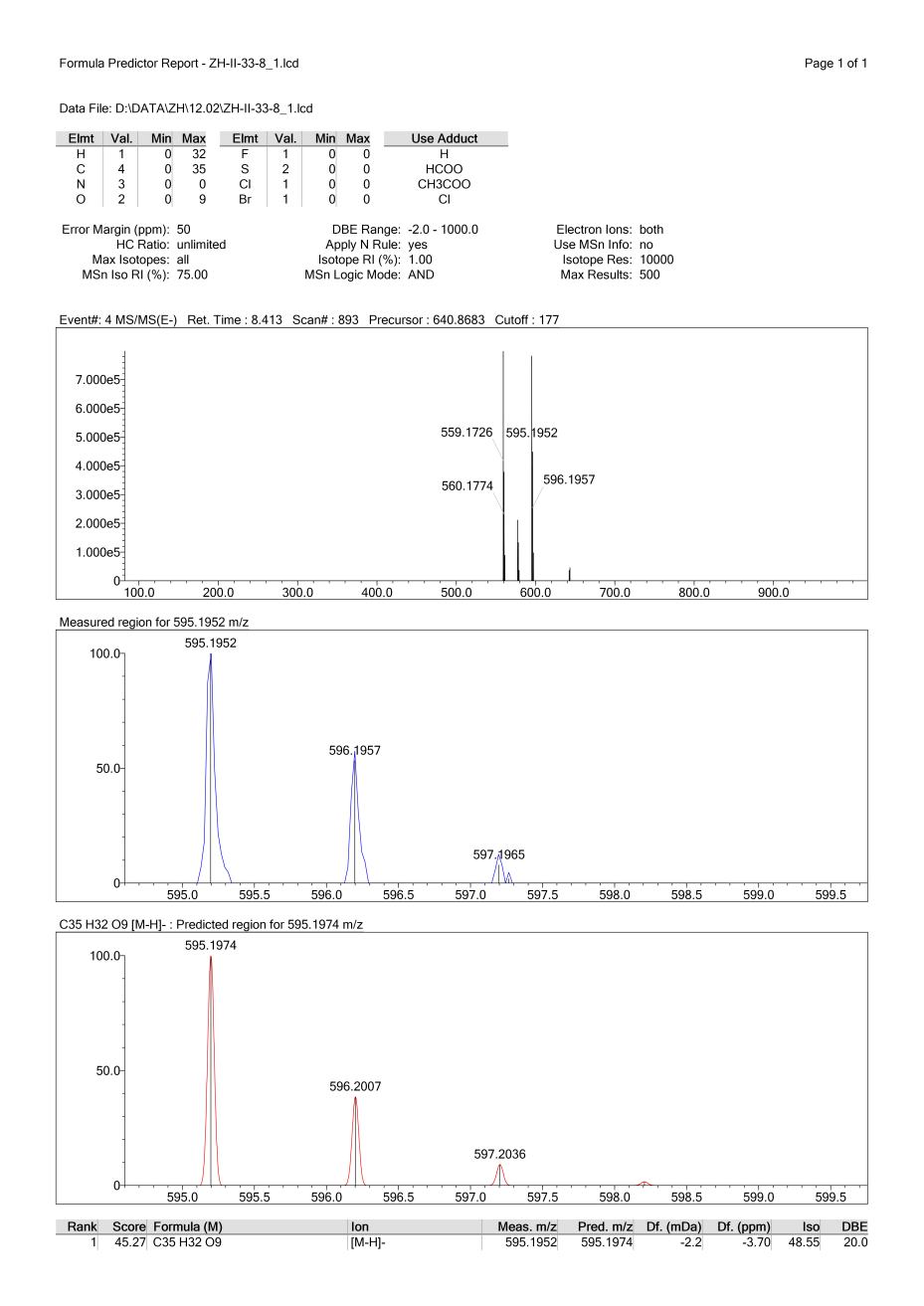


HRESIMS spectrum of compound **III**


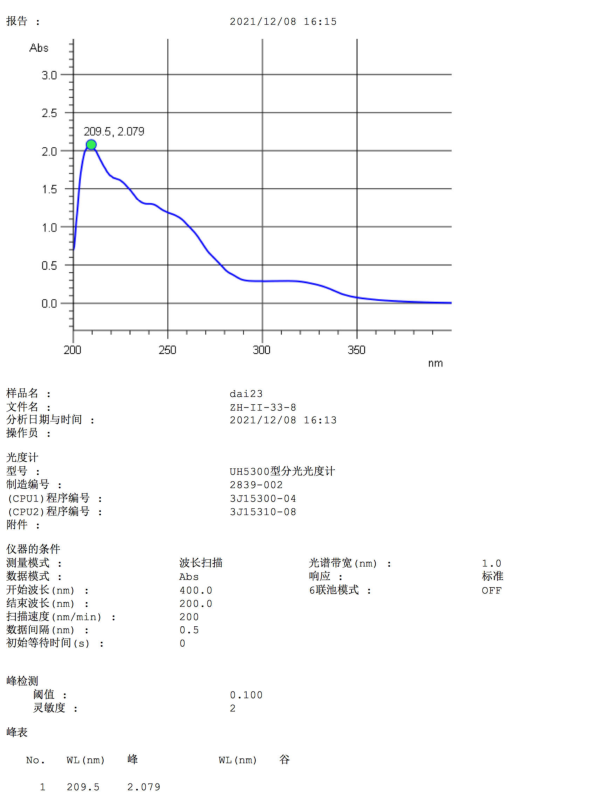


UV spectrum of compound **III**


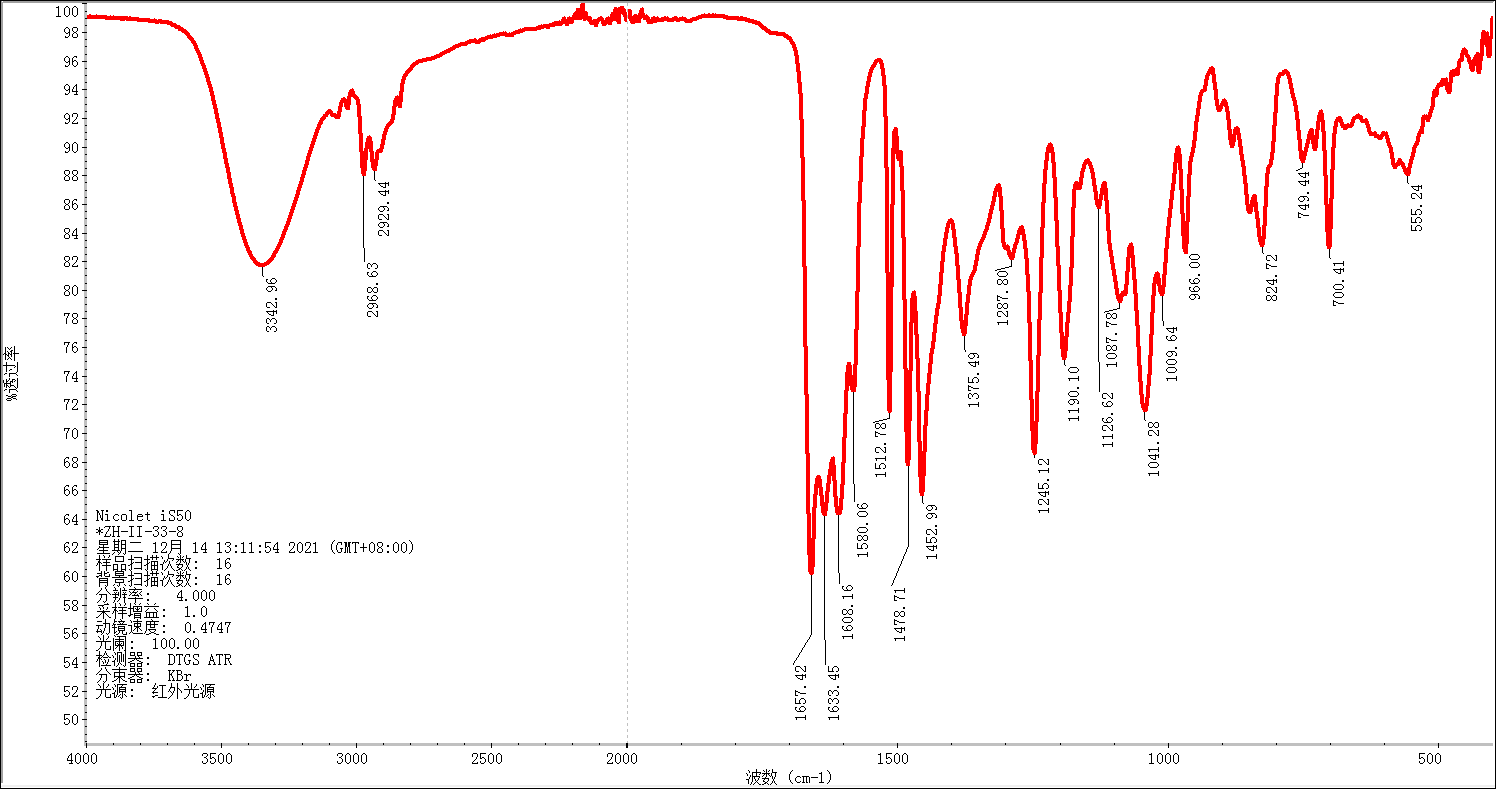


IR spectrum of compound **III**


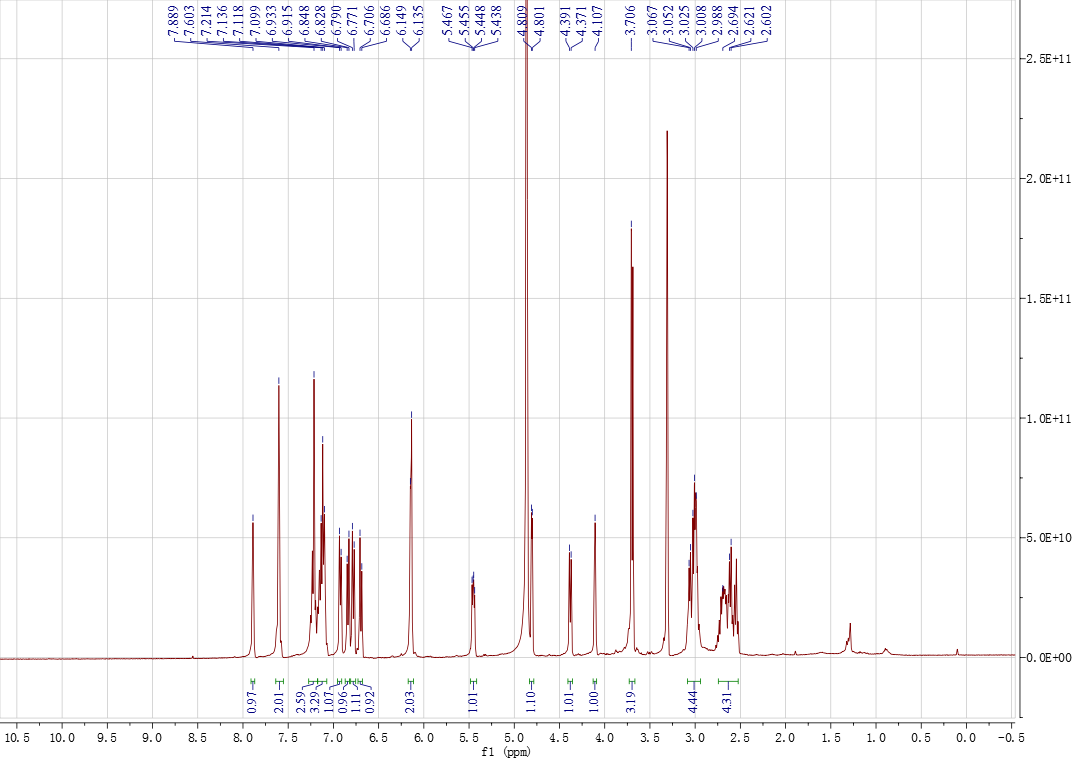


^1^H NMR spectrum of compound **III** in methanol-*d*_4_


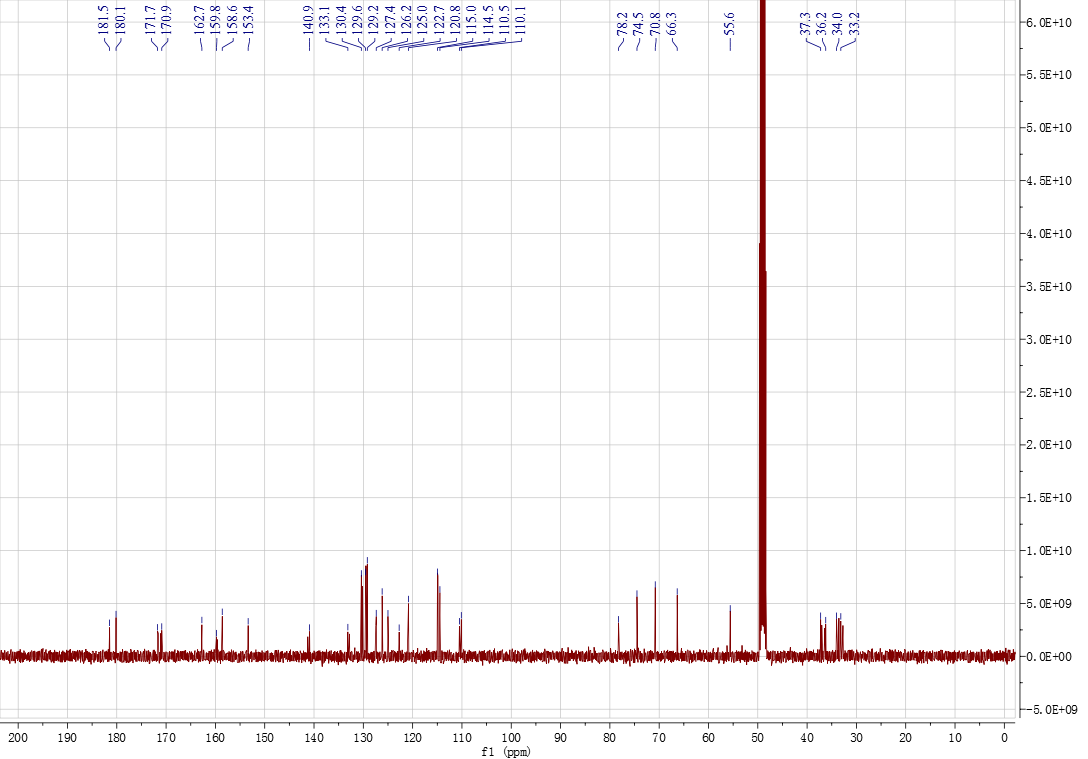


^13^C NMR spectrum of compound **III** in methanol-*d*_4_


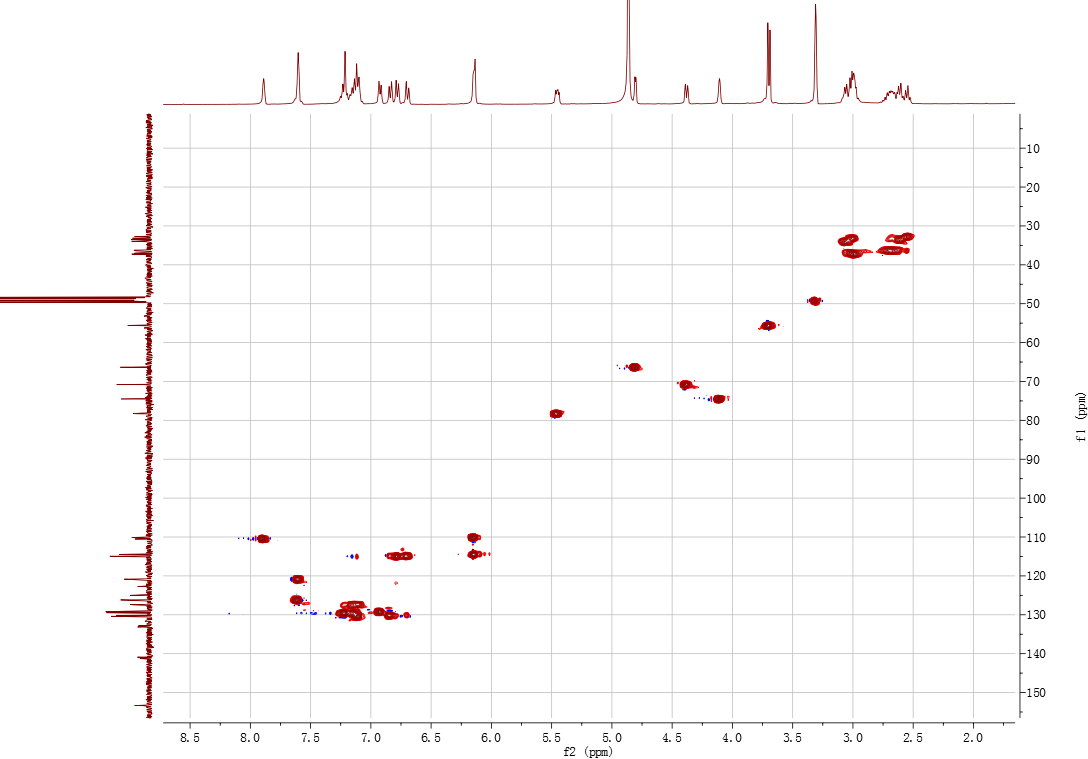


gHSQC spectrum of compound **III** in methanol-*d*_4_


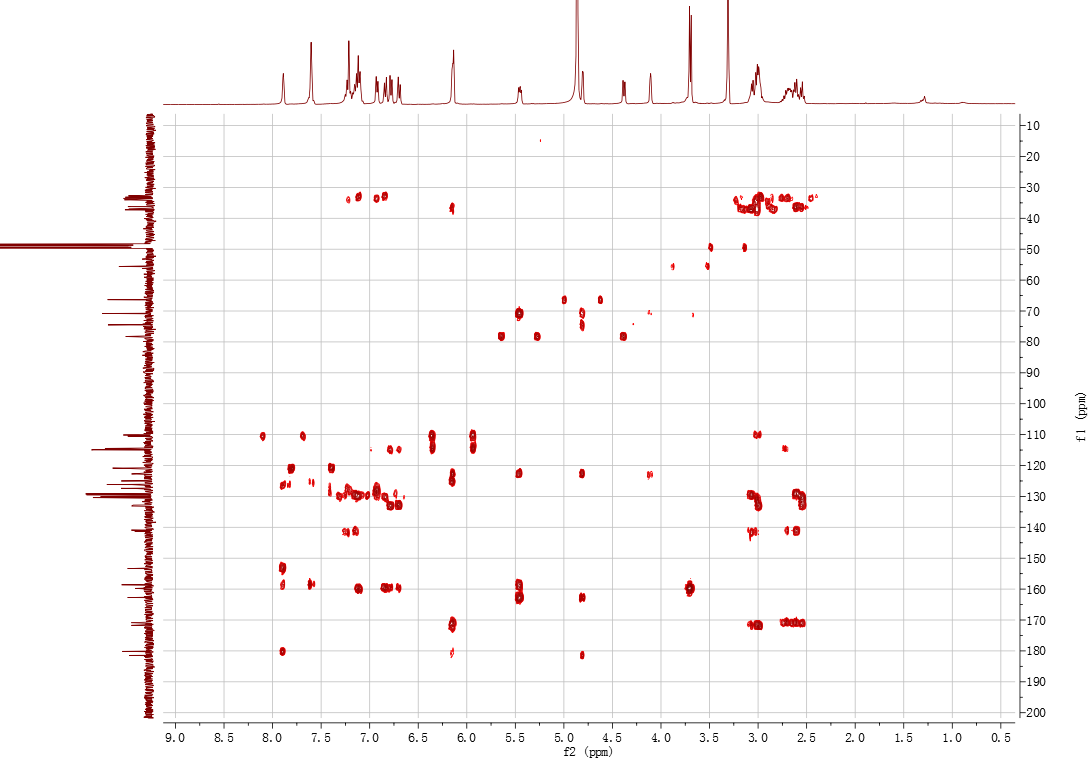


gHMBC spectrum of compound **III** in methanol-*d*_4_


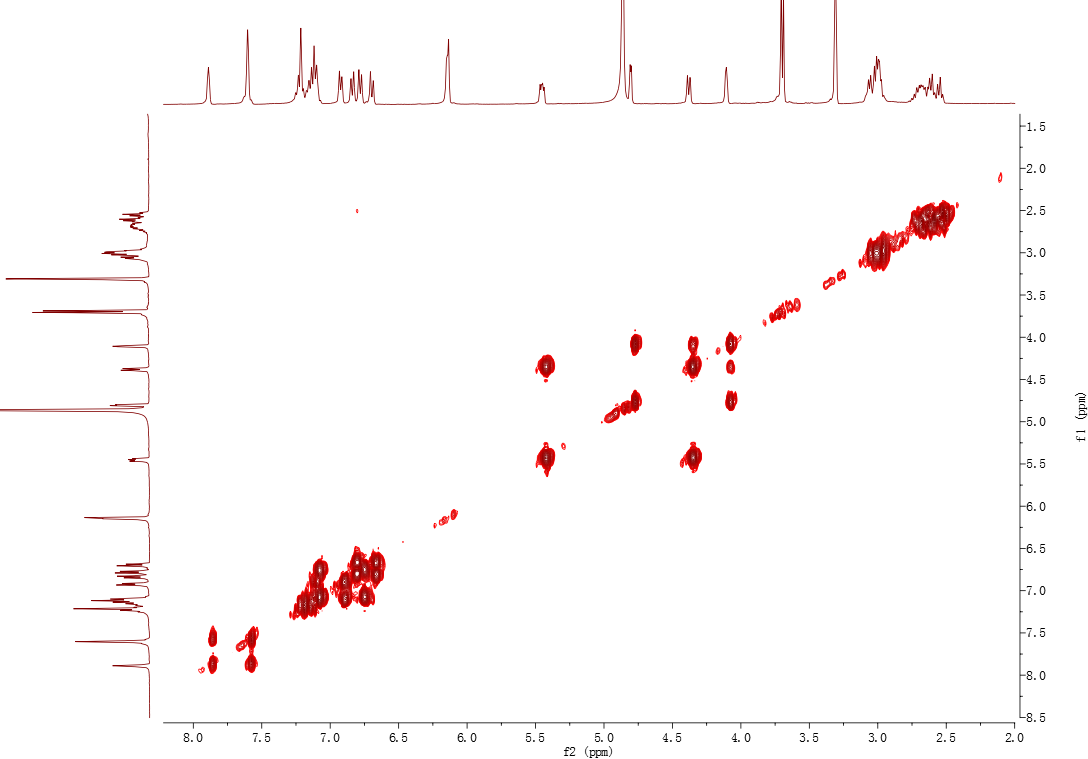


^1^H-^1^H COSY spectrum of compound **III** in methanol-*d*_4_


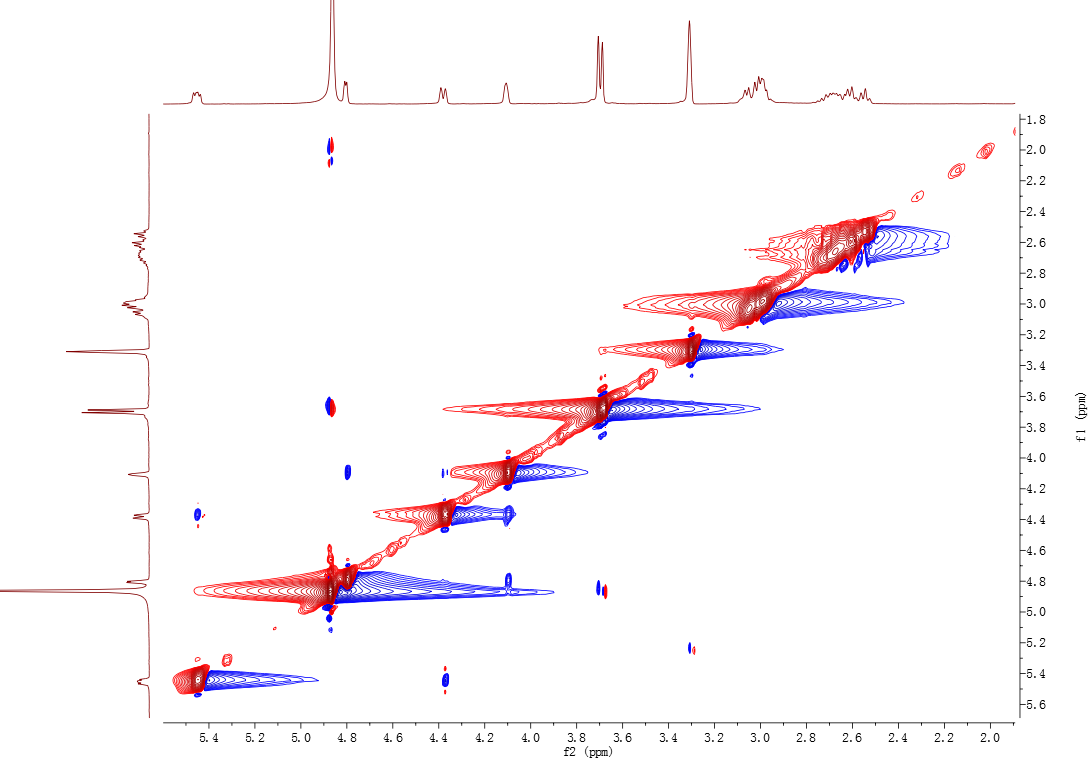


NOESY spectrum of compound **III** in methanol-*d*_4_


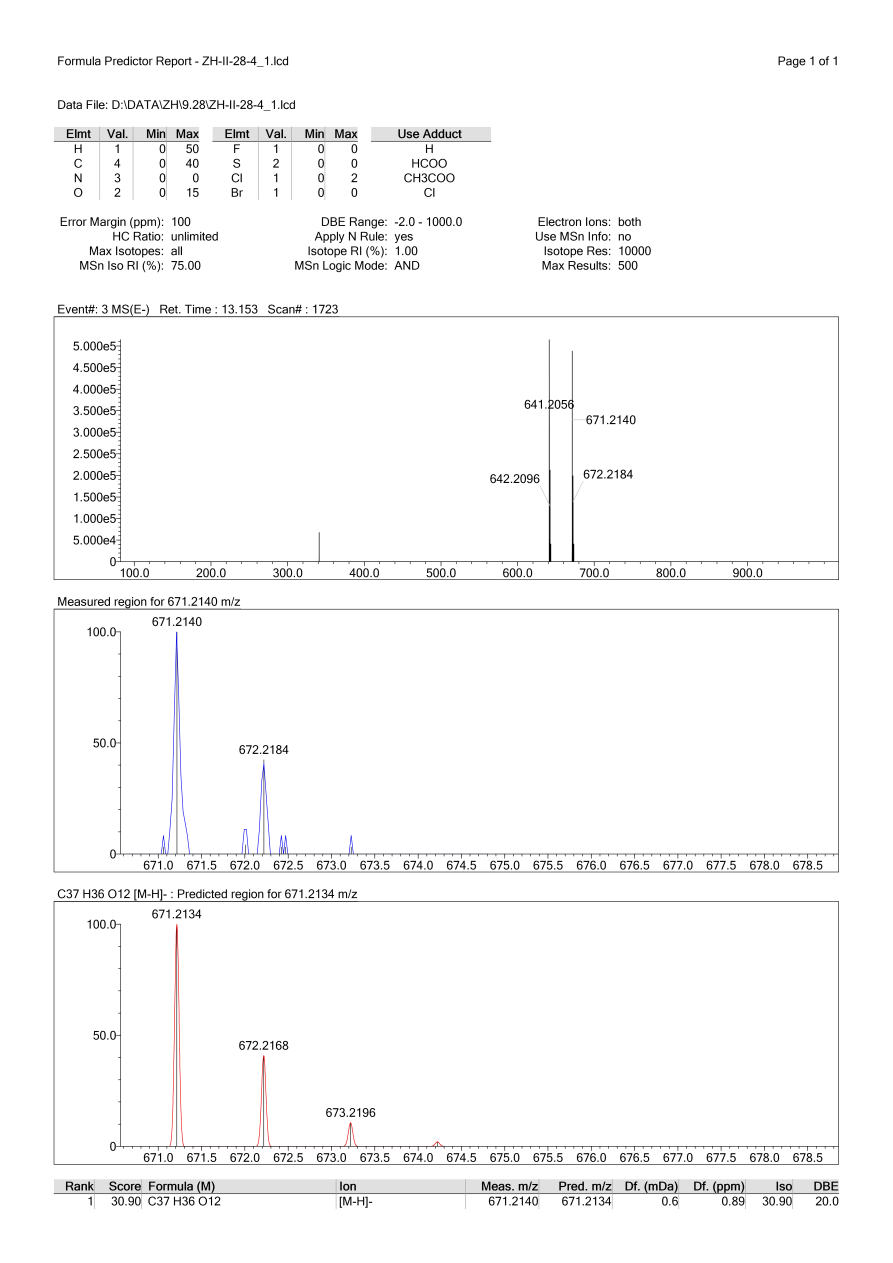


HRESIMS spectrum of compound **IV**


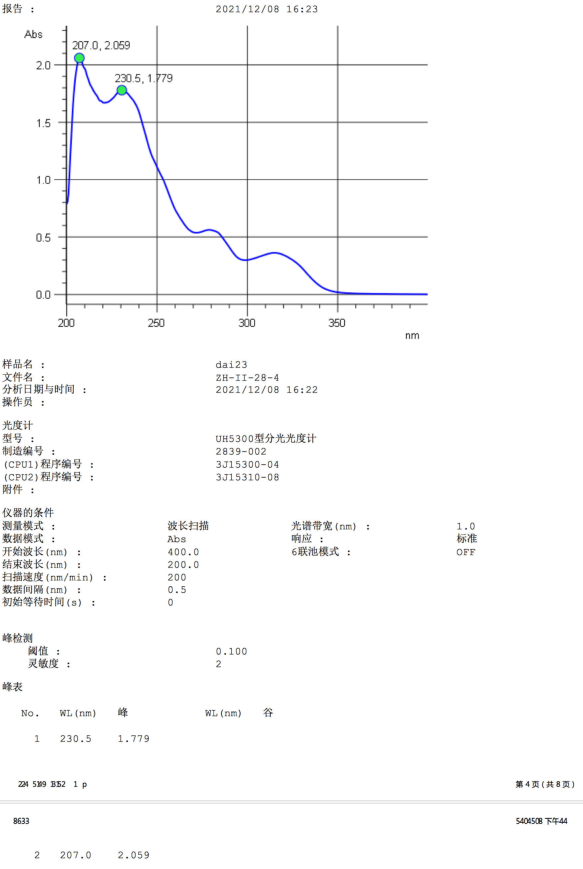


UV spectrum of compound **IV**


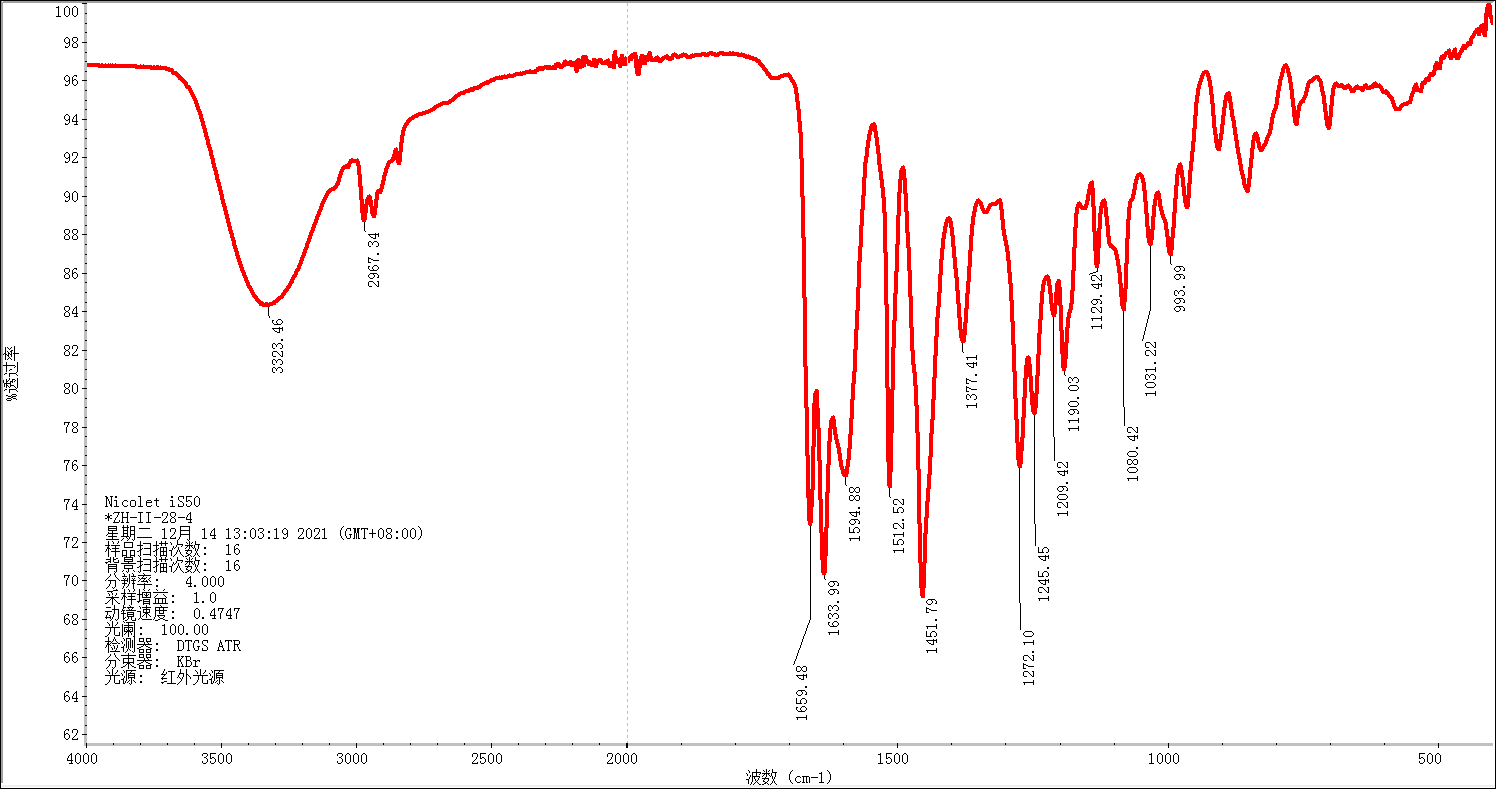


IR spectrum of compound **IV**


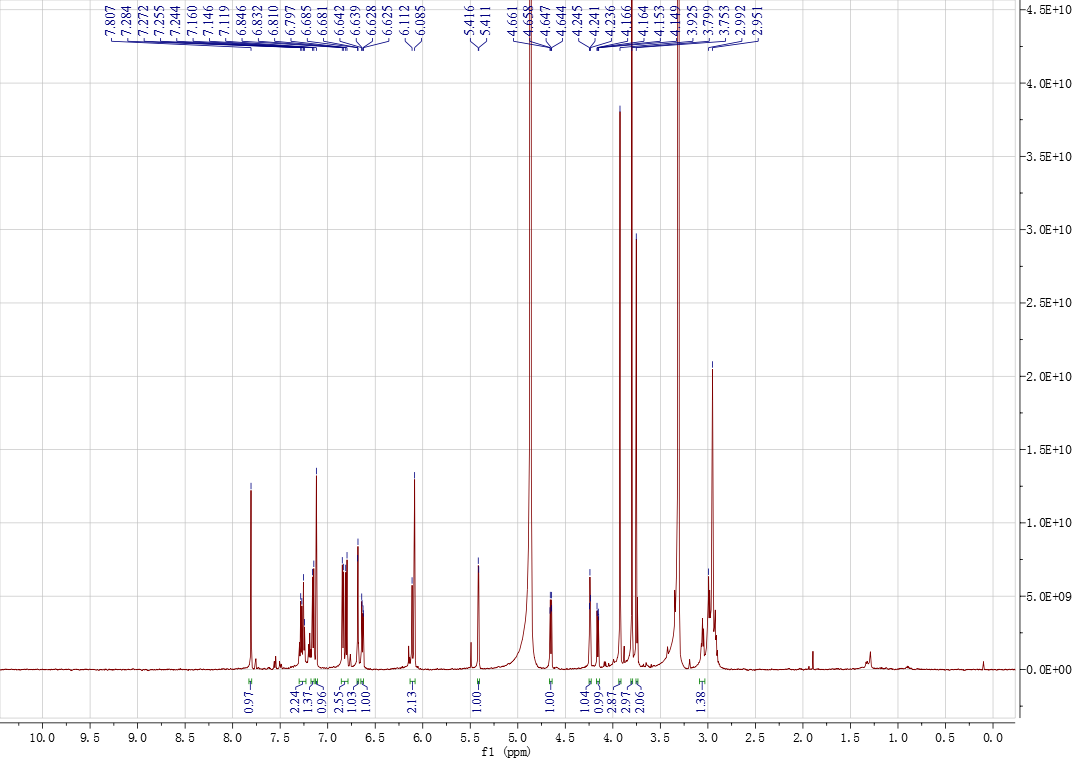


^1^H NMR spectrum of compound **IV** in methanol-*d*_4_


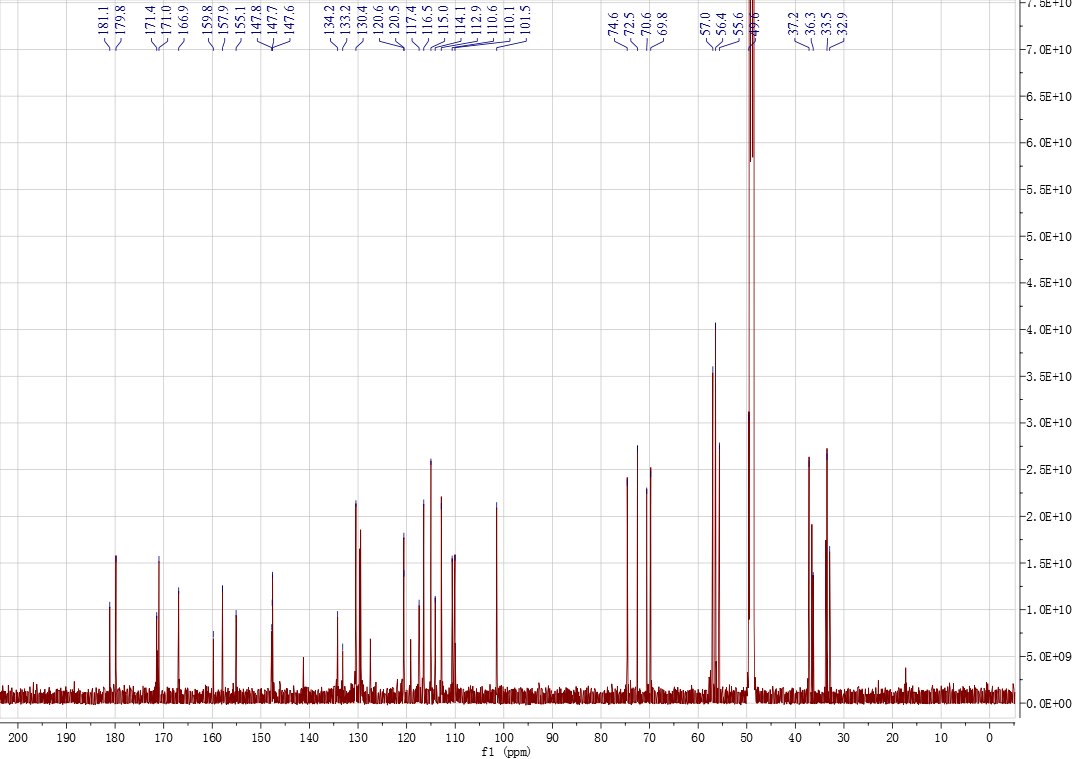


^13^C NMR spectrum of compound **IV** in methanol-*d*_4_


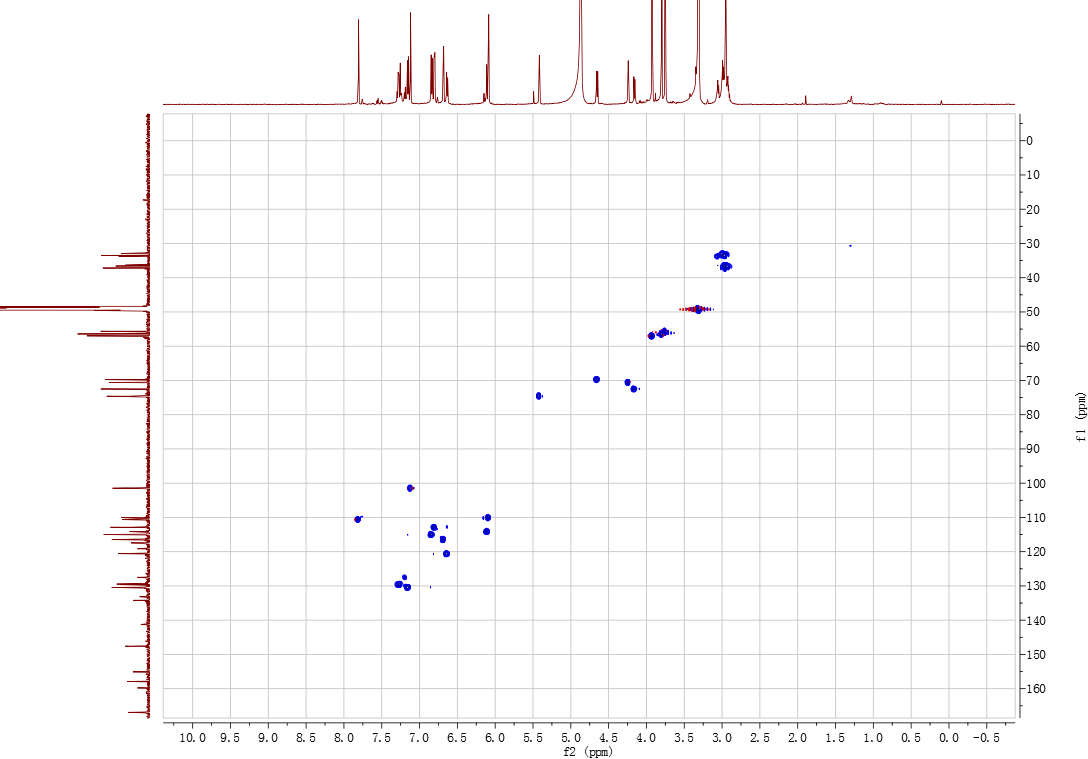


gHSQC spectrum of compound **IV** in methanol-*d*_4_


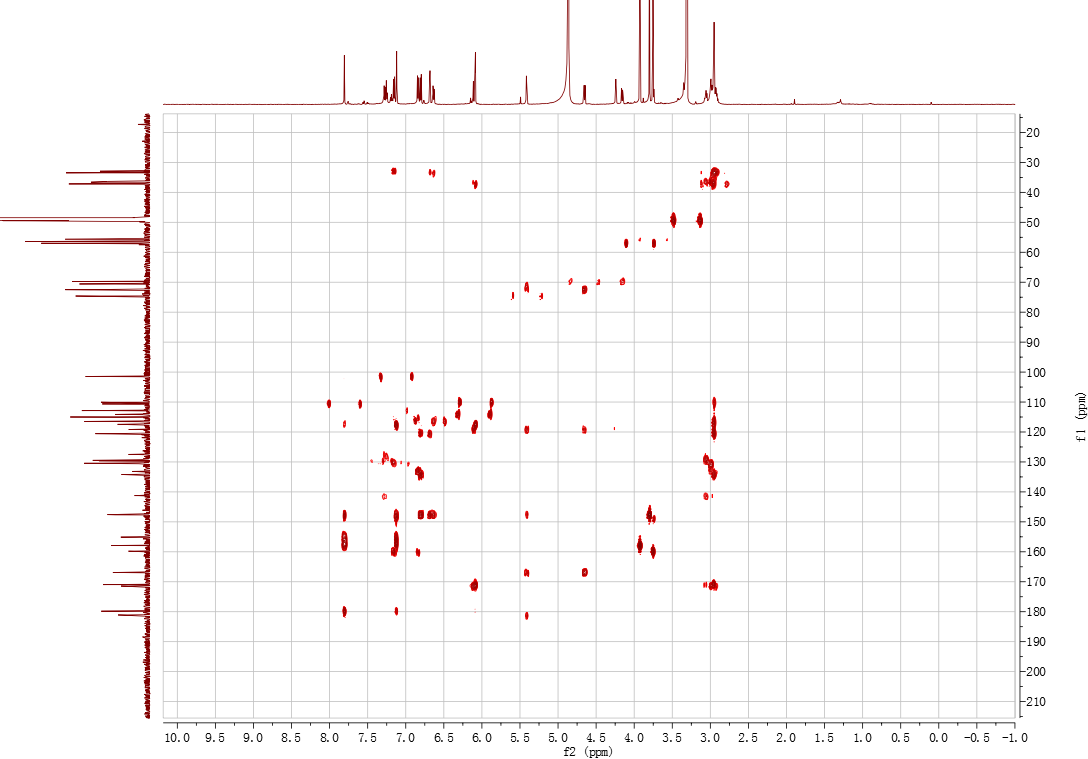


gHMBC spectrum of compound **IV** in methanol-*d*_4_


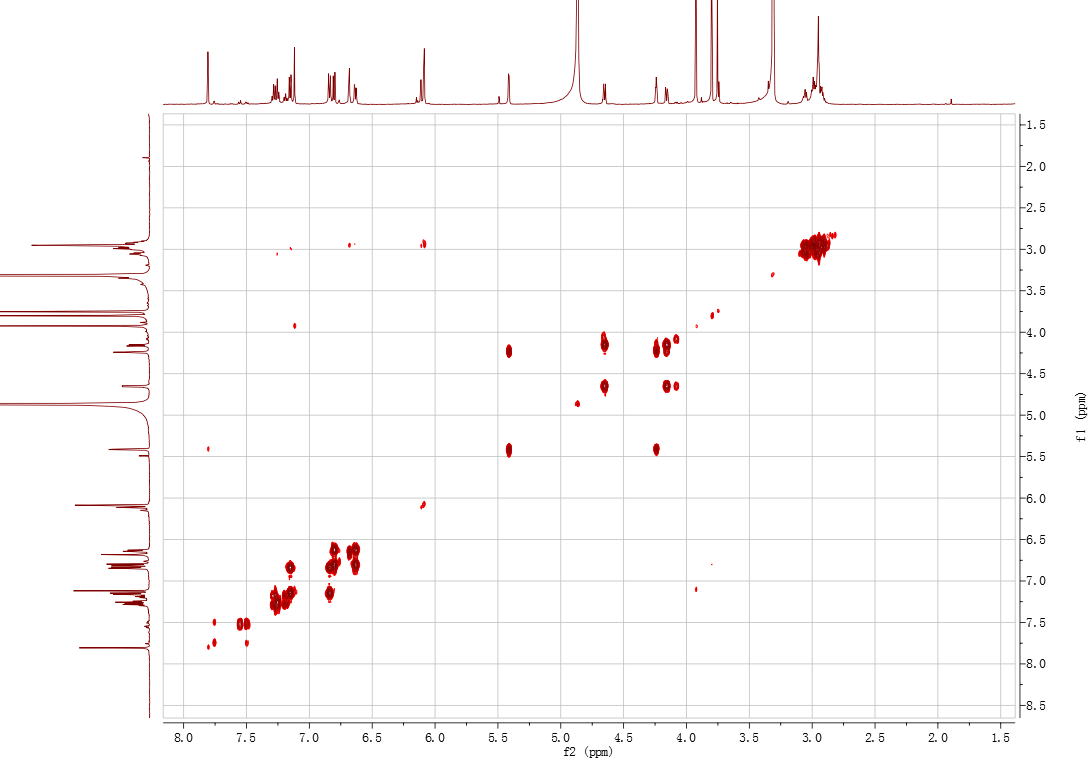


^1^H-^1^H COSY spectrum of compound **IV** in methanol-*d*_4_


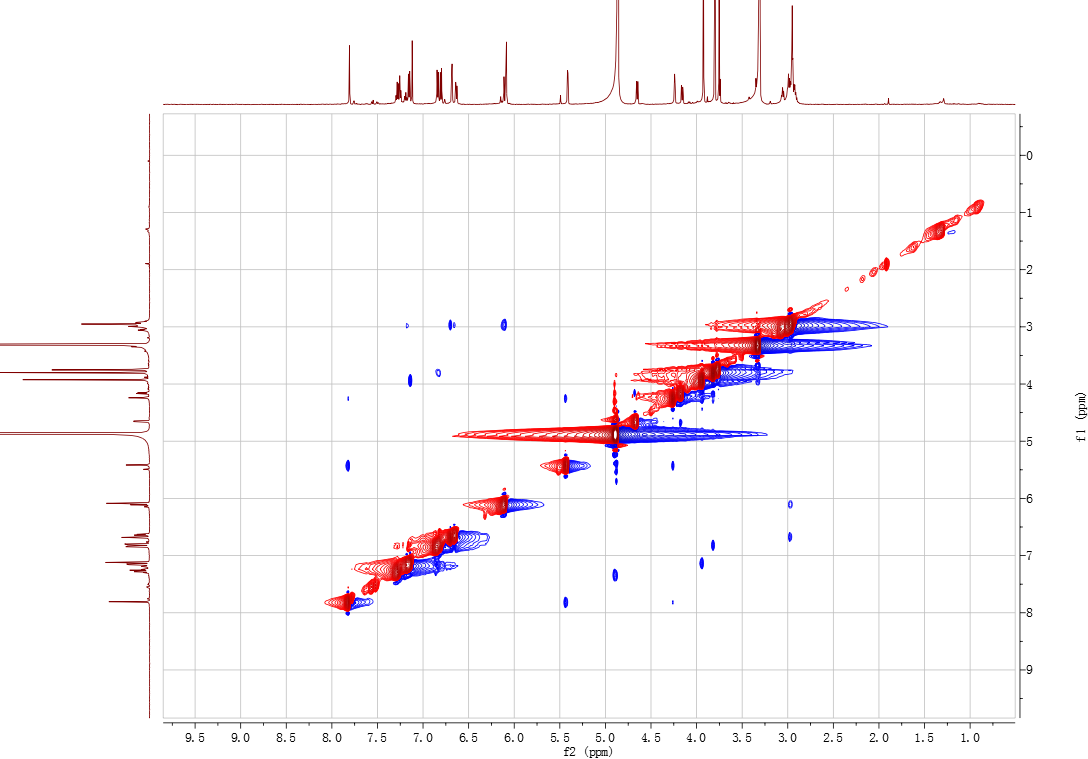


NOESY spectrum of compound **IV** in methanol-*d*_4_


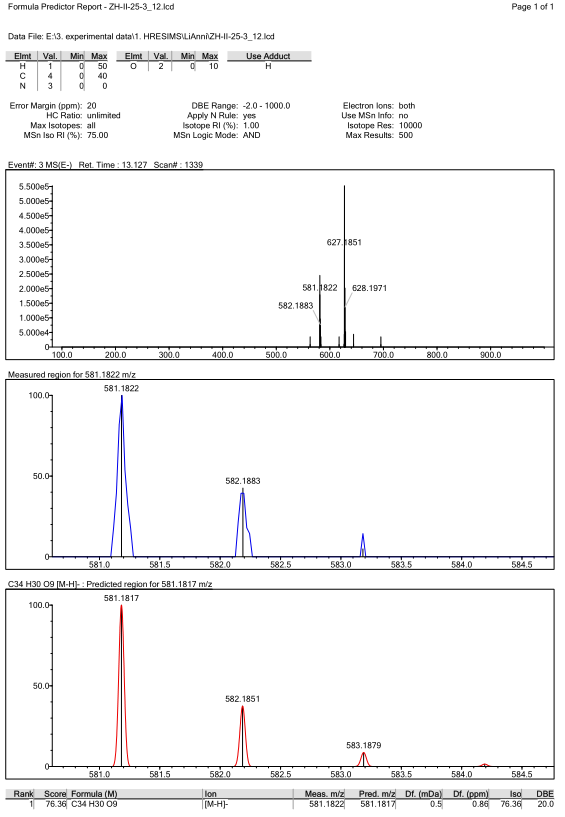


HRESIMS spectrum of compound **V**


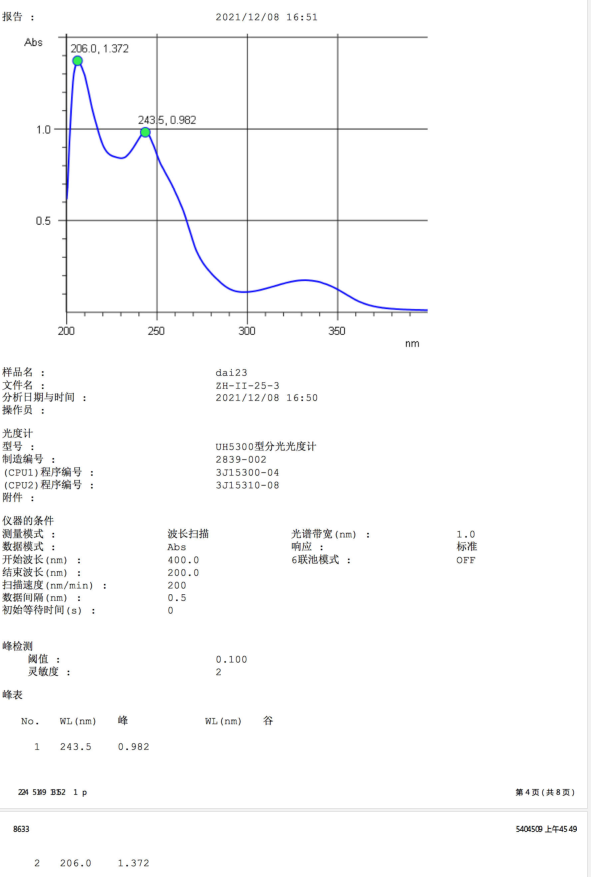


UV spectrum of compound **V**


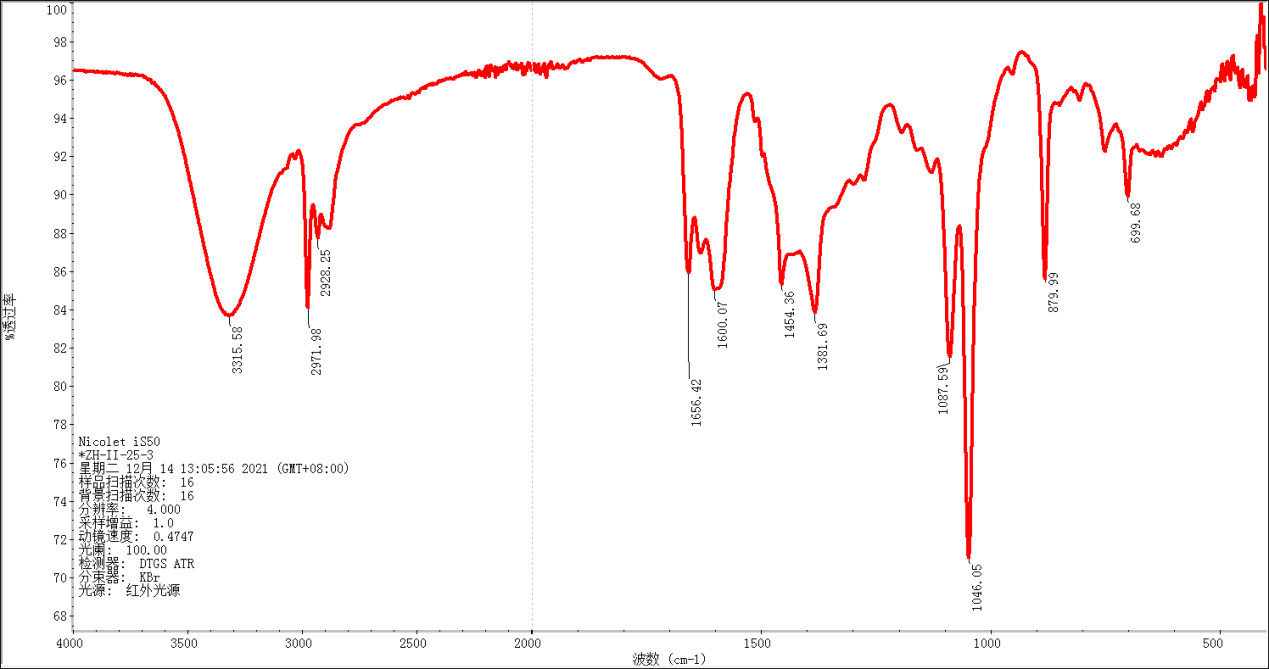


IR spectrum of compound **V**


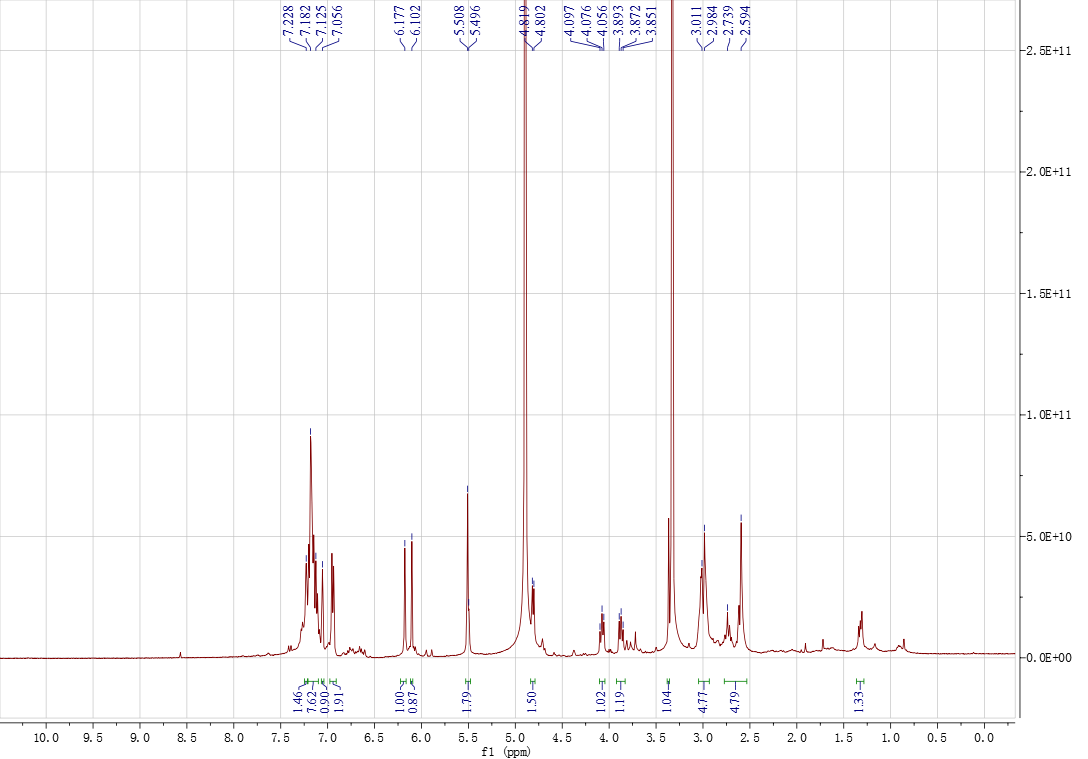


^1^H NMR spectrum of compound **V** in methanol-*d*_4_


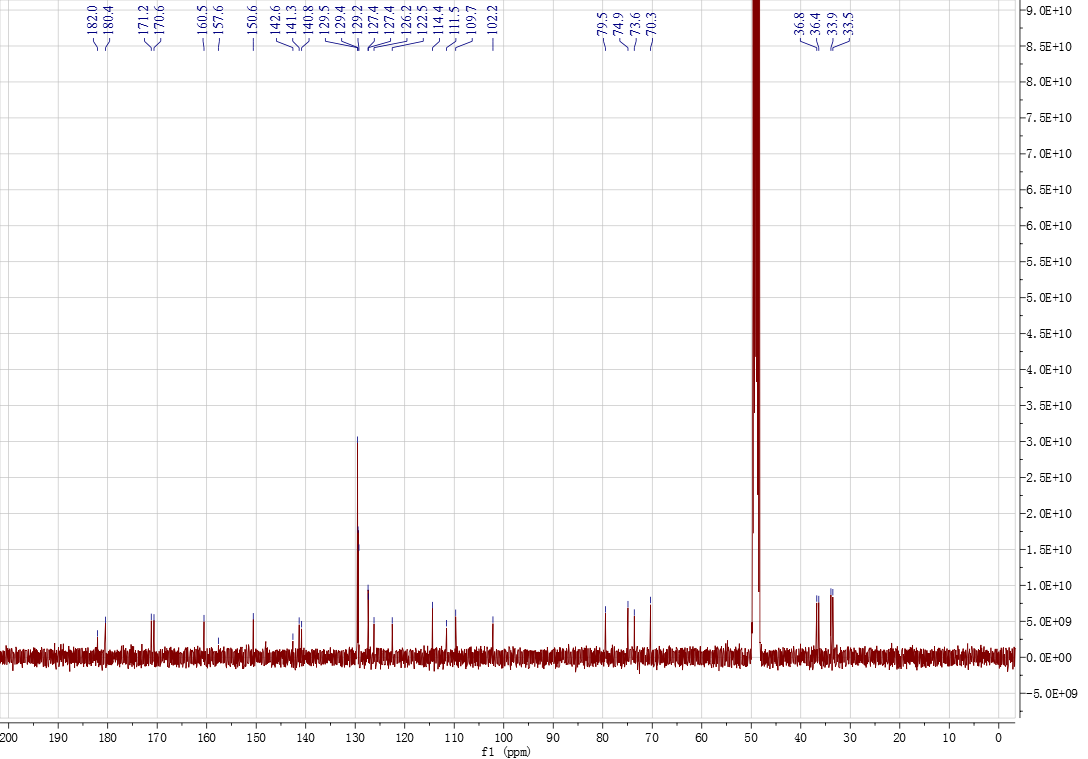


^13^C NMR spectrum of compound **V** in methanol-*d*_4_


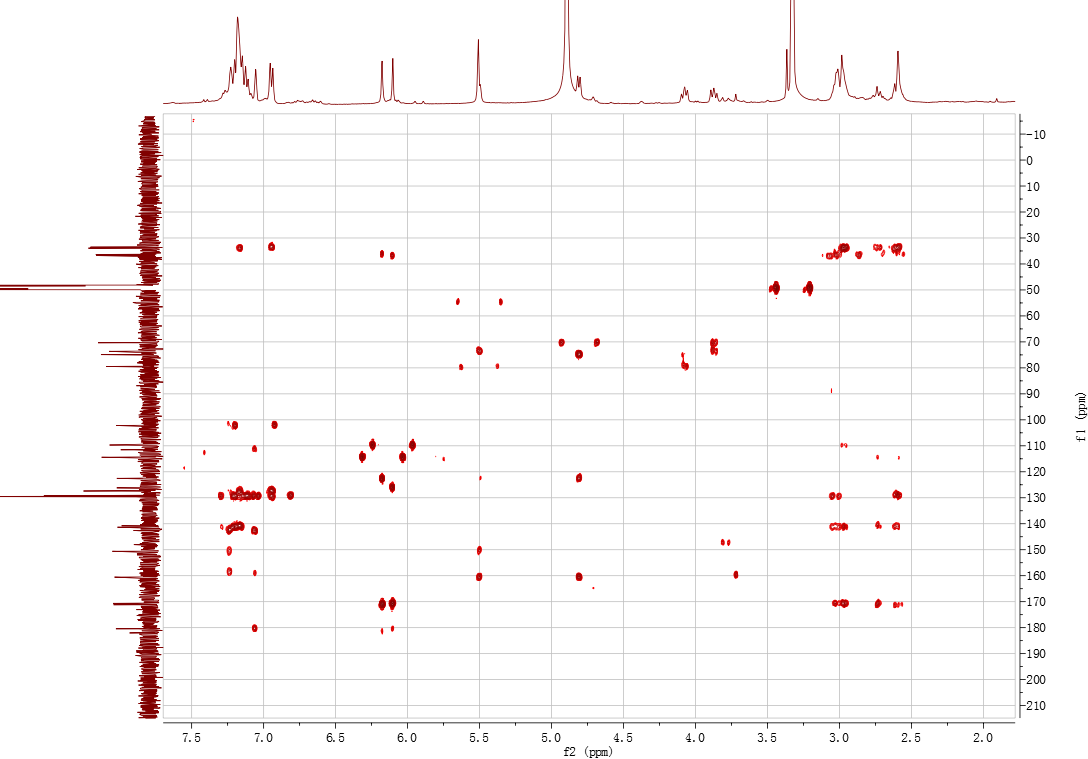


gHSQC spectrum of compound **V** in methanol-*d*_4_


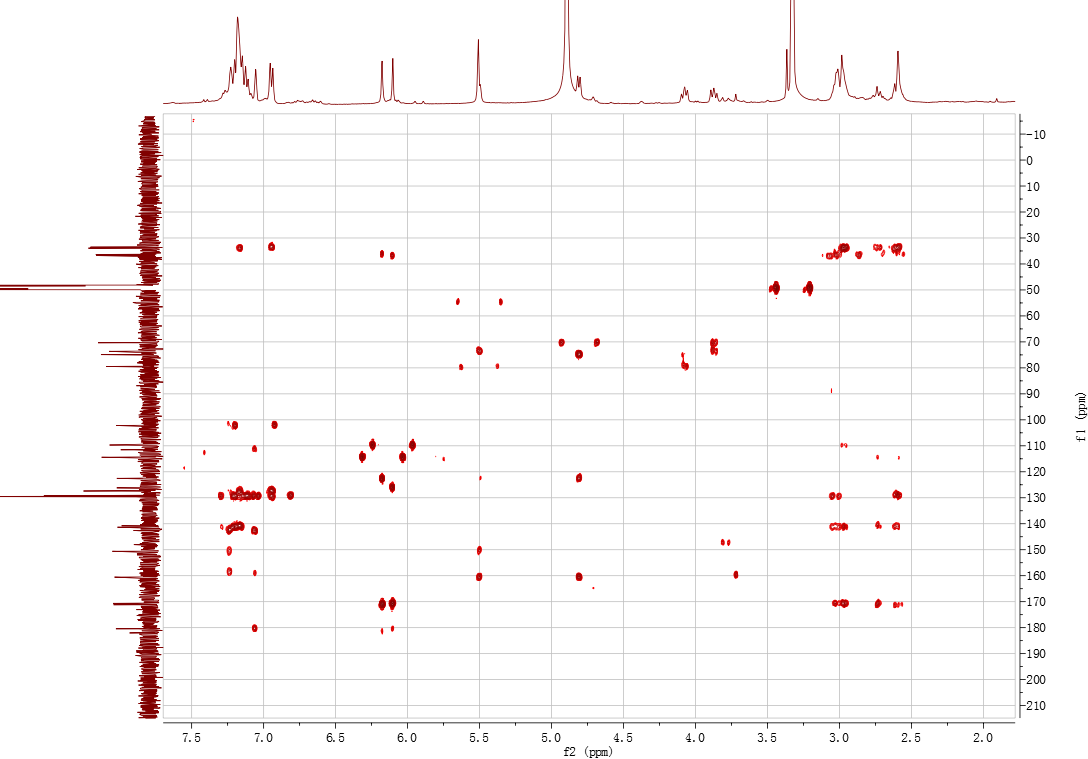


gHMBC spectrum of compound **V** in methanol-*d*_4_


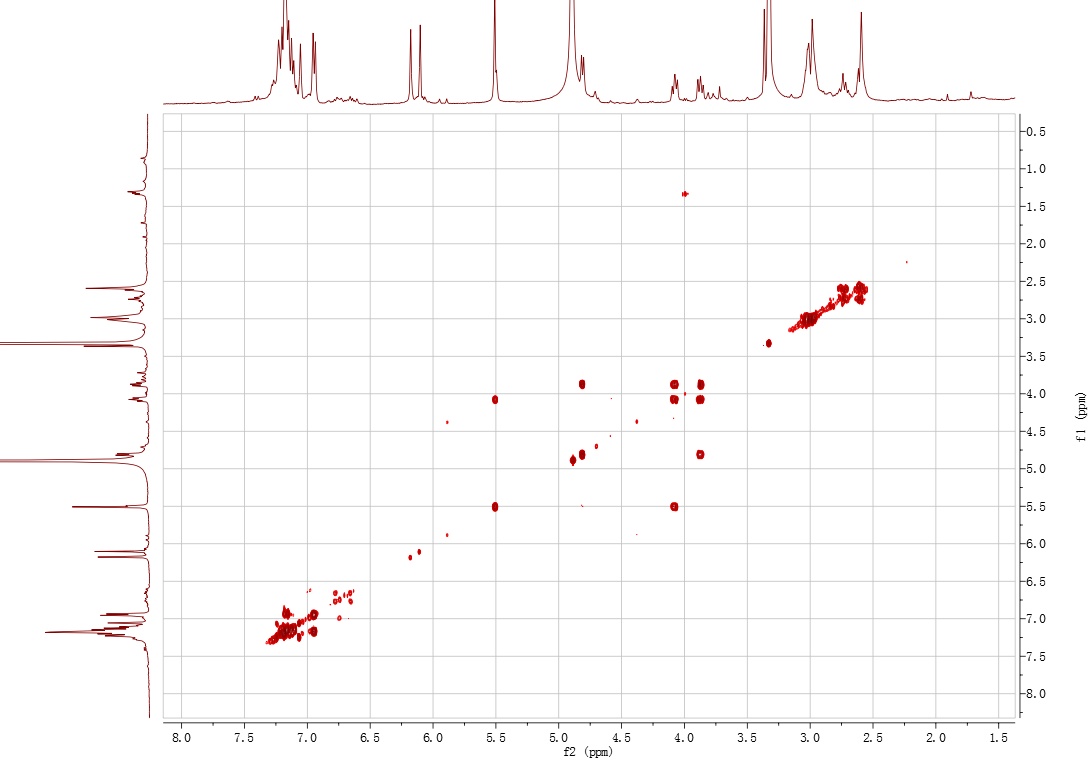


^1^H-^1^H COSY spectrum of compound **V** in methanol-*d*_4_


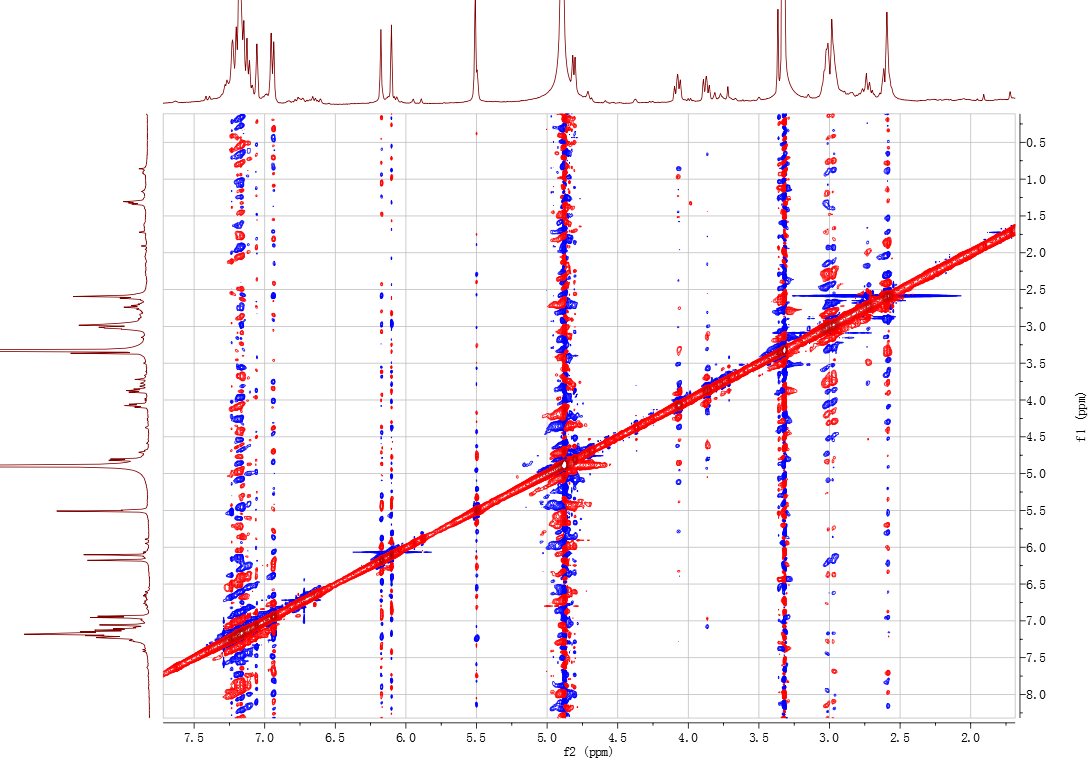


NOESY spectrum of compound **V** in methanol-*d*_4_

**References**

1. Konishi T, Konoshima T, Shimada Y, Kiyosawa S. Six new 2-(2-phenylethyl)chromones from agarwood. Chem Pharm Bull. 2002; 50: 419–422.
2. Yang DL, Mei WL, Zeng YB, Guo ZK, Zhao YX, Wang H, Zuo WJ, Dong WH, Wang QH, Dai HF. 2-(2-Phenylethyl)chromone derivatives in Chinese agarwood “Qi-Nan” from *Aquilaria sinensis*. Planta Med. 2013; 79: 1329–1334.
3. Ibrahim SRM. New 2-(2-phenylethyl)chromone derivatives from the seeds of *Cucumis melo* L var. *reticulatus*. Nat Prod Commun. 2010; 5: 403–406.
4. Shimada Y, Tominaga T, Konishi T, Kiyosawa S. Studies on the agarwood (Jinko). I. Structures of 2-(2-phenylethyl)chromone derivatives. Chem Pharm Bull. 1982; 30: 3791–3795.
5. Hashimoto K, Nakahara S, Inoue T, Sumida Y, Takahashi M, Masada Y. A new chromone from agarwood and pyrolysis products of chromone derivatives. Chem Pharm Bull. 1985; 33: 5088–5091.
6. Yang L, Qiao L, Xie D, Yuan YH, Chen NH, Dai JG, Guo SX. 2-(2-Phenylethyl)chromones from Chinese eaglewood. Phytochemistry. 2012; 76: 92–97.
7. Yang DL, Wang H, Guo ZK, Dong WH, Mei WL, Dai HF. A new 2-(2-phenylethyl)chromone derivative in Chinese Agarwood ′Qi-Nan′ from *Aquilaria sinensis*. J Asian Nat Prod Res. 2014; 16: 770–776.
8. Wu B, Kwon SW, Hwang GS, Park JH. Eight new 2-(2-phenylethyl)chromone (=2-(2-phenylethyl)-4H-1-benzopyran-4-one) derivatives from *Aquilaria malaccensis* agarwood. Helv Chim Acta. 2012; 95: 1657–1665.
9. Yang JS, Wang YL, Su YL. Studies on the chemical constituents of *Aquilaria sinensis* (Lour.) Gilg. IV. Isolation of 2(2-phenylethyl)chromone derivatives. Acta Pharm Sin. 1989; 24: 678–683.
10. Chen D, Xu ZR, Chai XY, Zeng KW, Jia YX, Bi QD, Ma ZZ, Tu PF. Nine 2-(2-phenylethyl)chromone derivatives from the resinous wood of Aquilaria sinensis and their inhibition of LPS-induced NO production in RAW 264.7 cells. Eur J Org Chem. 2012; 27: 5389–5397.
11. Iwagoe K, Konishi T, Kiyosawa S, Shimada Y, Miyahara K, Kawasaki T. Studies on the agalwood (Jinkō) VII. Structures of phenylethylchromone derivatives AH7, AH8 and AH9. Chem Pharm Bull. 1988; 36: 2417–2422.
12. Shimada Y, Konishi T, Kiyosawa S, Nishi M, Miyahara K, Kawasaki T. Studies on the agarwood (Jinkō). IV. Structures of 2-(2-phenylethyl)chromone derivatives, agarotetrol and isoagarotetrol. Chem Pharm Bull. 1986; 34: 2766–2773.
13. Konishi T, Iwagoe K, Sugimoto A, Kiyosawa S, Fujiwara Y, Shimada Y. Studies on agalwood Jinkō X. Structures of 2-(2-phenylethyl)chromone derivatives. Chem Pharm Bull. 1991; 39: 207–209.
14. Yagura T, Ito M, Kiuchi F, Honda G, Shimada Y. Four new 2-(2-phenylethyl)chromone derivatives from withered wood of *Aquilaria sinensis*. Chem Pharm Bull. 2003; 51: 560–564.
15. Zhao YM, Yang L, Kong FD, Dong WH, Li W, Chen HQ, Wang H, Cai CH, Gai CJ, Mei ML, Dai HF. Three new 5,6,7,8-tetrahydro-2-(2-phenylethyl)chromones and one new dimeric 2-(2-phenylethyl)chromone from agarwood of *Aquilaria crassna* pierre ex lecomte in laos. Nat Prod Res. 2021; 35: 2295–2302.
16. Li Y, Sheng N, Wang L, Li S, Chen J, Lai X. Analysis of 2-(2-phenylethyl)chromones by UPLC-ESI-QTOF-MS and multivariate statistical methods in wild and cultivated agarwood. Int J Mol Sci. 2016; 17(5): 771.
17. Iwagoe K, Kakae T, Konishi T, Kiyosawa S, Fujiwara Y, Shimada Y, Kawasaki T. Studies on the agalwood Jinkō VIII. structures of biphenylethylchromone derivatives. Chem Pharm Bull. 1989; 37: 124–128.
18. Huo HX, Zhu ZX, Song YL, Shi SP, Sun J, Sun H, Zhao YF, Zheng J, Ferreira D, J Zjawiony K, Tu PF, Li J. Anti-inflammatory dimeric 2-(2-phenylethyl)chromones from the resinous wood of *Aquilaria sinensis*. J Nat Prod. 2018; 81: 543–553.
19. Huo HX, Gu YF, Zhu ZX, Zhang YF, Chen XN, Guan PW, Shi SP, Song YL, Zhao YF, Tu PF, Li J. LC-MS-guided isolation of anti-inflamamtory 2-(2-phenylethyl)chromone dimers from Chinese agarwood (*Aquilaria sinensis*). Phytochemistry. 2019; 158: 46–55.

1. ***** Correspondence:

   Yuelin Song

   syltwc2005@163.com

   Jun Li

   drlj666@163.com

   *^a^ Modern Research Center for Traditional Chinese Medicine, Beijing Research Institute of Chinese Medicine, Beijing University of Chinese Medicine, Beijing 100029, People’s Republic of China.*

   *^b^ School of Chinese Materia Medica, Beijing University of Chinese Medicine, Beijing 100029, People’s Republic of China.* [↑](#footnote-ref-1)
